# Supplementary material for: Examining Food Sources and Their Interconnections over Time in Small Island Developing States: A Systematic Scoping Review
Source: Nutrients. 2025 Jul 18;17(14):2353. doi: 10.3390/nu17142353 (PMC12298424; doi:10.3390/nu17142353)
Supplement: Supplementary file 1 [file nutrients-17-02353-s001.zip › ASSIA database_search strategy.pdf]

Search strategy: ASSIA database

https://libguides.cam.ac.uk/az.php?a=m via Raven

ASSIA:

157 references retrieved on 17 June 2021 (no limits applied)

Combination of Keywords and Subject Heading (MAINSUBJECT)

- Advanced Search, Anywhere except full text - NOFT

A) Food Sources = a or b or c or d

a) General

noft((foodscape or "food environment?" or "food desert?" or "food swamp?" or "obesogenic environment?" or "nutrition* environment?" or "food forest?" or "food sourc*" or "market-based food?" or "marketbased food?" or "food purchas*" or "dietary pattern?" or "dietary behavio*r?" or "food consumption pattern?" or "food consumption behavio*r?" or "food acqui*" or "food choice?" or "food preference?")) OR mainsubject(("food consumption" or "food habits"))

b) Own production (1 or 2 or 3)

noft((commun* OR urban* OR rural* OR local* OR school* OR work* OR workpl* OR smallhold* OR small-hold*) NEAR/3 (allotment? or agricult* OR horticult* OR garden* OR farm* OR agroprocessing OR "agro processing" OR aquacultur* OR fishing OR fisheries OR fishery OR maricult* OR "food production"))

noft(((food? OR animal? OR fruit? OR vegetable? OR produce OR greens OR crop* OR insect? OR bees OR bird? OR nuts or plant* or honey) NEAR/3 ("own produc*" OR rear* OR forag* OR gather* OR harvest* OR hunt*)))

noft((Wild*) NEAR/1 (food? or plant*))

c) Purchase

noft(((enterprise? OR trading? OR trader? OR dealer? OR retailer? OR entrepreneur? OR vendor? OR street? OR school? OR college? OR hawker? or umbrella? or stall? or pallet? or shop? or kiosk? or store? or market? or parlour? or grocer* or truck? or van? or pick-up? or pickup? or trike? or bicycle? or bike? or tricycle? or wholesale? or bulk? or distributor? or takeaway? or take-away? or takeout? or take-out? or fast) NEAR/1 (food? or beverage? or fruit? or vegetable? or meal? or snack?)))

d) Food exchange and Food Aid (4 or 5 or 6)

noft((food? OR beverage? OR fruit? OR vegetable? OR meal?) NEAR/1 (transfer* OR borrow* OR exchang* OR barter* OR shar* OR aid* OR gift* OR bank* OR parcel* OR "faith-based organisation*" OR "shipp* barrel*"))

noft((tanda OR tandas OR "partner hand" OR partnerhand OR "box hand" OR boxhand OR ROSCAs OR rosca OR "food program*" OR "food kitchen?" OR "food sharing initiative?" OR "food network*" OR sou-sou OR susu OR asue OR feasting?))

noft(((commun* OR cultur* OR religio*) NEAR/1 (feast*)))

B) Small Island Developing States

noft((Small Island? Developing State? or SIDS? OR **Melanesia** OR **Micronesia** OR **Caribbean countries** or Caribbean? or Anguilla* OR Antigua* OR Antilles* OR Aruba* OR Bahamas* OR Barbuda* OR Barbados* OR Belize* OR Bermuda* OR Caicos* OR Caledonia* OR Cayman* OR Comoros* OR "Cook Island*" OR Cuba* OR Curacao* OR Dominica* OR Dominican* OR Fiji* OR

Grenada* OR Grenadines* OR Guadeloupe* OR Guam* OR Guinea-Bissau* OR Haiti* OR Jamaica* OR Kiribati* OR Lucia* OR Maarten* OR Maldives* OR Marshall* OR Martinique* OR Mauritius* OR Montserrat* OR Nauru* OR Nevis* OR Niue* OR Palau* OR Papua* OR Polynesia* OR Principe* OR Kitts* OR Samoa* OR Sao Tome* OR Seychelles* OR Singapore* OR Solomon* OR Suriname* OR Timor-Leste* OR Tonga* OR Trinidad* OR Tobago* OR Tokelau* OR Turks* OR Tuvalu* OR "Puerto Rico*" OR Marianas* OR Martinique* OR Vanuatu* OR Verde* OR Vincent* OR "Virgin Island*"))

Full search:
(A) AND (B)

It was impossible to combine all chunks together. The databases kept crashing.
I did run chunks from (A) individually AND (B)
