# Supplementary material for: Examining Food Sources and Their Interconnections over Time in Small Island Developing States: A Systematic Scoping Review
Source: Nutrients. 2025 Jul 18;17(14):2353. doi: 10.3390/nu17142353 (PMC12298424; doi:10.3390/nu17142353)
Supplement: Supplementary file 1 [file nutrients-17-02353-s001.zip › Conceptualisation of evidence_Disciplines.pdf]

Supplementary information Fig.5

Conceptualisation of evidence: Disciplines

Method: For each included study, we extracted data from the different reported departments from all authors involved in the research. For peer-reviewed articles, the disciplines were extracted using the author's affiliations. If the disciplines were not provided, we entered 'not reported' in the charting form. For grey literature documents, where disciplines were less often explicitly reported by authors, we used sources such as the department or publishing institution, the summary, and the reported Ministers attending a meeting or participating in a report to guide our decisions. If we could not infer the disciplines involved in the grey literature documents, we entered 'unclear' in the charting form. We used categorical variables and a pre-set list of disciplines created by the team review members following an inductive approach to guide the process. The pre-set list of disciplines was iterated and revised within the team at different stages. The extracted disciplines from 10% of the studies were double extracted by two independent members. Our analysis approach to grouping disciplines of evidence into categories was guided by thematic similarities observed in the data.

Categories:

1. **Food, Health and Well-being** It includes departments of Health, Emergency Medicine, Nursing, Epidemiology, Public Health, Mental Health, Sports and Nutrition, Exercise, Dietetics, Food and Nutrition, Food Systems, Food Technology and Food Safety.
2. **Agriculture and Land Studies** It includes departments of Agriculture, Agroecology, Forestry, Fisheries and Aquaculture, Marine Affairs, Land and Agrarian Studies, Rural infrastructure, Value Chains, Food Supply, Resource Management and Preservation, Land and Natural Conservation, Natural Resources and Applied Sciences and Entomology.
3. **Environmental Studies and Sustainability** It includes departments of Environmental Studies, Biological Diversity and Ecosystem Services, Plant Biology, Industry and Environment, Sustainability, Sustainable Development and Sustainable Consumption.
4. **Economics and finance** It includes departments of Finance, Economics, Business, Global Development, Policy and Governance, Legal, Trade and Market, Foreign Affairs and Marketing.

5. **Social Sciences and Humanities** It includes departments of History, Sociology, Anthropology, Archeology, People and Cultures, Geography, Human Geography, Social and Behavioural Sciences, Humanities and Political Sciences.
6. **Community and Development** It includes departments of Community Development, Architecture and Planning, Recreation and Tourism Administration, Social Protection, Disaster Emergency Management, Education and Psychology.
