# Supplementary material for: Examining Food Sources and Their Interconnections over Time in Small Island Developing States: A Systematic Scoping Review
Source: Nutrients. 2025 Jul 18;17(14):2353. doi: 10.3390/nu17142353 (PMC12298424; doi:10.3390/nu17142353)
Supplement: Supplementary file 1 [file nutrients-17-02353-s001.zip › Conceptualisation of evidence_Framing.pdf]

Supplementary information Fig.6

Conceptualisation of evidence: Framing

Method: For each included study, we extracted the main concept/s the research was framed around. As the research framing was often not explicitly reported by authors, we inferred it from the Introduction, Abstract or Keywords (in peer-reviewed studies), and from the Introduction or Executive Summary (in grey literature documents). When reported, we also used the theoretical framework to guide our decisions. If we could not infer the framing, we entered 'unclear' in the charting form. For studies that covered more than one frame, we limited the extraction to a maximum of three frames, the most mentioned. We used categorical variables and a pre-set list of frames created by the team review members following an inductive approach to guide the process. The pre-set list of frames was iterated and revised within the team at different stages. The extracted frames from 10% of the studies were double extracted by two independent members. Our analysis approach to grouping frames of evidence into categories was guided by thematic similarities observed in the data.

Categories:

1. **Sustainable Development and Resilience** It includes framings around SDG Agenda 2030, climate change resilience, biodiversity and ecosystem, natural disasters or emergencies (and recovery), SIDS particular challenges, food imports dependency, sustainable and local agrifood systems.
2. **Health and Disease** It includes women's health and safety, children's health and education, healthy aging, COVID-19 pandemic, Zero Hunger (SDG2) or undernutrition and hunger, and NCDs and malnutrition.
3. **Urban Sustainability and Social Spaces** It includes healthy cities, (rapid) population growth, globalization, gentrification and tourism, (rapid) urbanization, and sustainable urban agriculture.
4. **Food Security and Nutrition** It includes food and nutrition (in)security, food availability, food access, food supply, and food safety.
5. **Diet and Food Environments** It includes dietary patterns, food trends, food habits, food choices, nutrition transition, UPFs, obesogenic environments, food environments (incl. food retail modernisation), nutrition education, health promotion and hygiene.

6. **Economic Growth and Market Development** It includes (poor) economic growth, market development, agricultural sector development, human development, poor rural infrastructure, food loss.
7. **Social and Cultural Sustainability** It includes social economy, social sustainability, cultural heritage, kin relationships, social and cultural changes, legacies of colonialism, external aid consequences, informality in food systems, traditional indigenous knowledge (incl. wild foods).
8. **Equity and Justice** It includes gender equity, human mobility, social justice, (urban/rural) poverty, (food) inequities, right to food, food sovereignty, food agency.
