# Supplementary material for: Examining Food Sources and Their Interconnections over Time in Small Island Developing States: A Systematic Scoping Review
Source: Nutrients. 2025 Jul 18;17(14):2353. doi: 10.3390/nu17142353 (PMC12298424; doi:10.3390/nu17142353)
Supplement: Supplementary file 1 [file nutrients-17-02353-s001.zip › Eligibility criteria_iteration 1.pdf]

Inclusion criteria	Exclusion criteria
**Exposure:**<br>· It reports one or more types of food sources - purchased, own production (growing, gathering, hunting, fishing), borrowed, exchanged, bartered, food transfers and gifts, food aid, local-vs-imported<br><br>· Studies about small-scale farmers focusing on farmer's own food security, income and other livelihoods. Small-scale farming as a food source for own consumption.	**Exposure:**<br>· Studies reporting food-related issues (such as food transport and distribution, dietary and consumption patterns, food items) but not specifically linked to a food source.<br><br>· Studies describing food sector management (e.g., agriculture sector) and/or worker organisations (e.g., peasants' organisation)<br><br>· Studies about small-scale farmers focusing on how to increase food production, value chains, access to markets, etc.<br><br>· Food sources for non-human feeding or food practices (e.g., hunting) by non-humans<br><br>· Food sources (such as large commercial farms) not described as part of the food environment (i.e., there is no link or interaction with population)
**Population:**<br>UN categorisation of Small Island Developing States + Tokelau	
**Outcome:**<br>Information on food source characteristics and/or conceptualisation of evidence on food sources	
**Time Frame:**<br>From 1992 (included) up to date (Trade liberalization agreements, FAO)	**Time Frame:**<br>Studies published on or after 1992 but focusing on past times such as research using isotope analysis of skeletal elements to reconstruct indigenous diets.
**Languages:**<br>All languages	
**Study type:**<br>All included. No need to contain primary data. Reviews are also included	**Study type:**<br>Studies reporting hypothetical scenarios.
**Document type:**<br>Peer-reviewed journal articles and grey literature.	**Document type:**<br>Exclude books, book chapters and thesis (not relevant for mapping exercise).<br>Exclude conference proceedings and conference abstracts (not enough information to be extracted)
