# Supplementary material for: Examining Food Sources and Their Interconnections over Time in Small Island Developing States: A Systematic Scoping Review
Source: Nutrients. 2025 Jul 18;17(14):2353. doi: 10.3390/nu17142353 (PMC12298424; doi:10.3390/nu17142353)
Supplement: Supplementary file 1 [file nutrients-17-02353-s001.zip › Eligibility criteria_iteration 2.pdf]

Core Evidence – ScR

- **Objective 1: To describe the variety, extent and nature of the different food sources identified.**
- **Objective 2. To map the countries and settings from which the existing evidence is drawn**
- Objective 3. To underlie how the evidence has been conceptualised
- Objective 4. To identify a disciplinary framing of evidence and key element/s for future research on leverages for better diets, health, and food sovereignty

****ALL elements below must be met for a study to be considered 'core evidence' and to be included for data extraction**

**Variety** – 'what it is'	**Extent** – 'the range it covers'	**Nature** – 'the way it works'
Include studies **only if** focus on:	Include studies **only if** details are given on:	Include studies **only if** details are given on:
• Food sourcing<br>• Food acquisition<br>• Food purchasing	• Scale of use<br>• Frequency of use<br>• Popularity<br>• Relevancy	• Foods involved<br>• Function/role<br>• Who uses it?
Studies must:	Studies must:	Studies must:
• Report **at least one or more** food sources (own production, purchase, wild, BEB, aid)	• Give **at least one or more** details about the extent covered by the food source	• Give **at least two or more** details about the nature of the food source<br>• Give details about foods involved or who uses the food source **as well as** details on the role or function covered by the source
Exclude:	Exclude:	Exclude:
• Studies only about food production – no focus on food produced for own consumption<br>• Studies only about food consumption – no focus on the source of the food consumed	• Not enough information given	• Not enough information given
**Reason: Ineligible exposure**	**Reason: Ineligible outcome**	**Reason: Ineligible outcome**

Setting	Data-level	Document type	Timeframe
Include studies **only if** report on:	Include studies **only if** outcomes are given at:	Include studies **peer-reviewed and grey literature**:	Include studies **only if:**
Small Island Developing States or SIDS<br><br>Caribbean/Melanesia/Micronesia/French Polynesia/Commonwealth of the Northern Mariana Islands<br><br>American Samoa, Anguilla, Antigua and Barbuda, Antilles, Aruba, Bahamas, Bahrain, Barbados, Belize, Bermuda, Cabo Verde, Cayman Islands, Comoros, Cook Islands, Cuba, Curacao, Dominica, Dominican Republic, Fiji, Grenada, Guadeloupe, Guam, Guinea-Bissau, Haiti, Jamaica, Kiribati, Maldives, Marianas, Marshall Islands, Martinique, Mauritius, Montserrat, Nauru, New, Caledonia, Niue, Palau, Papua New Guinea, Puerto Rico, Saint Kitts and Nevis, Saint Lucia, Saint, Vincent and the Grenadines, Samoa, Sao Tomé and Principe, Seychelles, Singapore, Sint Maarten, Solomon Islands, Suriname, Timor-Leste, Tonga, Trinidad and Tobago, Tokelau , Turks and Caicos Islands, Tuvalu, Vanuatu, British Virgin Islands, U.S Virgin Islands	• Individual-level<br>• Household-level<br>• Community-level		• Published on or after 1992
Studies must:	Studies must:		
• Report **at least one** of the settings	• Report **at least one** of the levels<br>• Ultimately take the perspective of **the people** sourcing the food<br>• And must be people living in the setting as residents. Exclude tourist		
Exclude:	Exclude:	Exclude:	Exclude:
• Any other settings	• Outcomes given at country, regional, or global level<br>• Studies describing hypothetical scenarios	• Book, book chapters, thesis, conference proceedings, conference abstracts	• Studies published on or after 1992 but focusing on past times

Reason: Ineligible setting	Reason: Ineligible study type	Reason: Ineligible document type	Reason: Ineligible timeframe

Examples of potential functions/roles covered by the food source:

Nutrition and Diet diversity	Ethnic equity
Nutrition transition - Dietary shift	Social networks
NCDs	Social status
Food safety	Food cultures (norms)
Food supply	Animal welfare
Food for consumption	Water pollution
Food (and nutrition) security	Greenhouse emissions
Hunger alleviation	Deforestation
Burden of disease	Biodiversity
Employment	Environmental Sustainability
Income	Soil degradation
Economic growth	Weed control
Livelihoods	Food Waste
Transport	Domestic food production/consumption
Draught power	Imports dependency
Poverty and subsistence	COVID-19 impacts mitigation
Resilience against external shocks	Market opportunities for local farmers
Mental Health	Other – please, specify
Wellbeing	
Personal Security and safety	
School attendance	
Gender equity	
