# Supplementary material for: Examining Food Sources and Their Interconnections over Time in Small Island Developing States: A Systematic Scoping Review
Source: Nutrients. 2025 Jul 18;17(14):2353. doi: 10.3390/nu17142353 (PMC12298424; doi:10.3390/nu17142353)
Supplement: Supplementary file 1 [file nutrients-17-02353-s001.zip › EMBASE database_search strategy.pdf]

Search strategy: EMBASE database
1974 to 2021 June 16
https://libguides.cam.ac.uk/az.php?a=m via Raven

EMBASE:
1464 references retrieved on 28 June 2021 (no limits applied)

Combination of Keywords and EMTREE terms.

- Advanced Search, Keyword, Map Term to Subject Heading
- (mp.) 'multi-purpose': search fields of title, abstract, keywords, subject headings, original title, etc.

A) Food Sources = a *or* b *or* c *or* d

a) General
exp Food Preferences/ or (foodscape* or food environment* or food desert* or food swamp* or obesogenic environment* or nutrition* environment* or food forest* or food sourc* or market-based food* or marketbased food* or food purchas* or dietary pattern* or dietary behavio?r* or food consumption pattern* or food consumption behavio?r* or food acqui* or food choice* or food preference*).mp.

b) Own production (1 or 2 or 3)
1. ((commun* or urban* or rural* or local* or school* or work* or workpl* or smallhold* or small-hold*) adj3 (allotment* or agricult* or horticult* or garden* or farm* or agroprocessing or agro processing or aquacultur* or fishing or fisheries or fishery or maricult* or food production)).mp.

2. ((food* or animal* or fruit* or vegetable* or produce or greens or crop* or insect* or bees or bird* or nuts or plant* or honey) adj3 (own produc* or rear* or forag* or gather* or harvest* or hunt*)).mp.

3. (Wild* adj1 (food* or plant*)).mp.

c) Purchase
((enterprise* or trading* or trader* or dealer* or retailer* or entrepreneur* or vendor* or street* or school* or college* or hawker* or umbrella or umbrellas or stall or stalls or pallet or pallets or shop or shops or kiosk or kiosks or store or stores or market or markets or parlour or parlours or grocery or groceries or truck or trucks or van or vans or pick-up or pickup or pick-ups or pickups or trike* or bicycle* or bike* or tricycle* or wholesale* or bulk* or distributor* or takeaway* or take-away* or takeout* or take-out* or fast) adj1 (food* or beverage* or fruit* or vegetable* or meal* or snack*)).mp.

d) Food exchange and Food Aid (4 or 5 or 6)
4. ((food* or beverag* or meal* or fruit* or vegetable*) adj1 (transfer* or borrow* or exchang* or barter* or shar* or aid* or gift* or bank* or parcel* or faith-based organisation* or shipp* barrel*)).mp.

5. (tanda or tandas or partner hand or partnerhand or box hand or boxhand or ROSCAs or rosca or food program* or food kitchen* or food sharing initiative* or food network* or sou-sou or susu or asue or feasting).mp.

6. ((commun* or cultur* or religio*) adj1 feast*).mp.

B) Small Island Developing States

exp Caribbean/ or exp Melanesia/ or exp Federated States of Micronesia/ or (Small Island* Developing State* or SIDS* or Caribbean Region* or Melanesia* or Micronesia* or Polynesia* or Anguilla* or Antigua* or Antilles* or Aruba* or Bahamas* or Bahrain* or Barbuda* or Barbados* or Belize* or Bermuda* or Caicos* or Caledonia* or Cayman* or Comoros* or Cook Islands* or Cuba* or Curacao* or Dominica* or Dominican* or Fiji* or Grenada* or Grenadines* or Guadeloupe* or Guam* or Guinea-Bissau* or Haiti* or Jamaica* or Kiribati* or Lucia* or Maarten* or Maldives* or Marshall* or Martinique* or Mauritius* or Montserrat* or Nauru* or Nevis* or Niue* or Palau* or Papua* or Principe* or Kitts* or Samoa* or Sao Tome* or Seychelles* or Singapore* or Solomon* or Suriname* or Timor-Leste* or Tonga* or Trinidad* or Tobago* or Tokelau* or Turks* or Tuvalu* or Puerto Rico* or Marianas* or Martinique* or Vanuatu* or Verde* or Vincent* or Virgin Islands*).mp.

Full search:
(A) AND (B)
