# Supplementary material for: Examining Food Sources and Their Interconnections over Time in Small Island Developing States: A Systematic Scoping Review
Source: Nutrients. 2025 Jul 18;17(14):2353. doi: 10.3390/nu17142353 (PMC12298424; doi:10.3390/nu17142353)
Supplement: Supplementary file 1 [file nutrients-17-02353-s001.zip › Full description of food sources and interconnections.pdf]

# Supplementary Materials 6

## Table of contents

**AID** ........ **1**

NATURE ........ 1
*Type of foods* ........ *2*
*Role* ........ *2*
EXTENT ........ 4
*Scale of use* ........ *4*
*Relevant for who?* ........ *4*
CHANGES OVER TIME AND BETWEEN GENERATIONS ........ 5

**BUY** ........ **5**

NATURE ........ 5
*Type of foods* ........ *8*
*Role* ........ *10*
EXTENT ........ 13
*Scale of use* ........ *13*
*Relevant for who?* ........ *13*
CHANGES OVER TIME AND BETWEEN GENERATIONS ........ 16

**GROW** ........ **18**

NATURE ........ 18
*Type of foods* ........ *19*
*Role* ........ *21*
EXTENT ........ 23
*Scale of use* ........ *23*
*Relevant for who?* ........ *23*
CHANGES OVER TIME AND BETWEEN GENERATIONS ........ 24

**SHARE** ........ **25**

NATURE ........ 25
*Type of foods* ........ *29*
*Role* ........ *30*
EXTENT ........ 30
*Scale of use* ........ *30*
*Relevant for who?* ........ *31*
CHANGES OVER TIME AND BETWEEN GENERATIONS ........ 32

**STATE** ........ **33**

NATURE ........ 33
*Type of foods* ........ *35*
*Role* ........ *36*
EXTENT ........ 37
*Scale of use* ........ *37*
*Relevant for who?* ........ *37*
CHANGES OVER TIME AND BETWEEN GENERATIONS ........ 37

**WILD** ... **38**
NATURE ... 38
*Type of foods* ... *39*
*Role* ... *39*
EXTENT ... 40
*Scale of use* ... *40*
*Relevant for who?* ... *40*
CHANGES OVER TIME AND BETWEEN GENERATIONS ... 41
**INTERCONNECTIONS BETWEEN FOOD SOURCES** ... **41**
BUY (FROM) - REMIT (TO) ... 42
GROW (FROM) - MARKETPLACE (TO) ... 42
HOME GARDEN (FROM) - FEAST (TO) ... 42
FISH (FROM) - INFORMAL GROCERY (TO) ... 42
BUY (FROM) - BARTER (TO) ... 42
COMMUNITY FARM (FROM) - SCHOOL MEALS (TO) ... 42
HOME GARDEN (FROM) - GIFT (TO) ... 43
COMMUNITY (FROM) - ASSISTANCE (TO) ... 43
AID (FROM) - STATE (TO) ... 43
INFORMAL GROCERY (FROM) - FEAST (TO) ... 43
TARGET (FROM) - SCHOOL MEALS (TO) ... 43
HOME GARDEN (FROM) - INFORMAL GROCERY (TO) ... 44
WILD (FROM) - FEAST (TO) ... 44
GROW (FROM) - GIFT (TO) ... 44
WILD (FROM) - BUY (TO) ... 44
HOME GARDEN (FROM) - MARKETPLACE (TO) ... 44
FAMILY FARM (FROM) - GIFT (TO) ... 45
WILD (FROM) - BARTER (TO) ... 45
HOME GARDEN (FROM) - COMMUNITY FARM (TO) ... 45
COMMUNITY FARM (FROM) - GIFT (TO) ... 45
GROW (FROM) - FEAST (TO) ... 45
HOME GARDEN (FROM) - BARTER (TO) ... 45
FORAGE (FROM) - GROW (TO) ... 45
FISH (FROM) - SCHOOL MEALS (TO) ... 45
FISH (FROM) - BUY (TO) ... 45
FAMILY FARM (FROM) - BUY (TO) ... 46
BUY (FROM) - GIFT (TO) ... 46
GROW (FROM) - STATE (TO) ... 46
FISH (FROM) - GIFT (TO) ... 46
GROW (FROM) - AID (TO) ... 46
FAMILY FARM (FROM) - FEAST (TO) ... 47
FISH (FROM) - MARKETPLACE (TO) ... 47
FAMILY FARM (FROM) - MARKETPLACE (TO) ... 47
GROW (FROM) - INFORMAL GROCERY (TO) ... 47
FISH (FROM) - FEAST (TO) ... 47
FISH (FROM) - HOSPITALITY (TO) ... 47
FEAST (FROM) - STATE (TO) ... 47

GROW (FROM) - REMIT (TO)........................................................ 48
SHARE (FROM) - COMMUNITY (TO)........................................................ 48
COMMUNITY FARM (FROM) - MARKETPLACE (TO)........................................................ 48
HOME GARDEN (FROM) - BUY (TO)........................................................ 48
INFORMAL GROCERY (FROM) - GIFT (TO)........................................................ 48
MARKETPLACE (FROM) - FEAST (TO)........................................................ 48
REMIT (FROM) - GIFT (TO)........................................................ 48
GROCERY (FROM) - FEAST (TO)........................................................ 48
COMMUNITY FARM (FROM) - STATE (TO)........................................................ 48
FAMILY FARM (FROM) - INFORMAL GROCERY (TO)........................................................ 48

**REPORTED FOOD ITEMS BY FOOD GROUPS**........................................................ **50**

**REFERENCES**........................................................ **61**

Here we report all the analysed features concerning food sources found in the evidence reviewed, with the corresponding references in brackets.

# Aid

## Nature

### *Emergency*

Emergency food aid provided immediate assistance during extreme weather conditions or urgent situations. This aid was typically given by foreign governments, international organisations, humanitarian agencies, or philanthropic organisations [99, 15, 11, 12, 14]. Emergency food aid had its challenges to remain consistent or sufficient to cover the entire food shortage. There was often a gap between the end of aid and the availability of new crops after the disaster. Assessments of the impact of extreme events in emergency food aid programmes often overestimated the effects of more visible events like floods or droughts and overlooked smaller factors such as inadequate planting rates or excessive rainfall affecting root crops [15].

### *Target*

Target food aid supported existing national programmes and initiatives. It was provided by foreign donors such as governments, international organisations, and philanthropies [102, 103, 101, 100, 45, 13, 104]. An example was the Home-Grown School Meals (HGSM) program by the World Food Programme (WFP), which sourced food from local small-scale farmers to contribute to the local food supply [103]. However, challenges existed in ensuring these programs supported the local economy, were environmentally sustainable, and met recommended nutritional standards for school meals [100]. Some pilot projects had explored innovative approaches, such as distributing a mix of imported and locally produced food aid, which had been well-received, especially by school children [101]. These food aid programmes often targeted specific vulnerable groups, such as the Food Supplement Program implemented by WFP and USAID, which focused on individuals receiving care for HIV/AIDS and tuberculosis, malnourished pregnant and postnatal women, and undernourished children aged 0 to 5, providing monthly food assistance [13].

### *Community*

Community food aid was provided by NGOs, charities, social enterprises, churches, and voluntary groups [85, 48, 13, 47, 10]. These initiatives relied on volunteer donations, and referrals were based on need. Examples included breakfast programmes for children, home-delivered meals for the elderly, and meals provided by churches [47]. For instance, The Cornerstone Foundation, an NGO, operated a breakfast programme for approximately 87 children during the school year, with

referrals made by teachers based on need [47]. Churches also contributed to food assistance by hosting weekly meals, providing meals during holidays, and delivering food baskets to food-insecure individuals on an as-needed basis [47].

### *Type of foods*

#### *Emergency*

Emergency food aid packages commonly consisted of imported white rice, packet instant noodles, tinned meat, and tinned fish, supplemented with items like breakfast crackers, sugar, flour, and seeds for early production of fresh produce during the recovery phase [99, 15, 12, 11]. While the provision of shelf-stable foods ensured durability during transportation, recipients expressed dissatisfaction with the limited variety and absence of fresh food options. Concerns were raised regarding the inability to supplement their diets with fresh produce and the perceived unhealthiness of the provided food, which contradicted public health guidelines advising against excessive consumption of rice, tinned fish, and noodles. Consequently, the food aid did not fully align with participants' dietary preferences and failed to enable the preparation of balanced meals meeting nutritional guidelines [12].

#### *Target*

Target food aid for school feeding programs included a more diverse range of items, including beans, rice, peanut butter, oil, and daily hot meals with varied seasonal menus. These meals featured ingredients such as bulgur, pulses, fortified vegetable oil, micronutrient powder, and peanuts. Furthermore, school meals and take-home rations included canned mackerel fish from Japan, as well as beans, rice, vegetable oil, and salt from the United States [103, 101, 100, 13].

#### *Community*

In community food aid programs, a small food basket was provided, along with breakfast meals, rice and beans, chicken and coleslaw, and occasionally turkeys and hams [47].

### *Role*

#### *Emergency*

After a climate disaster, emergency food aid played a crucial role in helping people who were affected by hunger [99, 45, 48]. Villagers generally appreciated the support provided by food aid, but they often mentioned the lack of local food in the assistance they received. Following a cyclone, locally grown food was only available if it had been saved from before the disaster or collected from destroyed gardens

[15]. The provision of emergency food aid seemed to increase the availability of rice in people's diets, even after just one distribution. However, the impacts of climate extremes limited individuals' ability to choose the foods they preferred for a healthy and nutritious diet. This lack of agency undermined their ability to protect their health and well-being [11]. Families from outer islands expressed concerns about receiving only imported foods. Although these items provided access to foods they could not normally afford, people noted that they contradicted the messages they had learned in previous nutrition education workshops. Mothers, in particular, linked their consumption of unhealthy food from the aid to potential harm for their breastfeeding children. In nutrition education programs, nurses and health workers stress the importance of mothers eating well and consuming a "traditional" diet of local foods to maintain their children's health. Therefore, it was not surprising that post-cyclone food aid was considered inappropriate for breastfeeding mothers [12].

*Target*

Target food aid was reported to have several roles in supporting communities. Firstly, it ensured the provision of essential local public services [102] and offered support to government safety net programs, including the national school feeding program [103]. However, from a nutritional standpoint, this type of food aid had limitations. Although it included health-related activities and provided training on nutrition, the food basket lacked diversity, and the daily ration fell short of the targeted energy value outlined in the National Policy and Strategy on School Feeding [100]. In programs where a combination of local and imported food was distributed, communities were pleased with the opportunity to sell part of their local harvest, benefiting their children, and generating additional income. This was especially valuable in rural areas where limited access to markets possessed significant challenges for farmers [101]. Targeted food aid played a role in addressing short-term hunger, supplementing household food income, and even encouraging parents to send their daughters to school [101]. Targeted food aid granted individuals access to nutrient-rich food, which contributed to overall well-being [13, 104].

*Community*

Community food aid played a crucial role beyond simply providing food. They also contributed to increase social interaction and emotional support for those experiencing food insecurity. Moreover, they promoted education and public awareness about food insecurity, increased a sense of empathy, facilitated close relationships, and encouraged community engagement [85]. During the COVID-19 pandemic, NGOs and traditional communities had been working alongside the government to address the impacts of mitigation measures on farmers and

households. For instance, NGOs like FRIEND in Fiji, MORDI TT in Tonga, and KGA in Solomon Islands implemented projects that supported the distribution of seedlings, medicine, and farm equipment. They also conducted training sessions on cooking, food preservation, nutritional diets, and reducing NCDs for farmers and households, including women, men, and youth. Religious organisations provided relief supplies to households in need. Some churches offered their own land to households without access to land and mobilised their community-based organisations to address food insecurity. Well-organized programs, such as breakfast programs for school children, home-delivered meals for seniors, and food baskets for families provided by churches, had been established. However, these programs faced financial vulnerability, making it crucial to ensure sustainable funding [47].

**Extent**

***Scale of use***

After climate-related disasters, the reliance on food aid among villagers in rural and peri-urban areas was notable. These communities reported depending on food aid for a period of three to six months until their crops could regenerate [11]. However, the use of food aid was reported by only a small minority of respondents, with 5% in Fiji and 2% in SVG acknowledging its use. Interestingly, in both Fiji and SVG, the individuals who accessed food aid tended to be older and female, although the limited sample sizes hindered further analysis of this association [10]. These findings highlight the importance of food aid as a temporary support system in SIDS following climate disasters, particularly for vulnerable groups within the population.

***Relevant for who?***

*Emergency*

Emergency food aid proved especially relevant to individuals affected by climate extremes [12], particularly families who relied on agriculture and faced devastating losses, as their subsistence heavily relied on the produce from their gardens [99, 15].

*Target*

Many Pacific governments relied on target food aid to ensure the provision of essential public services [102]. The recipients of these programs primarily included individuals who suffered from severe malnutrition and disease [13, 104], as well as food-insecure primary school children, particularly those enrolled in public schools [101, 100]. However, it is important to note that while the programs successfully reached poor children, they fell short in reaching the most disadvantaged children

from the poorest and most vulnerable communities. This was because the selection criteria established by donors were often unmet by under-equipped schools, including those with rudimentary kitchens, in these highly vulnerable areas [101].

#### *Community*

Community food aid played a crucial role in supporting not only individuals facing food insecurity but also those experiencing feelings of loneliness and social isolation, regardless of their income status [85]. These organisations addressed not only the lack of food but also the emotional and social well-being of those in need.

### Changes over time and between generations

The provision of food aid led to notable transformations in dietary patterns over time and across generations [11, 12]. These changes were due to prolonged recovery periods after disasters for local crops, with yam yields taking 1-2 years to return to pre-drought levels, and persistent challenges in cultivating certain crops [11]. Consequently, food preferences and habits gradually shifted from reliance on local foods to increased consumption of imported rice and store-bought food items [11, 12].

## Buy

### Nature

#### *Informal grocery*

Informal grocery, including street-food vendors, corner shops, food trucks, *colmados*, small shops, roadside stationary or itinerant stalls, consisted of suppliers, transporters, retailers, and street and market vendors. It enhanced food accessibility and affordability, especially in low-income areas [42]. It was comprised of small independent or family-owned businesses that often procured goods from wholesalers [107]. Despite being less well stocked and often having higher prices, these local stores had the advantage not just of proximity but also of offering monthly credit facilities [32]. Informal grocery tended to concentrate at the main centres of commercial activity where potential clients required food during the working day. Mobile food vendors gathered near transportation hubs, schools, and offices, providing convenient access to food and saving consumers time and transportation costs. Operated with minimal or low capital investment, informal grocery exemplified flexibility and adaptability [105]. Examples of informal grocery included food trucks, small pick-up trucks equipped with additional speakers to announce their arrival, offering water, fruits, vegetables, and coffee [110]. Another example was the small corner stores (usually no more than 15 × 30 meters),

resembling bodegas, often offering bulk vegetables and sometimes fruits, typically sold by unit instead of weight. The corner stores could transform into lively dancehalls in the evenings, where patrons gathered to socialise, dance, and enjoy drinks. They offered fixed prices but extended credit to customers, expecting payment at the end of the month, even if partial [23, 110]. Another type of informal grocery involved boats reaching inhabited islands every few weeks, providing fresh produce otherwise inaccessible to many islanders at affordable prices. However, this trade was unregulated, and therefore lacked formal health and safety protection. For this reason, the more financially well-off islanders avoided sourcing their fresh foods in this way, whereas for many, it was their only source of fresh fruits and vegetables [19, 27]. Informal grocery allowed a direct exchange between the producer and the consumer, with fresh produce available within 5 km of production units and throughout urban neighbourhoods [111]. Small-scale commercial fishers sold their catches roadside from insulated ice boxes or at small commercial fish markets, without formal processing. This was convenient for people on the many mini-buses, taxis, and private vehicles that used the highway fronting the market area [71]. Roadside stalls and corner shops, which tended to be, on average, less than a kilometre from the patrons' homes, were visited frequently and generally accessed on foot [22]. Roadside markets, stationary or itinerant stalls on the side of the road, were scattered around the island and operated privately as re-sellers [115, 71, 105]. Roadside vendors, often consisting of simple wooden stands or carts, served customers passing by, including those traveling on buses or private vehicles [118]. Food vendors, including small kiosks and mobile sellers, gathered in the immediate surroundings of schools and libraries [112, 49].

### *Grocery*

Grocery as a food source included modern grocery stores, hypermarkets, supermarkets, big-box style supermarkets, and convenience stores, many of which are owned by transnational corporations [16]. Some supermarkets, part of chains and supplied by wholesaler marketers, purchase fresh items either from formal marketplaces or directly from farmers. They are increasingly capturing a larger market share for fresh produce that meets food safety standards and is quality-controlled. However, prices are significantly higher. Large food outlets, like "cash and carry" wholesale membership hypermarkets sell a wide range of non-food items as well as frozen and fresh food items [105]. Food and other items from these large food outlets seem to be purchased less often. This potentially suggests the practice of monthly bulk purchase for many commodities especially among households, possibly influenced by large wholesale/retail membership clubs offering discounts for bulk purchases [42]. Public transport, mainly buses, is the preferred mode of transport to supermarkets [22].

### *Marketplaces*

Described in the literature as central, domestic, local, municipal, town, city or formal markets. These markets included wet markets, fish markets and farmers' markets and consisted of stalls with produce laid out on the floor or benches [118]. They faced various challenges, including derelict structures, poor drainage, inadequate garbage disposal, and illicit dumping, as well as insufficient implementation of sanitary procedures and lack of running water, posing significant food safety risks to residents and tourists [105]. Women vendors often experienced sexual harassment, violence, and extortion, leading to their displacement from market premises to less safe locations like busy roadsides or open sewage areas, thereby increasing their vulnerability to violence. Moreover, inadequate storage facilities and toilets raised security and health concerns [121]. Efforts had been reported to improve market conditions by better managing human waste, solid waste, washing facilities, drainage, human security, and occupational health practices for cleaning staff, as well as enhancing safety with adequate lighting, wide access routes, information signage, and improved visibility [119, 120]. During the COVID-19 pandemic, these markets faced significant restrictions or closure, limiting people's access to local produce, including fruits, vegetables, and fish [124, 48].

From a food sourcing and production standpoint, there is little difference between products sold at wet markets and supermarkets, as both source from the same wholesale providers and distributors, often importing similar products from the government. However, the meat, fish, fruits, and vegetables sold at wet markets are perceived as fresher, less processed, and healthier due to the presentation of unpackaged food and the perceived knowledge and personal attention of vendors. Wet markets typically consist of open-air, partially sheltered spaces with numerous vendors selling fresh food products, spices, and other goods. The term "wet market" comes from the practice of regularly hosing down and washing the market floors to maintain cleanliness. These markets are often hot, humid, and noisy, with a labyrinth of stalls along narrow aisles and slippery floors [p54].

### *Hospitality*

It included hotels, restaurants, kava bars, hawker centres, food courts, takeaways, coffee shop stalls, and corporate fast-food chains. Hawker centres are open-air food complexes with multiple vendors selling local prepared foods. Coffee shop stalls are similar but usually have fewer stalls, typically under 20. Food courts, found in larger shopping malls, are air-conditioned versions of hawker centres. Since the types of foods sold at these venues are similar, the evidence refers to all of them as hawker centres [126]. These establishments serve as eat-and-leave spots, where youths usually have no intention of hanging around due to the environment, while the elderly often find them to be affordable and convenient places to socialise [125]. Fast-food outlets offer limited menus prepared quickly, where customers order, pay, and

pick up from a counter [16, 126]. Coffeehouses, including international chains and local brands, have expanded their offerings beyond coffee and tea to include bistros serving beer and a variety of set meals, catering to modern consumers' preferences [125].

### *Non-grocery*

It included non-grocery retailers such as pharmacy, bookstore, gas station, internet retail, and door-to-door retail. They offered ultra-processed food and drink products, but these are very low compared to USA and Canada. Online retail was of very little importance [16, 43, 117] and was primarily used during COVID by the at-risk population to avoid crowds in stores.

## *Type of foods*

### *Informal grocery*

Street-food vendors used to offer a diverse range of items, with local fruits and vegetables, beverages, ground provisions, and packaged snacks being among the most traded goods. Additionally, some vendors used to supply imported food sourced from local importers [113]. Fresh fish was the only product sold mainly by street vendors [42]. Water delivery used to occur at least twice a week by trucks, while daily visits from produce trucks used to ensure a reliable supply of fruits and vegetables in rural villages [110]. In small stores, various ultra-processed products such as carbonated soft drinks, snacks, confectionery, and ice cream used to be prominently sold, alongside imported processed food. [16, 67, 118, 116].
Some households used to rely entirely on corner shops for their purchases, with white bread and sweets/chocolates being particularly popular items [42]. Small, independent supermarkets, mainly concentrated in low-income areas, used to sell convenience items, processed foods, and limited fresh produce [105]. *Colmados* used to offer a variety of fresh produce and Caribbean vegetables, as well as bulk products like rice and beans. Homemade foods, canned/industrially processed items, local rum, sweet wines, and vacuum-packed cartons of milk and juices were also part of their inventory. Additionally, *colmados* used to stock individually wrapped products such as vanilla extract, cocoa powder, spices, and chicken bouillon. Cold drinks, including local and international soft drinks, litter bottles of local beer, and five-gallon jugs of potable drinking water, used to be available for purchase. Some *colmados* used to offer meat, often butchering it on-site for custom orders [23, 110].
Traditional markets used to dominate the fresh and produce-based product categories, with fish markets playing a significant role in the sale of fresh fish varieties. Roadside stalls used to provide a surprisingly diverse range of foodstuffs, ranking second only to supermarkets in poultry sales and offering products like fresh milk, eggplant, and certain types of seafood [22]. Small neighbourhood stores

mainly fulfilled immediate consumption needs for daily meals (sauce, canned foods, instant coffee, a packet of rice) and snack foods (sweets, cakes and pastries, drinks) [32].

### *Grocery*

The least affluent families established a budget for their supermarket visits, with women carefully monitoring their purchases to manage expenses. Typically, these shopping trips amounted to between €150 and €250, with shopping trolleys overflowing with frozen meat or poultry, large bags of rice, frozen vegetables, and bulk-sized packages of butter, oil, sugar, and mayonnaise [32]. Supermarkets predominantly stocked imported goods, with items like savoury snacks, processed meats, seafood, ready-made meals, processed fruits and vegetables, sweets, and biscuits experiencing significant demand [107]. Commonly found items included wheat, maize, rice, chicken meat, non-alcoholic beverages, soybean oil, refined sugar, raw centrifugal sugar, and palm oil [106], as well as ready-meals, spreads, sauces, breakfast cereals, and biscuits (cookies) [16]. These stores offered a wide array of cereal products, canned foods, and processed food such as rice, sugar, cooking oil, canned meat, powdered milk, cornmeal, and fresh vegetables and fruits [42]. Additionally, they stocked rice, noodles, and tin taiyo [33], as well as eggs and meats [67], pre-packaged items [23], and rice and chicken in bulk [108], or simply rice and chicken [18]. There were two varieties of chicken available: less expensive fatty legs imported from the USA, and mixed pieces (wings, drumsticks, thighs) imported from New Zealand, which were slightly leaner but pricier. The store sold three times more containers of the fatty legs in one and a half months compared to the mixed pieces [18]. While consumers predominantly favored supermarkets for processed and manufactured food products, they opted for alternative outlets for most of their fresh produce [22]. Food vendors at schools offered a limited selection of inexpensive snacks, including various types of cookies, sweet or savory fried dough, bread, fried bananas, instant noodles (often consumed dry), fruit-flavored drinks [41], as well as processed foods (chips, cookies, crackers, ice cream, popcorn, sweets, and sweet starch), fried foods (banana, dough, horse meat, hot dog), prepared foods (bread with peanut butter, hard-boiled eggs, boiled manioc, rice and beans/peas, sweet potatoes with milk, roasted peanuts), fruit (bananas and mangoes), frozen fruit juice, sugar-sweetened beverages (fruit drinks, shaved ice with syrup), and coffee [112]. Other offerings included cakes and drinks, keke (deep-fried pancakes), or dried noodles [49], as well as sweets, syrup drinks, and snacks like Cheese Trix (artificially flavored puffed corn), crackers, popcorn, or biscuits [70], and soda, candy, chips, and burritos [47], or sugar-sweetened beverages, lollies, candy, confectionery, fried beans, and potato chips [67].

### *Marketplaces*

Local fresh starchy fruits and vegetables, along with white buns, fresh fish, crab, nuts, popcorn, coconut buns, cinnamon donuts, and ready meals such as fried fish, cassava pudding, taro leaf pudding, fish and chips, fried rice balls, fish pudding, and sausage with cabbage and rice products, were all available [118]. Many women, who had access to markets, would gather fish, octopus, and shellfish, including giant clams, to prepare traditional puddings covered in coconut cream, thus creating value-added products for sale in municipal markets [71]. Formal markets served as the primary source for roots and tubers, fresh vegetables, fresh fruit, and green bananas and breadfruit [42]. The most common places to purchase food varied, with fresh fruit and vegetables bought from fruit and vegetable markets, starchy vegetables from markets, and fish and seafood from the fish market [67].

#### *Hospitality*

Kava bars also used to offer traditional fish puddings covered in coconut cream made with locally caught fresh fish [71]. For urban dwellers, street stalls were the most common places to purchase cooked seafood and finfish [67]. Cooked food, primarily obtained from takeaways and food bars, included items like patties, sow bow, pies, cooked chicken, cooked fish, sausages, and beverages [42, 118]. Hawker centres served cooked food such as mixed rice dishes like fried rice, coconut-rice ("nasi lemak"), and biryani, noodle dishes, stir-fried vegetables, legumes, soy, poultry, meat, and fish dishes. Local vendors appeared to offer a wider variety of foods from different food groups, including legumes and green leafy vegetables, and employed various cooking methods such as steaming and stir-frying [126]. Foods served at fast-food Western chains were similar to those available internationally but might also feature variations to suit local taste preferences. These foods were highly appealing, reasonably priced, and marketed effectively, although they tended to be energy-dense and lacking in nutrients [126].

### ***Role***

#### *Informal grocery*

Informal grocery played a crucial role in ensuring food security for poor urban households. Vendors adapted to the socio-economic conditions of their clientele by selling food in smaller portions, thus making goods more affordable. Also, they adopted customer-friendly practices to cultivate loyalty by including extra portions at no charge or by providing credit [113, 42, 23]. Establishing social relationships based on borrowing and repaying in-store credit created positive social ties, while failing to repay or withholding loans from credible shoppers could break down these relationships [23]. In periods of economic crisis, the decline in purchasing power and fall in employment opportunities in the formal sector often spur the

development of informal grocery as this provides employment and income to households in difficulty [105]. *Colmados* served as significant cultural hubs where people gathered socially and economically [23]. Roadside vendors played a vital role in improving food access for residents, eliminating the need to travel long distances to distant main markets for fresh produce [108, 114]. During the COVID-19 pandemic, an increasing number of families turned to selling vegetables and fruits at markets and roadside locations to generate income [108]. Informal grocery allowed people to access products for private consumption such as seafood (lobster) which is caught by local fishermen and by law only intended for tourism and export. However, social networks are crucial to have access to these items [66, 19]. School food vendors contributed to the increased availability of unhealthy food options, with purchasing from these vendors associated with child overweight [112]. During the pandemic, mobile food trucks were promoted to sell local food, offering market opportunities for local farmers, enhancing food access, and reducing dependency on food imports [59].

#### *Grocery*

Frequent consumption of food sourced from supermarkets was linked to positive aspects of diet, including higher reported intakes of fruits and vegetables and greater dietary diversity. However, it was also associated with increased weekly consumption of sugar-sweetened beverages (SSBs) and red and processed meat [10]. Looking beyond the practical aspect and the range of foods available, food buying is regarded as an indicator of status: an act that demonstrates possession of monetary resources, a sign of belonging to "the modern" society—as distinct from the "old times" when people had to work the land and be content with eating the produce of land and sea. These days, monetary dependency is regarded as more gratifying than dependence on working the land [32]. As incomes rise, so does the tendency to purchase imported foods from modern grocery stores. This trend is driven not only by the extended shelf life and lower production costs of imported items, which help improve access and address energy needs, especially during periods of high food insecurity, but also by their convenience in preparation and perceived association with modernity and affluence, as well as the aspiration towards a Western dietary pattern [20]. Occasional disruptions to the schedule of the international cargo ship that brings imported food sometimes cause modern grocery stores to run out of staple imported food items such as rice [18]. During lockdowns, supermarkets experienced food shortages and implemented restrictions on the quantity of items individuals could purchase, highlighting the vulnerability of this food source to global factors [108].

#### *Marketplaces*

Marketplaces served as bridges to the recent past and resilient places during the rapid transformations of the present. They provided, therefore, a social space of stability in a quickly changing world. People felt connected to them because they reminded them of the old days and helped them feel stable in the middle of social changes. People liked wet markets because they felt connected to their culture and traditions, especially as the government made big changes. Wet markets were kind of like a way for people to push back against all the big changes happening around them. They helped people feel like they still had something familiar in their lives, even as everything else was changing. Shoppers, vendors and visitors alike tended to describe markets in terms of their experiences rather than focusing simply on the variety of foods for sale. Wet markets figured as the primary places to meet and socialise as they provided spontaneous to sustained forms of social interaction, social mixing across a diverse range of groups and the creation of social ties that generate familiarity, loyalty and commitment outside conventional kin relations. The ability to touch, smell and sometimes taste food enhances the experience of shopping and reinforces trust among shoppers. During food scares (e.g. reports of 'bad' meat or fish or poor harvests) or scams (e.g. frozen meats passed off to consumers as fresh), customers seek out the assurances of a familiar and reliable vendor [25]. Class appearances matter less in the market than in the supermarket, where individuals with low incomes tend to feel judged by their dress. These places were perceived as 'great social equalisers' [p54, 84].

### *Hospitality*

The design of hawker centres, offering consumers a wide variety of cuisine styles under one roof, was often cited as a reason for choosing to eat there. Convenience, both in terms of affordability and ease of access, played a significant role in the decision to dine at hawker centres. Some women emphasised the convenience of eating out, especially given busy work schedules and time constraints. Home cooking was seen as challenging while working but became more feasible once women stopped working [126]. The growth of transnational fast-food chains of Western origin has raised public health concerns. Consumption of Western style fast-foods have been associated with abdominal obesity, type-2 diabetes, and coronary heart disease [126]. Western fast-foods were perceived as convenient because of their easy accessibility, late operating hours, and the availability of home-delivery option which was not offered by hawker centres. These foods were seen by some women as contributing to the culinary diversity of food choices particularly favoured by their children [126].

**Extent**

***Scale of use***

*Informal grocery*

High level of dependency on purchasing food from street vendors and corner shops underscored the importance of the informal food sector in the city [42]. In the Caribbean region, buying food from street vendors represented between 20 and 30 percent of urban family spending [56]. Sixty-six percent of respondents patronized corner shops over the past year [42]. The majority of food shopping was carried out in corner stores [23, 110]. Small wholesalers were popular choices for people living within the neighbourhood or within walking distance. In general, most food items were purchased at least once a week from wholesalers [42].

*Marketplaces*

Produce markets remained an important intermediary for fish, fruit and vegetables compared to supermarkets and roadside stalls [122, 105] retaining a substantial share of urban household food expenditure. Households used traditional markets not far below supermarkets, often visiting at least once a week. This suggested that urban consumers made use of both modern and traditional outlets for their food purchases, with traditional markets still largely preferred for fresh fruit and vegetables.

*Hospitality*

Singapore has a long tradition of hawkers like many of its South-East Asian neighbours. There was a high eating-out frequency usually eating either breakfast, lunch or dinner at eateries at these venues [126]. The major Western food chains in Singapore in 2015 were McDonald's® and Kentucky Fried Chicken® which together accounted for over 55% of the food service value for chained fast-food restaurants [126].

***Relevant for who?***

*Informal grocery*

Women played a critical role in the production, distribution, preparation, and sale of food in the Pacific. Most street-food vendors were women [102]. Street vending served as an important source of income and employment for women, who represented between 105 and 90 percent of vendors in the Caribbean region [56]. The informal food sector, comprising street-food vendors, was crucial for the food security of impoverished urban households [113]. Colmados and food trucks served as primary food resources for households in rural villages lacking reliable

transportation to town [110]. Corner shops were concentrated at major road intersections and scattered close to or within areas of informal and formal low-income housing [105]. Typically, poorer areas were more commonly served by produce markets and corner shops [42]. Boats reached inhabited islands approximately every two weeks, sometimes even once a month, from the main island, selling various fresh fruits and vegetables that were often unavailable or inaccessible to many islanders at affordable prices. While this boat provided access to fresh produce, the trade remained unregulated, lacking formal health and safety protections. Consequently, financially well-off islanders tended to avoid sourcing fresh foods this way, while for many others, it was their only option for fresh fruits and vegetables [19]. Studies indicated that women primarily owned and managed small catering operations and sold street food. They possessed traditional skills in these activities, already had the necessary cooking utensils at home, and the food they prepared also allowed them to feed their families at a lower cost [105]. Snacks purchased in and around schools, particularly during recess and on the way to and from school, were highly valued by adolescents. This served as their opportunity to assert their decision-making power regarding food consumption [41]. Most children who did not participate in lunch programs purchased food from street vendors or shops [70].

### *Grocery*

The preference among higher-income households for supermarkets was likely influenced by their relatively lower concern for differences in the cost of goods between supermarkets and other sources such as formal markets and vendors. Convenience appeared to be a greater consideration than cost, especially considering that many other items were sourced from supermarkets. Respondents in higher-income brackets relied more heavily on supermarkets, which were typically located in middle-class neighbourhoods, resulting in distinct spatial patterns [42]. Supermarkets attracted expats or tourists, as well as many residents, due to the lower prices of goods compared to *colmados*, where daily portions were purchased at higher prices. However, purchasing larger quantities of items found in supermarkets required a significant amount of money [23]. Higher-income households tended to allocate more of their budget to purchasing fresh fruit and vegetables from supermarkets [22].

### *Marketplaces*

Rural-based women fishers often sold their catch in municipal markets [71]. Produce markets tended to be more popular among lower-income earners but were extensively used by the general population [42]. Markets served as significant

economic and trading hubs, with women and girls playing central roles [120]. Market vendors were predominantly women, many of whom were extremely poor and relied on selling vegetables for daily subsistence [121]. For older retirees, especially, the relationships formed with wet market vendors provided feelings of social inclusiveness and combated social isolation. They described their connections as long-term and meaningful, fostering friendly and personal interactions, and serving as necessary links to community life [p54].

The fact that urban and humanitarian planning practitioners tend to not understand or to disregard the need to support existing food retail infrastructure may be related to the lack of evidence on how this infrastructure functions and is affected by crises. For most low-income city dwellers, access to and provision of food are relational in nature. They are influenced not only by the physical location in which trading activities occur (the physical infrastructure), but also by the interpersonal relationships among traders and between traders and customers (the social infrastructure). Several low- and middle- income countries have supported food retail modernisation policies aimed at the transformation of traditional and largely informal retailers to modern food systems with supermarkets as one of its most prominent features. Nevertheless, several studies demonstrate the limitations of supermarket-focused strategies because they disregard or undermine the social attributes of informal markets that are already present in these neighbourhoods. Advantages are found in the possibilities of negotiating small quantities and buying on credit. For most low- income city dwellers, food retail has an inherent social dimension and is indeed similar to Simone's concept of "people as infrastructure", a notion of an urban service that is directly linked to what people can produce with very few resources. Food provision and access, as social infrastructure, are generally at odds with official regulations and are highly provisional because of the extensive labour required, the rich social networks involved, and the numerous challenges faced by providers and users to access and maintain urban services [84, p54].

### *Hospitality*

Regular consumption of food from takeaways and restaurants was associated with younger age [10]. Besides simply relaxing and socializing in coffeehouses, these establishments also served as popular venues for youth to hold project meetings and discussions. Unlike adults, who often have their own offices, many students have traditionally used public spaces like these for studying or collaborating on projects.

One aspect that may not be immediately apparent to researchers outside the youth community is what the author term the 'Extended Family Syndrome'. Since the successful 'Stop at 2' campaign by the Singapore Government decades ago, many families—excluding perhaps Malay families—have had increasingly small family

units. As a result, many youth today grow up in environments where they are either only children or have just one sibling. This situation creates an emotional need for belonging within a larger group, typically their friends, classmates, and peers they frequently spend time with. This need for belonging and identity has led to the formation of tribes, associations, and various informal organizations where they identify themselves with a common name and often establish an online presence, such as a blog. This shared identity typically persists until the group of friends moves on to different classes or pursuits in life [125].

**Changes over time and between generations**

Over time, there has been a noticeable shift in food sourcing patterns, particularly between rural and urban populations. Urban areas have witnessed a transition from traditional, wild, or self-cultivated agri-foods to a reliance on purchased and ultra-processed foods [26]. This shift is primarily driven by the increased availability of imported, processed foods in stores, which often lack essential nutrients. Residents express frustration over this dependence on imported foods and raise concerns about their quality and origins, noting the shift from a time when acquiring food primarily required time and energy to now needing money. Imported options like high-fat chicken legs and carbohydrate-rich white rice dominate meals due to their affordability and accessibility, leading to meals that may lack sufficient nutrients. Despite preferences for locally sourced foods like fish, economic constraints force households to prioritize cheaper options such tinned fish [18, 19, 20].

There is a discernible shift toward more sales of ultra-processed products from modern grocery stores—mostly supermarkets and hypermarkets—at the expense of traditional stores. Other than ice cream, ultra-processed products are being increasingly sold through modern grocery stores. The biggest increase in sales of ultra-processed products in modern grocery stores are of juices, confectionery and snack bars [16]. While traditional markets (rural and urban) remain important for purchasing a variety of local foodstuffs (such as fresh fruit, vegetables and certain types of meat), over the years, it has become apparent that supermarkets have taken over a significant percentage of the market, with a mix of local, imported, processed and fresh food products. Modern supermarkets have overtaken traditional markets as the dominant food retail outlet. However, urban food expenditures are still shared between supermarkets and traditional main markets. This is highly significant, particularly considering the historically traditional-market food economy. In a general sense, supermarkets offer a full range of food products, but dominate the sales in the processed food category, while the traditional main market is still the preferred choice for fresh produce, such as fresh fruit, vegetables, and certain types of meat [22]. With more frequent climate extremes, coupled with the increased

availability and access to store-bought foods (it refers to a major supermarket chain and smaller stores with cheap, imported products), villagers described ceasing to cultivate certain crops, such as yams, and decreased motivation to continue tending gardens [11]. However, despite the convenience of supermarkets, some communities still prefer local corner stores (*colmados*), highlighting cultural preferences and benefits derived from these traditional shopping venues [23].

Over the past four decades, there has been a significant expansion and modernisation of the retail distribution system, leading to increased access to imported products. However, this convenience has resulted in a decline in the consumption of traditional plant foods like taro and tubers, in favour of easily accessible options like rice. This shift is also evident in school canteens, where European-style dishes have become prevalent, further perpetuating dietary changes. Consequently, the society faces issues of inequality and dependence on imported goods, with only a quarter of agricultural land being properly cultivated and self-sufficiency levels at a mere 20%. This development has contributed to an economy characterised by over-consumption and an inflated public sector, exacerbating difficulties in accessing local products due to their limited availability in supermarkets [32].

In response to the growing demand for high-value and processed foods, there has been a rapid evolution in agricultural supply chains and the rise of supermarkets. These changes involve a shift towards more integrated supply chains with fewer intermediaries and longer distances. While supermarkets offer potential benefits such as higher prices and larger markets for smallholder producers, they also pose challenges. Smallholders face limitations in meeting the demand for consistent supply due to small landholdings scattered across remote rural areas with poor infrastructure. Additionally, constraints such as limited access to inputs, technologies, marketing facilities, and credit make it difficult for them to maintain stringent quality and safety standards. These challenges are compounded by decades of poor farming practices and the adverse effects of climate change, including land degradation, declining soil quality, water scarcity, and pollution. The precariousness of land and natural resource rights further exacerbates the situation, discouraging farmers from investing in sustainable management practices [17].

Younger individuals, particularly those under the age of 35, have limited exposure to wet markets and exhibit a preference for the convenience offered by modern supermarkets. However, despite this preference, there remains a strong attachment to the preservation of wet markets among this group [p54].

Between 2000 and 2013, there was a significant surge in per capita fast-food purchases in the Dominican Republic [16]. Singapore, known for its Westernised

society compared to other Asian countries, has attracted numerous fast-food chains from the USA and Europe. These chains often choose Singapore as their initial expansion market in Asia, exposing various ethnic groups in Singapore to the fast-food culture. Interestingly, unlike in the USA, where fast-food consumption is common, in Singapore, visiting a fast-food establishment is considered fashionable, especially among the youth. This trend mirrors the cultural shift observed in the USA during the late 1950s and early 1960s, during the initial stages of fast-food chain proliferation [24].

## Grow

### Nature

#### *Home garden*

The literature also referred to home gardens as urban, village, backyard, kitchen, domestic, and sup-sup gardens. Home gardening was reported to have taken place in both urban and rural areas, typically within the gardens adjacent to residences or in nearby areas, close to houses and coastlines. These gardens were considered backyard crops, requiring less labour and perceived as of lower value, primarily serving household and individual consumption needs. The plots varied in size but were often small, such as backyard gardens, with some urban areas utilising containers on rooftops and balconies for food production. Surplus produce was commonly shared among family and friends, and organic agricultural practices, blending traditional knowledge with modern science, were prevalent. Recycled containers were commonly repurposed for growing, and growers often lacked formal training, learning through trial and error.

In Pacific indigenous communities, food gardens took on a slightly different nature and purpose; 'Teitei' (in Fiji) were the traditional food gardens passed down through generations and allocated through the traditional 'kastom' process. Elders typically reserved fertile land within villages, while younger generations established gardens in forests, steep slopes, and distant sites. Most agricultural labour was manual-intensive, making tasks like soil preparation, harvesting, and transporting produce back to the village physically demanding. Consequently, young farmers tended to manage food gardens across multiple plots, sometimes setting up temporary farmhouses nearby during peak farming periods. Gardeners primarily cultivated crops for household use, exchange, and gifting, with limited commercial sales. These gardens played a vital role in preserving ethnobotanical diversity, serving purposes ranging from food and medicine to spirituality and cultural practices.

#### *Family farm*

The definition of family farming (FF) was broad and heterogeneous. However, most of its problems and opportunities were shared and cut across various regions. Family farming was mostly small-scale (although there was a great variety of land hectares from two or less to more than 10). It was managed and operated by a family and predominantly relied on family labour, including both women's and men's. The family and the farm were linked, co-evolved, and combined economic, environmental, social, and cultural functions. Smallholder family farmers' production strategies typically utilised a mixture of root crops, vegetables, fruits, and livestock varieties for domestic consumption, sale, and gifts. A vast majority of these crops, plants, and trees were either cultivated as mixed crops or followed a scattered type of planting, while a minimum number of the crops were cultivated in monoculture (cash crops). The utilisation of farming inputs was very low. FF aimed for self-sufficiency with part of the produce being used for extended family own consumption and the other part as a source of livelihoods from selling to local markets or family farm stalls. Money generated from the crop sales was used for purchasing daily base needed goods as well as for other cultural and religious obligations. Family farming could also include small-scale aquaculture operations in ponds (450–500 m2).

#### *Community farm*

Landless or community farming referred to farmers who utilised the 'commons' for livestock production or food gardening. Farmers could use the lands independently or be part of a community. Community gardens were very heterogeneous in size, crop mixes, and management levels. Farming took place on idle or undeveloped land in various locations ranging from small towns to large cities. These areas were located on public lands, such as vacant urban lots, unused green spaces on public school grounds, or church land spaces. Such areas included road frontages, empty adjacent allotments, riverbanks and valleys, rights-of-way for proposed or existing paths and roads, and open land in general, including hillsides or swamplands. These farms were organised by charities, non-profit associations, cooperatives, social enterprises, or by groups of neighbours. Cultivation occurred in raised beds or in the pre-existing soil with minimal external inputs, applying principles of organic agriculture. Produce was split among farmers and often sold at the market or through food box schemes to fund garden operations.

### ***Type of foods***

#### *Home garden*

The most common foods produced in home gardens were vegetables and fruits (banana, mango, papaya, citrus fruits), typically part of traditional diets [129, 132, 42,

135, 66, 131, 12, 48, 111, 143, 36, 27, 20, 127, 13, 134]. Planting root crops (such as sweet potato, taro, cassava, maize), tubers, and plantains was also very common [12, 48, 130, 34, 143, 36, 20, 134]. Many households in the Pacific had coconuts and grew kava [129, 48]. Less often, they grew cocoa and coffee [129]. People grew herbs and chili peppers for seasoning alongside vegetables such as melange, zucchini, pumpkin, okra, callaloo (green leafy vegetable), pakchoi, tomatoes, sweet pepper, and cucumber [138, 131, 35, 20, 134]. Most vegetables were consumed fresh, with little or no processing, although hot peppers were often sundried or refrigerated, okra and spinach were blanched, and fruit was processed into drinks [134]. Other foods included chickpeas [140], lemon leaf tea and coconut milk [27] and nuts [20]. Crops that were easily adapted to urban spaces such as Chinese cabbage, pakchoi, and peppers were common in urban gardens [127]. Some kept livestock (mainly chickens) in urban gardens, which also provided them with eggs [42, 135, 131, 66, 131, 41, 19], and there were cases of raising small animals, such as poultry and guinea pigs [111, 136, 133, 19, 130, 13, 129] and cows or goats [19, 130, 13], normally consumed on special occasions. Some home gardening projects promoted apiculture in backyards [111].

### *Family farm*

Family farm involved a wide range of food crops, including local staples and roots [104, 144, 108, 152, 29, 21, 58, 157, 39, 11, 45, 156, 154, 14], traditional fruits (tree crops and vines) [104, 144, 108, 152, 29, 58, 147, 157, 39, 40, 30, 156, 154, 123, 153], vegetables [104, 37, 144, 108, 152, 29, 58, 147, 39, 40, 158, 30, 155, 156, 154, 123, 153], and herbs for seasoning [37]. Family farming had a very old tradition of rice-growing [160, 58, 39]. Additionally, it often included livestock raising [152, 30], such as cows [108, 67], pigs [108, 52, 156, 67, 18, 29], goats [146, 104], sheep [146], pork, and mutton [104], and poultry (including eggs) [108, 146, 104, 30, 156, 122, 67, 18, 29], and milk [149], as well as some cash crops such as coconut, copra, cocoa, coffee, and vanilla [152, 58, 14]. Beekeepers were also part of family farming [40]. Some farmers were involved in seaweed, giant clam, and tilapia farming in pond aquacultures [151, 41, 39, 152].

### *Community farm*

In addition to starchy crops [164, 161, 162, 165, 52], vegetables [66, 164, 165, 162, 35, 161, 52], and fruit cultivation [130, 157, 66, 164, 165, 35, 161, 52], some gardens also cultivated herbs and medicinal plants [104, 164, 162, 35]. Few integrated animal production [104], where poultry and pigs raised in small corrals were a common feature [164, 165, 52]. Occasionally, they included rice [165], beans [161], and coffee [162]. Gardens were also a source of fuelwood, flowers, and nuts [130].

## *Role*

### *Home garden*

The home garden served multiple functions crucial to the economies, nutritional status, and social well-being of the studied populations, contributing to household food security [36, 47], total caloric intake [129, 128, 20], income [132, 133, 134], and savings on food purchases [111, 48, 35, 15]. It supplemented protein-deficient diets [130] and promoted better nutrition [129, 132, 128, 111, 130, 127, 133, 134], although dietary diversity in some households remained low [48]. Homegrown produce offered fresh options, improving household nutrition, livelihoods, and resilience by providing healthy crops for consumption and generating income through surplus sales in local markets [99, 111, 131, 35]. The income generated from home gardening empowered women to work within their communities while caring for their children [131] and led to job creation in produce processing, seedling production, and tree grafting [134]. Such projects also imparted valuable knowledge and skills in vegetable, root crop, and fruit tree cultivation [132], offering financial stability and protection against price fluctuations [35]. Productive gardens instilled pride in growers [99, 142] and satisfaction in contributing to household economies [99], seen as a healthy alternative to market-bought food due to reduced pesticide use [35] and providing autonomy in food choices [35]. Gardening was associated with enjoyment and physical activity [35, 130], while the social benefits included fostering community ties through shared produce distribution and feast provision [130] and preserving cultural values [130, 20, 129]. For rural-to-urban migrants and the unemployed, home gardens offered familiar surroundings and local foods [130], elevating social status through livestock raising [133]. Even in extreme weather, small home gardens provided a vital safety net against poverty, malnutrition, and economic downturns [127, 12], ensuring food security [111]. During COVID, governments in the Pacific and Caribbean promoted home gardening to enhance food security, alleviate stress, and mitigate mental health impacts [59, 48, 138, 139, 140, 141, 42, 135, 66]. For Pacific indigenous communities, food gardens also served medicinal, spiritual, and cultural purposes [34].

### *Family farm*

Family farming played an important role as a source of income and employment for farmers [28, 56, 108, 52, 153, 18, 149, 151], serving as their main source of livelihood [104, 39, 123, 149]. It contributed to family food security [37, 160, 39, 154, 149], dietary diversity [108, 157, 149], calorie intake [15], and healthier diets [147, 155]. Raising animals and fish farming was the primary source of protein for rural and poor families [149, 151]. Livestock also played a socio-cultural role, being kept for special occasions and contributing to community feasts [18, 149]. Some family farmers

carried out diversified agricultural activities and followed agroecological principles that supported environmental sustainability and the conservation of biodiversity [28, 144, 145, 52, 14]. Livestock also provided energy and fertiliser for the soil and had a controlling effect on diseases in farming systems [149]. Family farmers made important contributions to the food and nutrition security of their communities [146, 104, 37, 148], local food supply [104, 52], and reducing dependency on food imports [104, 147]. The production from family farming ensured food access to farmers during periods of supply shortage due to COVID-19 pandemic [114, 37, 108, 150, 40, 159, 124], providing resilience against external shocks, whether economic (price spikes, global recession) or natural (cyclones, floods, droughts, pests, and diseases) [148, 15, 149].

#### *Community farm*

Community gardens played a role in supporting people's livelihoods [104, 52]. The produce of the garden was shared among the gardeners who brought fresh, nutritious produce back to their households [162, 35]. Sometimes, the production was sold in the local market or through food box schemes, contributing to the local food supply [163, 66, 35, 164, 52], including the tourism sector [164]. These gardens revitalised many traditional crops, particularly starchy root crops (viandas) like sweet potato (boniato), potatoes, and cassava, helping to reduce dependency on external food sources [164, 161]. They increased liveability in cities as communal foodscapes reduced the heat island effect and promoted stress-free environments, better mental health, and social well-being [85]. The communal gardens were important educational hubs whose activities taught people about the importance of nature in urban spaces [43]. Some community gardens also acted as cultural centres [162]. In these centres, along with learning new gardening skills such as creating compost to fertilise the garden [162, 161], gardeners learned about the culture, colonial, and environmental history of the island, cultivating an empowered community [162, 75, 161]. Elders taught younger farmers, and knowledge was passed down from one generation to another [162, 75, 161]. The community garden served as a recreational gathering place, playing a socialising role [162, 161]. The garden lots played a role in using previously abandoned spaces often filled with garbage [162, 161, 52], functioning as a form of resilience after climate disasters [162]. Community farmers perceived their pesticide-free food produce as a healthy alternative to purchasing food at the market, also fulfilling their desire to know what they were eating and decide for themselves what they would eat [35].

**Extent**

***Scale of use***

As seen in the UN food security country profiles, food consumed from own production contributeed to the average dietary energy consumption [171, 170, 172, 169, 31, 32]

*Home garden*

Home gardening was reported as a relatively unimportant source of food for urban dwellers. Some of the reasons why urban households did not engage more in home gardening were that they would be victims of theft, that it was easier to buy food than grow it, that they did not have land on which to grow food, that they did not have the time or labour, that they did not have access to relevant inputs and weather worsening conditions such as dry season and hot temperatures [42,135, 35]. On the other hand, literature from Cuba described a very different scenario regarding urban gardening; before 1989, home gardens were rare but during the economic and food crisis urban agriculture was the response to resource constraints. Many city dwellers began to grow vegetables and fruits out of necessity rather than intentionally. Ever since then, urban agriculture has been strongly encouraged by the government, securing access to land and organising urban farmers in cooperatives. This has become a strong urban agriculture movement [128, 66, 111]. Small-scale 'urban' animal husbandry, although playing a minor role compared to food production, is also present but of variable importance. Apart from kitchen waste, the main feed for pigs and chickens in most areas is coconut [130].

*Family farm*

Described as a predominant form of production in SIDS [146], although they were also seen as having economically inefficient small farms, low production and productivity levels, and limited access to new technologies [122].

***Relevant for who?***

*Home garden*

In large part, women were responsible for growing food in home gardens [102, 99, 131]. Some home gardening projects targeted women and students [132]. Although there was no class distinction among participants, who included lawyers, doctors, pilots, accountants, nurses, civil servants, and businessmen, there was a clear gender dimension: home gardening was dominated by women [111]. Home gardening was vital for the subsistence of people living in small, low-lying atoll states [129] and for those living on islands that were predominantly rural, with a high percentage of subsistence agriculture and a lack of off-farm income [36]. It also had particular

importance for some of the poorest rural households [36], and for extended families and urban households who would otherwise have to rely on store-bought foods to meet their nutritional needs [99]. Home gardening was also relevant for families working in rural settlements who came from elsewhere and had no access to extensive areas of rural land [130], and for recent urban immigrants with very low cash incomes who relied on their backyard gardens to save money on food purchases and to pay rents [130]. Residents in neighbourhoods where residential density was low and homes were more likely to have yards with considerable green space were more likely to consume food items cultivated in their yards than residents in densely urbanised neighbourhoods [143].

#### *Family farm*

Family farming was vital for people living in rural areas and highly lagging territories [28, 56, 104, 160, 58, 39, 158, 15, 11, 45, 149] as it was their main source of income and employment. It played a major role in fighting hunger for the poorest families [146, 104, 160, 156, 151]. Women played a central role in family farming [149] although they lagged behind men in terms of access to productive resources such as land, equipment, credit, and technology [133]. The limited population and resources of some small islands restricted their access to raw materials and their ability to benefit from economies of scale, enhancing the importance of family farming in this context [30, 155]. Pond aquaculture was especially relevant for those living in remote, isolated coastal, and atoll communities [151].

#### Community farm

Community gardens were especially relevant in megacities (urban and peri-urban areas) [85, 35, 130, 164, 128, 165, 52].

### Changes over time and between generations

Over time, people moved away from subsistence practices to cash-based economies due to the arrival of logging companies. Logging machines brought and dispersed weeds and snails, impacting home gardens ecologically and affecting production and the time investment people needed for gardening. Logging also changed people's preferences, with fewer showing interest in gardening and becoming used to buying rice from stores whenever they were hungry. This raised concerns among people living near logging companies, as they were aware they depended on them for food [33]. Similarly, the transition to cash-based economies affected home gardens using traditional farming systems. Within villages, cash crops and cattle replaced traditional food gardens, which were displaced to areas further away, making it difficult for people to tend to their gardens. Cash crops were planted in fertile lands, while traditional farming shifted to inferior plots, declining their yields

and increasing their dependence on cash for buying food [34]. A few respondents had noticed a lack of interest in home gardening among youth [35]. Climate change was also mentioned by members of the community as a reason why people were more discouraged to plant, as temperatures increased and issues with water availability limited the capacity to plant more fruits and vegetables [27]. Socio-political and globalisation factors increased the amounts of imported rice by the governments that sold it at a subsidized price to the population. Consequently, the population relied on rice rather than the traditional maize crops from their home garden due to its affordability, availability, and less labour required. Farmer households were forced to buy imported rice at the market, illustrating the importance of rice as a backup food to maize and the vulnerability of poorer farmer households to fluctuations in the market price [36].

Over time, the production of taro and sweet potatoes dropped significantly due to increased access to imported staples, which are more convenient for preparation and storage. For instance, arrowroot, a traditional staple of the atolls, has nearly vanished [29, 21, 18]. Imports now satisfy most of the demand for pork, chicken, and eggs, driven by the cost of animal feed, which must be imported as well [29]. Importantly, food imports have influenced people's preferences, with many opting for fatty imported chicken over locally-raised options or cheaper imported foods like rice, flour, and tinned or frozen meat [21, 156, 18]. These shifts in food consumption, combined with limited and impoverished agricultural lands, pose significant challenges for the government in promoting further development in family farming [21]. Over the years, family farming has experienced a decline, counteracted by substantial growth in tourism and related services [21]. This decline is attributed to factors such as migration to urban areas, foreign investment, and an increased need for cash income, resulting in the sale of much land in villages and reduced available land for family farming [11]. Climate change emerged as the primary driver of crop production variability, discouraging farmers and leaving them feeling vulnerable [28, 29, 21, 30, 31, 26, 18]. Similarly, COVID-related restrictions and sales difficulties had comparable discouraging effects on family farmers [37]. However, in Fiji, the pandemic seemed to have the opposite effect, prompting people to collaborate in gardens and increase food production [45].

## Share

### Nature

*Gift*

Food gifts occurred in various settings, including schools, extended families, neighbours, and communities [41, 49, 10, 47, 21, 42]. Adolescents at school often relied on friends to access less healthy foods [41, 49, 10]. Communities in Pacific Island countries revived traditions of communal support and sharing, such as sharing farming tools, lands, and produce [48, 11]. Fishers in some communities prioritised giving away part of their catch to family, friends, and neighbours instead of selling it, emphasising community relationships and solidarity [74, 71, 40, 166]. Rural communities often shared food, stories, and supported each other, considering themselves as family. For example, when a family member passed away, neighbours came together to support the grieving family. They sent essential food items like rice and sugar to help them during this challenging time [41]. Volunteer-based initiatives and online platforms facilitated food sharing activities, including rescuing surplus food and redistributing it to charities or through potlucks, which were gatherings where each guest brought a dish to share with everyone else [85, 43]. In Singapore, for-profit enterprises were common in food sharing initiatives, with a focus on sharing knowledge, skills, meals, and various food items [167]. Shopkeepers provided gifts of food to community members in need, driven by a sense of moral responsibility and religious beliefs [23]. Food gifting between neighbours and extended family members sometimes involved delayed reciprocity, where the gift was returned at a later time [36]. This type of reciprocity was common in communities where individuals understood that their own well-being was interconnected with that of others, rather than relying solely on unpredictable job markets [c]. Food also served as social capital, reinforcing social responsibilities and kinship ties [54]. Mutual assistance based on love and compassion involved providing food and daily meals to close relations [32].

### *Feast*

Feasting was a common practice observed in ceremonies, festivities, and celebrations [175, 50, 51, 55, 27, 136]. Sundays were often recognised as "the feasting day" [55, 27]. During feasts, the distribution of food was done in a hierarchical and politically oriented manner. Dignitaries from neighbouring villages and tribes received generous portions of food first. Then, other special guests helped themselves from the tables, followed by local community leaders, members of the local community, and finally village members and other non-community guests. Food was typically served in deep bowls or plates, around 20 cm in diameter, filled to the point of overflowing [39, 175]. In Vanuatu, feasting, known as *lafet*, involved sharing food with a broader group of individuals beyond the immediate family. *Lafet* was commonly associated with weddings or funerals, lasting between three and ten days, with most lasting five days. Elders within the host family determined the duration based on available financial and food resources. As people gathered in

advance, they constructed temporary dwellings made of bamboo with roofs made of corrugated tin or traditional thatched roofs. These dwellings provided shelter from rain and sun during cooking and eating, serving as a gathering point. Activities such as creating large earth ovens with stones for cooking, building temporary pigpens for gifted pigs, and organising the event beforehand extended the feast's total duration to accommodate the needs of attendees, those involved in preparations, and their families. Other community celebrations involving feasts included church events, public holidays, visits by missionaries, Mother's Day, Father's Day, and birthdays. These events often adopted a potluck approach, where individual families contributed various dishes to the communal meal. Examples included stews with beef, chicken, or fish served over rice, cakes for dessert, platters of local fruits, or traditional *laplap* pudding cooked in earth ovens. All the food was combined and presented on a communal table. Unlike weddings and funerals, these feasts typically lasted for one day and revolved around a single large meal. Social obligations related to customary practices ensured that feasts were communal events that did not overly burden the host family. All attendees were required to bring a gift on behalf of their family group, including items like yams, taro, coconuts, bananas, fresh beef, and pigs. In urban areas, 25-kilogram bags of rice and 12-kilogram flats of sugar had become popular gift choices. A traditional ceremony, where each gift was presented and acknowledged to the host family, allowed guest families to attend the feast and eat on subsequent days. These food gifts enabled the host family, with the support of extended kin networks, to prepare and serve large quantities of food to all attendees [50]. All community members actively participated in meal preparations. Tasks were organised in advance, with individuals assigned one or more tasks based on the community's needs. Food production activities were segregated by gender, with women making bread, preparing taro paste (*popoi*), and cooking, while men fished and provided beef and goat meat. Community meals, where festive attire was worn, were preceded by speeches and prayers [32].

#### *Barter*

Food barter referred to the practice of trading food for other goods or services without involving money [136, 48]. This exchange occurred between coastal and mountain communities [136], as well as among families in the same area, where different types of food, like fish and cassava, were exchanged [48]. It also involved swapping traditional food crops, such as local atoll food, for imported and processed food between relatives living in outer islands and urban centres [29]. During COVID-19 pandemic, the availability of disposable income decreased, leading to a resurgence of this cultural practice in the Pacific region [173,44,174, 48]. Social media platforms, like Facebook group pages, emerged as popular platforms for facilitating such exchanges, with traders sharing photos and arranging pickups without the

involvement of money [173,44,174, 48]. These platforms were monitored by family members who ensured compliance with group rules, such as no money exchanges or illegal trading [173,44,174, 48]. Food barter was not limited to social media platforms. One example was bartering produce from a small fruit and vegetable garden with neighbours in exchange for their labour. For instance, a person with a garden might have offered their fresh produce to a mechanic who, in turn, helped fix their car [166]. This practice demonstrated how people could use their resources and skills to meet their needs through direct exchanges within their local community [33, 166]. Food barter served as a valuable strategy, especially in uncertain times like climate disasters or economic downturns [173]. For instance, when facing unemployment, individuals might have relied on others to acquire essential food and supplies. In return, they repaid their debts by offering their assistance in household chores like cooking, cleaning, and appliance repairs. Additionally, they contributed to small projects at their church, receiving modest compensation in cash or food [23, 53]. These examples highlighted the importance of trust and social networks in meeting household needs [23, 53].

*Remit*

Food remittances typically involved the exchange of food between different locations, such as from a main island to outer islands within the same country or between relatives living in different countries or across diverse community settings like coastal and highland areas. This exchange often occurred within familial relationships, distinguishing it from barter which may have lacked this personal connection. Food remittances from overseas occurred once or twice a year and were more common during the festive Christmas season when people often engaged in excessive spending on food, leading to increased food access [42]. Rural-urban food transfers also occurred between family members and relatives [42, 135]. Improved mobile connectivity allowed people in rural communities to contact family members or contacts in urban centres to purchase fresh fruits and vegetables that could be transported via weekly boat [27]. For people living in the capital city in Tuvalu, for example, most of the local food came from the outer islands and was distributed among extended family through an informal trading system. Family members sent store-bought goods to those in the outer islands in exchange for local foods [18]. Urban dwellers ensured food security by relying on provisions and meats provided by family and friends in rural communities [139]. During disasters like Cyclone Pam, families sent produce from one region to another to supplement diets and maintain kinship networks [12]. In some cases, unprocessed food products were sent from remote islands and then shared among siblings and friends. Receiving such a gift from one's original island held great emotional significance, surpassing its economic

value. These foods were believed to possess a unique taste and flavour that distinguished them from the same foods bought in stores [32].

### *Type of foods*

#### *Gift*

Food gifts involved sharing various types of foods. Among adolescents, snacks, pancakes, cakes, and soft sugary drinks were commonly shared [41, 49]. After a fishing trip, the catch, including finfish, shellfish, and seafood, was shared among the participants [46, 38, 74, 71, 172]. Similarly, produce from the farm (including cereals, tubers, plantains, greens, and chickens) and canned fish were distributed within the community [48, 169, 168, 43]. Chicken quarters were shared among wealthier households [168]. During the COVID-19 pandemic, neighbours exchanged a variety of foods such as coconuts, mangoes, cucumber, golden apples, sour orange, plantain, green bananas, and limes [40]. In times of mourning, food staples like rice and sugar were shared between neighbours to support grieving families [41]. In food sharing institutions, sharing meals included various food items, such as fruit, vegetables, meat, fish, and non-prepared food products [167, 85]. When providing food to those in need, milk and eggs were included [23]. In rural communities, food gifting focused on cassava, maize, hulled rice, and leafy greens [36, 10]. Within villages, sharing of food was commonplace, with individuals exchanging items like fruits [11].

#### *Feast*

Feasting involved collecting large quantities of food. Participants associated feasting with fresh meat (poultry, mutton, pork, or beef), island-grown vegetables such as yams, taro, and plantains cooked in an earth oven, and an ample serving of food [175, 27, 136, 50]. Fish, ice cream, canned fruit salad, sodas, and sweetened beverages were also commonly reported during feasts [55, 51].

#### *Barter*

Food bartering involved trading store-bought food items such as rice, noodles, and canned goods [33]. It also included the exchange of plantains, homemade scones, pies, fruits, vegetable garden produce, fresh fish, shellfish (lobster), vegetable seedlings, groceries, brownies, and piglets [173, 44, 174, 166]. Wild foods were traded for items like rice, flour, sugar, and canned fish [75].

#### *Remit*

Food remittances involved roots, greens, fish, shellfish, and some wild game meat [136]. There was also a practice of exchanging local atoll food for imported food

between relatives living in the outer islands and those residing in urban centres [29]. Provisions, meats, fresh fruit, vegetables, fish, pulaka, coconuts, and breadfruit were exchanged between rural and urban communities [139, 42, 135, 11, 18, 27].

*Role*

Food sharing played a significant role in various aspects of people's lives. It was rooted in traditions and cultures, promoting communal support, and strengthening a spirit of working together and looking after one another [46, 39, 41, 53, 54, 43, 38]. One of the key functions of food sharing was to enhance food security [42, 40, 13, 47, 85, 43, 36, 135, 42]. Especially during the COVID-19 pandemic, food sharing brought communities together, emphasising self-reliance and the sharing of locally produced food [45, 48, 40]. Food sharing could have played a role in dietary diversity and intake, positively impacting health status [49, 168, 171, 170, 172, 169, 43, 52, 117, 18, 10, 47, 13]. It also addressed issues related to food waste and promoted education [85, 43]. Food sharing acted as a safety net, providing support to those in need and creating a sense of moral responsibility towards food-insecure communities [11, 23, 85]. In some cases, the decision to share or not share food became a sign of judgment. If someone chose not to share food, they might have faced stigmatisation and criticism. As a result, people might have felt obligated to share food to avoid negative consequences or judgment from others [53]. Food sharing could also be used as a strategic move to gain sympathy and assert one's position in complicated family land ownership situations [32]. The role of food sharing was economic with regard to subsistence and security, social with regard to cohesion, and ecological with regard to sustainability [46].

In particular, feasts held a significant role in cementing bonds between communities and honouring leaders. They provided an opportunity for nourishment and served social and ritual purposes [175]. Feasts also served as a means for children from poorer families to access food [50]. In remote communities, food sharing increased access to food imports, while in urban centres, it helped enhance access to local foods [29]. Food sharing played a crucial role in mitigating the effects of disasters on food security [139, 12]. Feasts might have supplied individuals with up to three times their daily caloric requirements. Women reported consuming higher amounts of total energy on feast days compared to non-feast days [51].

**Extent**

*Scale of use*

Food sharing was prevalent among adolescents, particularly in school settings [41]. Community-based findings revealed that approximately one third of participants engaged in food sharing with neighbours and other households, with a weekly

frequency reported by 30% of individuals [42]. Once study highlighted a concerning trend towards individualism in rural areas, posing a threat to food sharing practices [46], while other recent studies noted a resurgence of food sharing activities following the COVID-19 pandemic [48, 173, 174].

Food gifts constituted 41% of overall sharing activities in Singapore [167]. In regions such as Kiribati, Tonga, Tuvalu, and the Solomon Islands, the consumption of free food received and consumed at home contributed between 6% and 10% to the average dietary intake [171, 170, 172, 169]. A volunteer-based food sharing initiative in Singapore rescued and distributed a substantial quantity of edible food products, amounting to 2 tonnes on a weekly basis [85]. Urban and peri-urban gardeners actively engaged in food gifting, with 78% of them contributing food to the community [52]. Additional sources of food included communal meals with family and friends (60%), food received as gifts from loved ones (16.1%), and contributions from local churches (2.5%) [13].

Traditional feasting occasions, occurring on a weekly basis, expanded in recent years, particularly in urban areas, encompassing village fiestas, holidays, birthdays, graduations, weddings, rosaries, and funerals [27, 51]. Rural-urban food transfers played a pivotal role, with 25% of households receiving such transfers on a weekly (8%) or monthly (46%) basis [135]. Additionally, 21% of households received food remittances from abroad, typically occurring once or twice per year [42]. The outer-inner islands remittances emerged as the primary source of local foods, contributing to household food supply [18]. Importantly, food sharing was relied upon by a substantial proportion of households, ranging from 23% in Fiji to 40% in Saint Vincent and the Grenadines, to acquire necessary food items [10]. The communal significance of food was demonstrated by dedicating a substantial portion (45-82%) of locally grown food in Tonga and the Solomon Islands to be shared among family members and the broader community [54].

***Relevant for who?***

In rural areas, food sharing followed customary practices and played a vital role in community dynamics, especially during challenging times [39, 131, 50, 32, 30, 38, 74, 41, 45, 11, 36, 166, 75, 10]. Adolescent boys, for instance, might have felt hesitant to bring food from home to school but were more comfortable sharing food from their female friends [41, 49]. Urban dwellers engaged in food sharing with neighbours and other households in their communities [42], while also receiving food sent by relatives from rural areas, particularly in response to climate disasters [42, 135, 12]. Food sharing was especially relevant for food-insecure communities, as rescued food was redistributed to support their needs [85]. Low and very low food secure families used food sharing with extended families as a coping strategy to enhance food

availability [47]. Wealthier households tended to receive more food for free compared to poorer households, potentially influenced by larger social networks and greater involvement in church activities [168]. Older individuals particularly benefited from food sharing, especially when physical limitations prevented them from accessing gardens or forests, relying on accessible food or sharing among family members [11, 54]. For unemployed individuals, food sharing might have become a last resort, either through requesting food gifts [23] or exchanging labour for food (bartering) [23]. Gardeners often donated food to schools, nurseries, nursing homes, and neighbours, particularly those in less fortunate circumstances [52]. In the case of food-insecure households, 14% of their dietary energy came from fish and shellfish received as gifts, predominantly in the form of canned fish [168]. Feastings also benefited food-insecure children in urban and peri-urban areas, ensuring they were fed, even if their parents had not contributed a gift to the event [50]. Food sharing, whether in the form of bartering or remittances, was especially relevant for remote rural communities with limited access to food stores [33, 27]. For vulnerable groups and those below the poverty line, access to formalised bartering systems could have served as a temporary buffer, dependent on the resources available for exchange [44]. Additionally, individuals with higher education levels and those living in smaller households were more likely to regularly consume shared food [10].

**Changes over time and between generations**

The adoption of Western practices has led to an increase in feasts, especially in urban areas, where public holidays and religious ceremonies were celebrated more frequently. Peri-urban villages experienced population growth due to migration from the outer islands, resulting in more weddings, funerals, and celebrations [50].

As rural economies became more monetised, there was a shift away from subsistence fishing for home consumption or meeting social obligations, with more fish being traded for cash [38]. This shift towards individualism in food sharing practices posed a threat to the social aspect of marine resource use. Factors such as easier access to fishing grounds and markets, changing preferences for specific fishing techniques, and the need to earn money contributed to this trend. Improved transportation and storage facilities granted fishermen greater independence and access to resources. Foreign aid projects aimed to enhance market structures, reducing dependence on communal support. These changes had the potential to alter people's preferences, shifting from collective approaches to more individual ones [46].

In urban areas, sharing or not sharing food within households became a way to judge relationships. There was a new household dynamic where membership was based on who could contribute. Not sharing food was stigmatised, and people often

shared out of fear of negative consequences. Instead of collective sharing, the focus was on individual contribution, creating social distance. This change affected family bonds, especially with unemployed young men who were excluded from sharing. Urbanisation and economic factors played a role in this transformation. To cope, young men formed supportive relationships outside their families, like informal groups or "squads." These networks helped them navigate an unsupportive living environment [53].

Changes in social and familial structures, larger communities, and access to store-bought food made traditional safety nets less important. Food played a vital role in cultural celebrations and bringing people together. However, changing lifestyles and extreme weather events disrupted these traditions. For instance, the availability of certain foods used in celebrations was affected by climate extremes. The destruction of yams, an important crop, after Cyclone Pam made replanting costly and challenging. Family networks struggled to provide the necessary resources. The threat of future climate extremes discouraged investment in traditional farming practices. These changes impacted the social fabric and cultural practices of the communities [11].

Most of small islands' cultures were communal, and the sharing of work and food were once central. Traditional food-sharing practices served as a type of social safety net, the means through which food was produced and distributed to ensure community well-being under challenging ecological conditions. Yet today, in less traditional urban areas, it was implicitly acknowledged that there were some ways in which food sharing beyond one's immediate nuclear family may threaten its food security. For example, the obligation to provide food for community and church feasts could be a notable challenge for many families. Some respondents noted that the only time they bought healthy, high-quality foods was when they were preparing a meal for such a feast. Likewise, the cultural obligation for individuals to host and provide for extended family members was upheld even when it came at the expense of their household's food security [18].

## State

### Nature

This type of food source was provided by the government and included school meals, food baskets, and food assistance.

#### *School meals*

School meals came in various forms. One type of programme covered all government and government-assisted schools but excluded private schools, offering daily free meals to students. Primary school teachers used Ministry of Education guidelines to select eligible children based on factors such as parental employment, family size, and specific medical conditions [69]. Some programmes consisted in preparing traditional cooked meals in school canteens using supplies like flour, rice, and oil provided to schools and grants to buy additional items, while other programmes prepared the lunches centrally and distributed to schools [70, 47]. Funding for meals in nursery and primary schools was provided based on the number of enrolled students, without specific conditions, but schools were required to have food preparation facilities, management committees, bank accounts, and undergo training. Applications were reviewed by the Ministry of Education, and funds were transferred via bank transfers [63]. In the Caribbean region, school meals programs had a common characteristic of being universal. This meant that all students enrolled in public schools, regardless of their socio-economic status or where they lived, received free meals. Universal programs were more likely to include non-poor children and had objectives beyond income considerations. In the Caribbean, families with higher incomes often chose private or semi-private education, so the school meals programs provided to public schools already served the less affluent sections of the population. Providing a daily meal that met national guidelines remained a challenge, mainly due to funding limitations. This issue was reported in countries like Haiti, where meals might not have been consistently provided throughout the year, or the food rations might not have always met national standards [64]. Some programs integrated school gardens, cultivating diverse plant species to supply fresh vegetables for school lunches and for the wider community. These schools had dedicated kitchens and predominantly served lunchtime meals [68, 67]. During the COVID-19 pandemic, school feeding programs had to adapt. They introduced measures such as providing take-home rations, home deliveries, food vouchers, and temporary cash support [61]. Efforts were also made to enhance the nutrition content of school meals by strengthening market linkages with farmer organisations. Similar initiatives were implemented in government institutions like prisons and hospitals [60].

*Baskets*

Food baskets were another type of food source, offered by the government and typically containing locally grown foods, thus supporting local farmers [58]. Certain programmes used beneficiary cards, which needed renewal every two years. These initiatives offered discounted weekly food packages, such as rice and flour. Beneficiaries purchased the basket but received discounts at specific stores when using their card [62]. Many of these programmes worked on diversifying food

baskets by adding fresh products and traditional foods that reflected local culture [64]. Some programmes used ration cards, allowing people to buy a fixed amount of food at a set price [66]. In some cases, certain food items were exempt from taxes, and food vouchers were provided to help struggling households [32]. However, some programs faced challenges in providing consistent support, with only small monthly food baskets given to families in need [47].

#### *Assistance*

In certain cases, national governments provided food assistance in response to specific events. To address the limited market access for farmers, some governments established connections between farmers and schools, allowing farmer groups to supply the feeding programs [58, 60, 64, 65]. These programmes were reported as having improved healthy school menus for children and increased food security through increasing knowledge and introducing new and improved farming technologies for the local market [65].

### ***Type of foods***

#### *School meals*

Foods provided by school meals varied and could include breakfast, snack, lunch, or a combination of these [56]. School gardens contributed to these programs by cultivating crops such as cabbage, beans, cassava, tomatoes, tropical seasonal citrus fruits, coriander, bele, and eggplant. Some schools engaged in additional food production activities like poultry rearing, hydroponics, beekeeping, fish farming, pig farming, and fruit tree cultivation [67]. In the case of centrally prepared lunches distributed to schools, the meals typically consisted of a bun and milk or a sweet drink [70]. Biscuits and locally made juices were also included in some instances [63]. Free school meals included ready-to-consume snacks that were typically served as breakfast or mid-afternoon snacks. These snacks were composed of a beverage such as milk, milk-based or cereal-based drinks, juices (fortified or non-fortified), along with dry food products like bread, cookies, granola, and occasionally fresh fruit [64]. Other food items found in these meals included local fruits such as watermelons, bananas, tangerines, and oranges, fish, imported vegetables like carrots, onions, and Irish potatoes, and local vegetables such as tomatoes, cucumbers, string beans, sweet potatoes, and cabbage [65]. However, the introduction of fresh products posed challenges in ensuring food safety, leading many programs to rely primarily on non-perishable items [64].

#### *Baskets*

In the food baskets, the subsidised food typically included rice and flour [62]. Some programs also incorporated animal products, fresh fruits and vegetables, milk, or fortified milk-based beverages [64]. In some instances, certain food items like milk were designated specifically for children under 7 years, elderly individuals, and pregnant women due to shortages. *Bodegas*, local convenience stores, were often the primary source for obtaining basic food products such as rice, sugar, cooking oil, and coffee [66]. Different food baskets based on vulnerability criteria were increasingly being implemented, where children attending schools in particularly vulnerable areas were entitled to more or different types of food support [64].

### *Role*

#### *School meals*

School meals played a role in various strategic areas, including education, health, agriculture, and territorial development. These programmes aimed to address poverty and promote the well-being of children and adolescents, often being linked to local food production, such as purchasing from family farming, thereby contributing to a more inclusive development of the local economy [56]. The main goals of most school feeding programs were education, with a growing recognition of the importance of food security and nutrition in achieving educational outcomes [64]. School garden projects were developed as sustainable and replicable models to promote community and environmental livelihood. These projects involved the creation of organic school gardens and had the potential to positively impact the community and environment [68]. Impact evaluations showed that school meals increased enrolment, improved classroom behaviour, and acted as a safety net during periods of price shocks [63]. Successful outreach efforts included training teachers, parents, and school canteen workers [57]. Governments increasingly sought to provide nutritious and healthy school meals and snacks that were culturally adapted, as well as to connect them to local food production and local economies, which could create long-term market opportunities for local farmers [64, 58, 60, 65]. This collaboration between farmers and schools not only promoted the cultivation and preparation of nutritious food in school meals but also exposed students to healthy foods from an early age by incorporating them into school menus. By building integrated value chains that connected small producers and consumers with markets, countries could reduce their reliance on food imports and contribute to the sustainable growth of their economy [58, 60, 65].

#### *Baskets*

Food baskets played a role in mitigating the COVID-19 impact of restrictions on farmers' access to local markets. Farmers expressed enthusiastic feedback and emphasised the need for its continuity [58, 59].

**Extent**

*Scale of use*

School feeding programmes were widely implemented in the Caribbean region, with a significant number of countries adopting these initiatives [56, 64]. The provision of free school meals was prevalent, although the extent of coverage varied among nations [69]. Additionally, a substantial proportion of schools incorporated organic gardens, highlighting the significance of school gardens in supporting these food programs [67].

*Relevant for who?*

There there was recognition of the importance school feeding programmes in targeting vulnerable children and their families [64, 69, 70, 47, 64]. Research suggested that the receipt of free school HYPERLINK "https://embed.kumu.io/efc4aa64e21adec36bb268e0059c6813"r family, lower educational attainment of parents, long-term unemployment of the father, and fewer household amenities [69]. Regarding food basket programmes, its objective was to provide subsidised food to working poor families [62] and the most disadvantaged households [32, 47].

**Changes over time and between generations**

Over time, school feeding programmes underwent significant transformations. Initially, they primarily focused on providing food to hungry and impoverished students, encouraging regular school attendance. However, these programmes were later recognised as integral components of the country's safety net system, ensuring universal access to both food and education. Despite their importance, not all countries established clear laws or policies governing these programmes, leading to coordination challenges with other support initiatives [64]. The influx of forced migrants presented additional challenges in host communities. A comprehensive assessment of these programmes revealed inadequate staffing levels and programme capacity in these areas, underscoring the urgent need to increase resources to meet the growing demand and adequately support newly enrolled students [63].

## Wild

### Nature

#### *Forage*

Wild areas like forests and bushlands served as valuable sources of food through foraging practices. Additionally, old farm areas that had been left unused also yielded food crops that naturally regrew without the use of chemicals [75]. Collectors of wild foods were often specialised, focusing on a specific type of food, while others targeted various food types. Specialised collectors were typically older individuals, whereas those collecting multiple food types and non-collectors tended to be younger [75]. Consumption of wild foods varied throughout the year, with lower consumption during the wet season and a greater diversity of wild foods available during the dry season. Tubers like sweet potato, cassava, and wild yam were dried for storage and consumption during the wet season. Various parts of wild foods, including tubers, seeds, flowers, fruits, and leaves, were consumed [176].

#### *Hunt*

Hunting typically involved individuals or small groups working together to hunt animals using trained dogs, spears, or bows and arrows [45, 33].

#### *Fish*

Marine environments, including the open ocean, mangroves, mudflats, coral reefs, and rivers, served as vital sources of wild food. Coastal communities harvested protein from both the ocean and land, while inland communities relied on freshwater habitats and surrounding areas [48]. Artisanal fishing methods, such as manual collection, spearfishing, and hook and line fishing from traditional dugout canoes, were commonly employed [33]. Shore-based fishing activities, like gleaning and swimming, were prevalent, often using non-powered canoes [38, 71]. Subsistence fishing typically involved small boats with outboard motors owned by individuals [180]. Fishing knowledge was transmitted across generations, with community participation being integral, and catches shared within the community [38, 71]. Gender dynamics played a significant role in fishing practices, with fishing activities often segregated based on location or habitat. Women were predominantly engaged in mangrove and nearshore fishing, while men focused on offshore and reef fishing [76]. Women and children tended to participate in shallow-water fishing, while men undertook more physically demanding tasks, such as deep-sea fishing and diving [33]. Spearfishing near the shore became prominent, especially with the introduction of diving goggles, targeting easily speared fish primarily for family consumption or occasional sale [181]. Women were involved in reef gleaning,

collecting invertebrates, and preparing food from the catch, using simple gear like handlines and hand nets [76, 14]. Different species were targeted for consumption and sale, with women concentrating on specific species based on habitat, like giant clams and spider shells in coral reefs [76]. Various transportation methods, including walking, bicycles, motorcycles, cars, and motorised boats, were used to reach fishing sites, with women fishing closer to their villages, typically for shorter durations, often during low tide hours and mornings [76, 73]. Traditionally, fishing communities prioritised meeting immediate needs over financial gain, with any surplus being shared within the community [74]. Fishing held high regard as a profession in the Pacific Islands, deeply influenced by community values [74].

### *Type of foods*

#### *Forage*

Wild food sourcing involved a diverse range of food types. Foraging as a food source included tree crops like coconut, banana, and breadfruit, as well as wild native plants such as ota (edible ferns), mushrooms, wild dark green leafy greens, fruit trees like papaya, maripa, and hog plum, nut trees like chestnuts, staple foods like sago, bitter beans, and root vegetables such as taro, cassava, and various types of yam.

#### *Hunt*

In hunting, the most reported animal was the wild pig. However, hunters also targeted a variety of mammals, rodents, reptiles, birds, insects, and worms, and collected animal products such as honey, eggs, and bird's nests.

#### *Fish*

Regarding wild fishing, the studies reviewed mentioned various fish species, shellfish, molluscs, crustaceans, and seaweeds, highlighting the extensive range of resources obtained from wild fishing practices. The findings indicated that more than 160 different fish species and over 100 species of invertebrates and seaweeds were reported as significant sources of wild food.

### *Role*

The evidence reviewed suggested that wild food sources served various functions at the individual, household, and community levels. Primarily, they provided food for personal or family consumption, contributing directly to food security, particularly during lean seasons, and acting as a buffer against external shocks, such as those caused by events like the COVID-19 pandemic. Additionally, wild food sources played a vital role in enhancing dietary quality and diversity, offering essential nutritional supplements and valued animal-source proteins. They also had the

potential to improve livelihoods by reducing the need for cash spent on food purchases and serving as sources of natural medicines and construction materials. Wild food sources could generate cash income through surplus sales, or the commercialisation of artisanal products and crafts derived from them. They also held cultural significance, often being used in ceremonies and rituals, and were linked to the spiritual importance of forests as ancestral spaces. Traditional knowledge was transferred through hunting and fishing practices, with elders teaching young individuals while sharing stories, traditions, and biodiversity knowledge. Wild food sources contributed to personal well-being, providing a sense of nutritional value and perceived health benefits, while also offering enjoyment through lifestyles. Moreover, wild food sourcing had environmental benefits, regenerating abandoned land without agrochemical use. Despite often being overlooked, the social and economic impacts of wild food sources were significant, contributing to local economies through surplus sales and enhancing community values and social ties through shared catches. Fishing ability could symbolise social status, with individuals gaining prestige by catching more fish and enhancing their reputation within the community by sharing greater amounts of the catch.

**Extent**

***Scale of use***

The evidence reviewed highlighted the cultural and economic significance of wild food sources, underlining their crucial role in ensuring food security and livelihoods [133, 151, 177, 148]. They were described as a primary and essential source of animal protein [178, 33], and in some instances, they served as the primary source of income [16]. In the context of Pacific islands, the dependence on subsistence fisheries was not solely attributed to a lack of development but rather recognised as a state of "subsistence affluence" [43]. This term reflects the notion that despite not being measured in monetary value, reliance on wild food sources allows for a good quality of life.

***Relevant for who?***

Wild food sources had a significant impact on rural populations, particularly those living in remote areas. While urban populations obtained little energy from wild foods, rural communities relied on them for subsistence. In rural settings, people commonly sourced meat from the bush, whereas urban areas relied on less healthy options like processed sausages or fried chicken [136]. In small low-lying atoll communities, wild food sources were particularly important. Almost every family in these communities had someone actively involved in fishing, highlighting the role it played in their livelihoods [74, 72]. Moreover, in countries such as Tuvalu, the

Solomon Islands, Vanuatu, Papua New Guinea, and Niue, the subsistence sector accounted for over 80 percent of the coastal catch, showing the reliance on wild food sources [133, 38, 71]. Wild foods not only contributed significantly to the food basket of cash-poor rural households but also served as a valuable source of income [133, 163, 142].

**Changes over time and between generations**

Over time, wild food sources remained important in rural areas. However, as rural economies became more monetised, there was a shift towards trading wild foods for cash. This led to the involvement of intermediaries and changes in how wild foods were distributed, reaching towns and nearby islands [72]. This shift also brought competition for resources and overexploitation [71, 46, 18, 73]. The importance of being near wild food sources changed in rural communities, reflecting the value of people's time. As rural areas became more modern and focused on money, the significance of being close to wild foods was not just about getting food, but also about showing social status. This shift showed how rural communities changed and how people's priorities were influenced by time and money [11]. Urban migration impacted fishing in communities, requiring negotiations for fishing rights with newcomers [74]. The availability of imported food in urban contexts as substitutes for traditional foods led to a decline in the collection and consumption of wild foods. This shift resulted in a social stigma associated with collecting wild foods and linked superior socioeconomic status to the ability to purchase imported food items [75]. While there were concerns about the decline in the collection and consumption of wild foods [26], examples were found of adolescents and children who demonstrated a strong interest in preserving traditional practices such as fishing, collecting wild foods, and maintaining a village lifestyle [20]. HYPERLINK "https://embed.kumu.io/15dce7cfe928c28570ef1e873294024f"range of habitats and a greater diversity of fish, invertebrates, and seaweed [76]. Additionally, the COVID-19 pandemic prompted households to rely more on nearby rivers, the sea, and wild forest harvests for their food supply [48].

## Interconnections between food sources

Here we report all the interconnections between food sources we identified from the evidence reviewed, with the corresponding references in brackets. A visual representation of these interconnections can be found in the interactive open-access evidence map that we developed for this paper.

**Buy (From) - Remit (To)**

Family members in Funafuti (main island in Tuvalu) send store-bought goods (e.g., food and other items such as Chinese floor coverings) to those in the outer islands in exchange for local foods. [18]

**Grow (From) - Marketplace (To)**

People use their gardens as a source of income by selling produce at local markets and shops. Own production has also created jobs in the processing of produce into sauces, jams and jellies, the production of seedlings, and grafting trees. [111]

**Home garden (From) - Feast (To)**

Home gardeners raise plants of tremendous ethnobotanical importance ranging from those for food to medicinal, spiritual, and cultural purposes such as rituals and ceremonies, community gatherings, treating ailments, and dyes for painting. The social benefits of home gardening are manifold and must be seen as significantly contributing to the quality of life and the protection of cultural values, as, for example, the maintenance of social ties through the distribution of garden produce and provision of food for feasts. [130, 131]

**Fish (From) - Informal grocery (To)**

Most rural-based women fishers use their catches primarily to ensure household food security. Since no cash is involved, these fisheries are viewed by policymakers and donors as less important than commercial fisheries. However, women are becoming increasingly involved in commercial fisheries, including for trochus, as well as in adding value to their catches. Many women with access to markets collect fish, octopus, and shellfish, including giant clams for the preparation of traditional puddings covered in coconut cream to produce a value-added product for sale to a range of buyers along the roadside. [71]

**Buy (From) - Barter (To)**

Food bartering involves trading store-bought food items such as rice, noodles, and canned goods in exchange for local foods. [33]

**Community farm (From) - School meals (To)**

In school feeding programs where a combination of local and imported food is distributed, communities are pleased with the opportunity to sell part of their local harvest, benefiting their children and generating additional income. This is especially valuable in rural areas where limited access to markets poses significant challenges for farmers. [101]

**Home garden (From) - Gift (To)**

Surplus production from home gardens is often shared among family and friends. Home gardeners predominantly cultivate crops for household consumption, exchange, and gifting and do not generally sell their produce. Home food gardening was a way to connect with their families and their community, a way to share with family members and neighbours. The maintenance of social ties through the distribution of garden produce and provision of food for feasts. Sharing extended beyond food and included sharing knowledge. Several participants indicated they had learned about gardening from other family members, such as parents or siblings. [35, 130, 134]

**Community (From) - Assistance (To)**

Throughout the COVID-19 pandemic, NGOs and indigenous communities have collaborated with the government to mitigate the impacts of control measures on farmers and households. For instance, organisations such as FRIEND in Fiji, MORDI TT in Tonga, and KGA in the Solomon Islands are actively involved in projects aimed at distributing seedlings, medication, and agricultural equipment to support local communities. [48]

**Aid (From) - State (To)**

Food aid serves multiple functions in supporting communities. Primarily, it ensures the provision of essential local public services. For example, many Pacific governments depend on assistance from foreign donors to sustain the delivery of fundamental public services. Additionally, food aid extends support to government safety net programs, such as the national school feeding program. [102]

**Informal grocery (From) - Feast (To)**

Kilograms of rice and sugar were purchased from a family-owned store in town for feasting purposes. [50]

**Target (From) - School meals (To)**

Most Latin American and Caribbean (LAC) countries initially started programs dependent on external funding and support for implementation, primarily from the World Food Programme (WFP). Over time, these programs transitioned into national initiatives funded and overseen by respective governments. The shift to national ownership varied across countries, yet it typically involved a gradual process of institutionalisation, marked by the development of national policies, norms, financial structures, and management capacities. [64, 103]

**Home garden (From) - Informal grocery (To)**

Women that were also street-food vendors knew there was a market for chicken selling so they were interested in raising chickens at home than growing vegetables. Chickens were sold live or slaughtered, either fresh (chilled) or frozen. [131]

**Wild (From) - Feast (To)**

There are broader cultural links between wild food sources, such as their use in ceremonies and rituals. [163]

**Grow (From) - Gift (To)**

Produce from the farm and urban and peri-urban gardens (including cereals, tubers, plantains, greens, and chickens) and canned fish, is distributed within the community. [54, 52, 48, 169, 168, 43]

**Wild (From) - Buy (To)**

The evidence supports that wild sources of food can generate cash income, contributing to local economies when the surplus is sold commercially or when artisanal products and crafts derived from them are sold. [177, 163, 75, 133, 71, 72, 33, 124, 142, 76, 108, 46, 14, 74]

**Home garden (From) - Marketplace (To)**

Home gardens surplus was sold at the local market for a small income. There was connectivity between marketplaces and home gardens. The most frequently purchased perishable crops from the market were sweet potato, cabbage, tomato, and cucumber. The purchasing of higher-value vegetables (i.e., tomato, cucumber, and to a lesser extent capsicum, eggplant, snake bean, and spring onion) is likely to reflect the fact that these crops can be more difficult to produce within a subsistence-based home-garden due to the accessible of planting material, and the need for more intensive production practices. The purchase of traditional crops from the market, particularly sweet potato, cassava, and plantain, all of which are commonly grown within subsistence-based home gardens, is more difficult to explain. It is possible that participants visit the markets to purchase items that they do not have ready access to in their garden, or in the case of traditional crops insufficient quantity to support a specific need (i.e., customary or family event). More speculative, is the possibility that the occasional purchase of traditional crops from the market, ensures home gardens are not fully depleted maintaining a reserve source of household food. [123]

**Family farm (From) - Gift (To)**

The primary goal of the family farm is self-sufficiency, with a portion of the produce allocated for the extended family's consumption. Every crop planted in the gardens serves the purpose of supporting the family's daily meals, and they also share their harvest with five neighbouring families. [144, 108, 18]

**Wild (From) - Barter (To)**

Wild foods are traded for items like rice, flour, sugar, and canned fish. [75]

**Home garden (From) - Community farm (To)**

Home growers tried growing boxes in their backyards but eventually shifted to a community garden approach because of the time requirement. [35]

**Community farm (From) - Gift (To)**

Communities grow food as a hobby, for pleasure and well-being, and to share with neighbours, especially those who are not very well-off. [162, 35, 52, 54]

**Grow (From) - Feast (To)**

Food was grown to meet church and other community obligations (such as weddings or funerals). [54]

**Home garden (From) - Barter (To)**

Produce from a small fruit and vegetable garden is bartered with neighbours in exchange for their labour. For instance, a person with a garden may offer their fresh produce to a mechanic who, in turn, helps fix their car. [166]

**Forage (From) - Grow (To)**

Gathering and own production are mutually reinforcing activities. Replanting after harvesting wild foods is an established rule among farmers, and it is not uncommon for farmers to take advantage of the rich soils of the forest to cultivate other crops. [75]

**Fish (From) - School meals (To)**

Fishing surplus was designated for local primary and/or secondary schools. [76]

**Fish (From) - Buy (To)**

As rural economies transition towards increased monetisation, there is a noticeable shift away from subsistence fishing practices, which were traditionally dedicated towards home consumption or meeting social obligations. Instead, there is a growing trend of trading fish for cash. Furthermore, the accessibility of tinned tuna, typically consisting of second-grade tuna and flesh only, poses a challenge to the consumption of more traditionally caught small reef fish. These smaller fish are often

consumed whole, providing a richer source of micronutrients compared to consuming only the flesh. The reliance on tinned tuna flesh is exacerbated by reduced access to wild reef fish populations due to factors such as overfishing and climate change. In regions where subsistence fishing remains prevalent, fish catch is primarily distributed directly to consumers, retail outlets, and restaurants. While most finfish catch is consumed locally or shared with relatives, only a small portion is reported to be sold, primarily through informal roadside markets. [38, 71, 20]

**Family farm (From) - Buy (To)**

Smallholder family farmers' production strategies typically utilise a mixture of root crop, vegetable, fruit, and livestock varieties for domestic consumption, sale, and gifts.

**Buy (From) - Gift (To)**

Adolescents pooled their limited resources to purchase food and share it among them.

**Grow (From) - State (To)**

Urban and peri-urban gardeners often donate food to schools, nurseries, nursing homes, and neighbours, particularly those in less fortunate circumstances. To address the limited market access for farmers, some governments established connections between farmers and schools, allowing farmer groups to supply the feeding programs. Efforts were also made to enhance the nutrition content of school meals by strengthening market linkages with farmer organisations. Similar initiatives were implemented in government institutions like prisons and hospitals. The garden was used for food production for school children and/or the local community. [54, 52, 58, 60, 67]

**Fish (From) - Gift (To)**

Fishers in some communities prioritise giving away part of their catch to family, friends, and neighbours instead of selling it, emphasising community relationships and solidarity. [46, 38, 74, 71, 40, 172, 166]

**Grow (From) - Aid (To)**

In the World Food Programme (WFP)'s flagship program, all the fresh produce is supplied by local smallholder farmers. In cases where both local and imported food are distributed within the program, communities express satisfaction with the chance to sell a portion of their local harvest, which not only benefits their children but also generates extra income. This is particularly valuable in rural regions where farmers face considerable challenges due to limited access to markets. [103, 101]

**Family farm (From) - Feast (To)**

Livestock from family farming also had a socio-cultural role as it was kept for special occasions and contributions to community feasts. [18, 149]

**Fish (From) - Marketplace (To)**

The fish caught by underwater spearfishing are mostly used for family consumption, although sometimes it is sold at the town market. [71, 32, 76]

**Family farm (From) - Marketplace (To)**

The aim of the family farms is self-sufficiency, with a portion of the produce serving as a source of livelihood through sales in local markets or at the family farm stall. Production is both consumed by the family and sold to the cooperative or local market. [104, 144, 157]

**Grow (From) - Informal grocery (To)**

During the COVID-19 pandemic, there has been a noticeable rise in the number of families selling their vegetables and fruits both at local markets and roadside stands near their homes. By setting up small fruit and vegetable stalls in front of their houses and offering produce from their own gardens, these families provided their communities with access to fresh produce without the need to travel to distant main markets. [108]

**Fish (From) - Feast (To)**

Fishing surplus to meet social/church obligations (such as offerings) or community festivities. [71, 76, 108, 32, 46]

**Fish (From) - Hospitality (To)**

Preparations with surplus fish (such as seafood packs, fish pies, etc.) are sold at ferry terminals and resorts. Fishing surplus is also sold at family-owned seafood restaurants. Value-added fish products from catches are sold in popular outlets, such as kava bars. [76, 71]

**Feast (From) - State (To)**

Some churches are even offering their own land to households without access to land and mobilising their community-based organizations to address food insecurity. Well-organised programs, such as breakfast programs for school children, home-delivered meals for seniors, and food baskets for families provided by churches, have been established. However, these programs face financial vulnerability, making it crucial to ensure sustainable funding. [47]

**Grow (From) - Remit (To)**

Urban dwellers ensure food security by relying on provisions and meats grown and provided by family and friends in rural communities. [139]

**Share (From) - Community (To)**

Food sharing initiatives such as the Singapore Food Bank.

**Community farm (From) - Marketplace (To)**

Produce from community farms is typically divided among farmers and often sold directly at local farmers' markets or through food box schemes, which not only contribute to the local food supply but also help fund garden operations. [163, 66, 35, 164, 52]

**Home garden (From) - Buy (To)**

Food from the home garden contributes to saving on food purchases, used for other food purchases such as fish, meat, eggs, or dairy products to supplement protein-deficient diets. [111, 48, 35, 15, 130]

**Informal grocery (From) - Gift (To)**

Students bought school lunches from a variety of school food vendors and share it among their friends. [49]

**Marketplace (From) - Feast (To)**

Baskets of taro and sweet potatoes of varieties that were not grown in the family's garden, were purchased from the market in town for feasting. [50]

**Remit (From) - Gift (To)**

When raw, unprocessed food products are sent from remote islands, for example, these are then distributed to siblings and friends. [32]

**Grocery (From) - Feast (To)**

Store-bought imported food is not commonly shared in the way that local food is. Imported food tends to be shared among families only during times of need or at feasts and special occasions; the cultural imperative to share does not appear to apply as strongly to imported food as to local food. [18]

**Community farm (From) - State (To)**

Community gardens made regular donations to schools, nursery, and nursing homes. [52]

**Family farm (From) - Informal grocery (To)**

Family farming is self-sufficiency with part of the produce being used for extended family own consumption and the other part as a source of livelihoods from selling to

local markets or family farm stall. Production is sold at the farm gate by the family members. Several gardeners explained that after they make it known through word of mouth that they sell produce, neighbours typically approach them at their homes. One household had a table just outside the front door with a sign indicating what was for sale and at what price. [114, 152, 161]

## Reported food items by food groups

This table reports all food items by food source sub-type that we extracted from the evidence reviewed. The terms used for the food items are the same the authors used to report them. We coded food items by food groups following the FAO/WHO GIFT methodological document:

FAO & WHO. Global Individual Food consumption data Tool (GIFT): methodological document. https://www.fao.org/3/cb8809en/cb8809en.pdf (2017).

| Food source | Sub-type | Cereals and their products | Roots, tubers, plantains, and their products | Pulses, seeds and nuts and their products | Milk, eggs, and their products | Fish, shellfish, and their products | Meat and meat products | Insects, grubs, and their products | Vegetables and their products | Fruits and their products | Fats and oils | Spices and condiments | Beverages | Food supplements and medicinal foods | Sweets and sugars | Composite dishes | Savoury snacks | Other |
|---|---|---|---|---|---|---|---|---|---|---|---|---|---|---|---|---|---|---|
| Aid | **Community** | rice | sweet potato /kumara /kumala | beans | | | chicken meat<br>turkey meat<br>ham | | coleslaw<br>various vegetables | | | | | | | | | |
| | **Emergency** | rice<br>instant noodles (dry)<br>flour | | various seeds | | tinned fish | tinned meat | | | | | | | | biscuits<br>crackers<br>sugar | | | |
| | **Target** | bulgur<br>rice<br>rice seedlings<br>maize seedlings | taro /dasheen seedlings<br>cassava seedlings<br>sweet potato /kumara /kumala seedlings | beans<br>peanut<br>peanut butter<br>pigeon pea<br>cowpea seeds | | canned mackerel | | | duruka seedlings<br>eggplant seedlings<br>chilli seedlings<br>okra seedlings<br>tomato seedlings<br>bitter gourd seedlings | | vegetable oil | salt | | micronutrients powder | | | | |

| Food source | Sub-type | Cereals and their products | Roots, tubers, plantains, and their products | Pulses, seeds and nuts and their products | Milk, eggs, and their products | Fish, shellfish, and their products | Meat and meat products | Insects, grubs, and their products | Vegetables and their products | Fruits and their products | Fats and oils | Spices and condiments | Beverages | Food supplements and medicinal foods | Sweets and sugars | Composite dishes | Savoury snacks | Other |
|---|---|---|---|---|---|---|---|---|---|---|---|---|---|---|---|---|---|---|
| **Buy** | **Informal grocery** | rice<br>white bread<br>cornmeal<br>instant noodles (dry)<br>popcorn | plantain green banana<br>potato<br>yucca<br>sweet potato /kumara /kumala<br>cassava bread<br>yucca flatbread<br>boiled manioc | beans<br>canned beans<br>betel /areca nut<br>coconut milk<br>boiled beans<br>roasted peanuts | canned evaporated milk<br>powdered milk<br>carton milk<br>fresh milk<br>hard-boiled eggs | lobster<br>fresh tilapia<br>freshwater prawns<br>fresh fish | goat meat<br>poultry meat<br>tinned meat<br>processed meat<br>fresh meat | | carrots<br>tayota<br>lettuce<br>chard<br>radish<br>beetroot<br>cucumber<br>tomatoes<br>spinach<br>pepper<br>onions<br>garlic<br>cabbage<br>auyama (pumpkin)<br>eggplant<br>rourou (local green leafy vegetable)<br>coliflower<br>broccoli<br>brussel sprouts<br>salad<br>canned vegetables<br>various fresh vegetables | fresh fruits<br>lime<br>lemon<br>banana<br>mango<br>papaya/pawpaw | cooking oil | chicken stock (cubes or liquid)<br>salsa<br>vanilla extract<br>various spices<br>various herbs | bottled drinking water<br>coffee<br>tea<br>rum<br>sweet<br>wines<br>local beer<br>kava drink<br>carbonated soft drinks<br>fruit-flavoured drinks<br>fruit drinks, shaved ice with syrup<br>pre-packaged juice<br>cocoa powder | | candy<br>chocolates<br>lollies<br>ice cream<br>cookies<br>baked buns<br>fried dough<br>deep fried pancakes<br>cakes<br>biscuits<br>pastries<br>fried banana<br>sweet starch<br>sugar<br>crackers | burritos<br>fried beans<br>fried horse meat<br>fried hot dog<br>bread with peanut butter<br>rice and beans<br>sweet potatoes with milk | Cheese Trix (artificially flavoured puffed corn) chips | |
| | **Grocery** | large bags of rice<br>cornmeal<br>instant noodles (dry)<br>pasta | potato | betel /areca nut | powdered milk<br>eggs | tinned fish<br>processed seafood<br>canned tuna | frozen meat<br>frozen poultry<br>canned meat | | onion<br>garlic<br>frozen vegetables<br>fresh vegetables | fresh fruits<br>processed fruits apples | catering-sized packs of cooking oil<br>catering-sized packs of butter | catering-sized packs of mayonnaise<br>spreads<br>sauces<br>salt | sweet drinks<br>bottled drinking water | | catering-sized packs of sugar<br>cookies<br>sweets<br>crackers | ready meals | savoury snacks | |

| Food source | Sub-type | Cereals and their products | Roots, tubers, plantains, and their products | Pulses, seeds and nuts and their products | Milk, eggs, and their products | Fish, shellfish, and their products | Meat and meat products | Insects, grubs, and their products | Vegetables and their products | Fruits and their products | Fats and oils | Spices and condiments | Beverages | Food supplements and medicinal foods | Sweets and sugars | Composite dishes | Savoury snacks | Other |
|---|---|---|---|---|---|---|---|---|---|---|---|---|---|---|---|---|---|---|
| | | flour<br><br>breakfast cereals | | | | | processed meats<br><br>chicken meat<br><br>imported meats | | processed vegetables | | | | | | | | | |
| | **Marketplace** | popcorn | plantain green banana<br><br>breadfruit<br><br>sweet potato /kumara /kumala<br><br>cassava | various nuts<br><br>snake bean | | fresh local fish<br><br>lobster<br><br>crab | pork<br><br>beef<br><br>poultry<br><br>goat (livestock)<br><br>chicken (livestock)<br><br>cows | | fresh local vegetables<br><br>cabbage<br><br>tomato<br><br>cucumber<br><br>capsicum /chili pepper<br><br>eggplant<br><br>spring onion | fresh local fruits | | various spices | | | white buns<br><br>coconut buns<br><br>cinnamon donuts | traditional fish puddings covered in coconut cream<br><br>fried fish and sweet potato chips<br><br>cassava pudding<br><br>taro leaf pudding<br><br>fish and chips<br><br>fried rice balls<br><br>fish pudding<br><br>sausage with cabbage<br><br>beans and rice<br><br>mais moulen (corn mush) | | |

| Food source | Sub-type | Cereals and their products | Roots, tubers, plantains, and their products | Pulses, seeds and nuts and their products | Milk, eggs, and their products | Fish, shellfish, and their products | Meat and meat products | Insects, grubs, and their products | Vegetables and their products | Fruits and their products | Fats and oils | Spices and condiments | Beverages | Food suppleme nts and medicinal foods | Sweets and sugars | Composite dishes | Savoury snacks | Other |
|---|---|---|---|---|---|---|---|---|---|---|---|---|---|---|---|---|---|---|
| | **Hospitality** | | | | | | | | | | | | coffee<br>tea<br>beer | | ice-cream | ready meals<br>traditional ethnic dishes<br>internation al dishes<br>burger<br>fries<br>pizza<br>sausages<br>steamed chicken buns (sow bow)<br>pies<br>cooked chicken<br>cooked fish<br>traditional fish puddings covered in coconut cream<br>bowl of noodles (la mien)<br>basket of dumplings (xiao long bao) | | organic fresh produce |
| | **Non-grocery** | | | | | | | | | | | | | | | ready meals<br>ultra-processed foods | | |

| Food source | Sub-type | Cereals and their products | Roots, tubers, plantains, and their products | Pulses, seeds and nuts and their products | Milk, eggs, and their products | Fish, shellfish, and their products | Meat and meat products | Insects, grubs, and their products | Vegetables and their products | Fruits and their products | Fats and oils | Spices and condiments | Beverages | Food supplements and medicinal foods | Sweets and sugars | Composite dishes | Savoury snacks | Other |
|---|---|---|---|---|---|---|---|---|---|---|---|---|---|---|---|---|---|---|
| **Grow** | **Home garden** | corn<br>maize | taro /dasheen<br>sweet potato /kumara /kumala<br>yam /elephant's foot yam<br>cassava<br>sago<br>breadfruit | coconut milk<br>betel/areca nut<br>chickpeas<br>various nuts<br>cowpea seeds<br>pigeon peas<br>coconut<br>copra | eggs | | ducks (livestock)<br>goats (livestock)<br>cows<br>chicken (livestock)<br>pigs<br>poultry (livestock) | | pakchoi<br>pepper<br>cabbage<br>tomatoes<br>capsicum /chili pepper<br>squash<br>melange<br>zucchini<br>okra<br>pumpkin<br>various vegetables<br>dwarf beans | citrus fruits<br>papaya /pawpaw<br>guava<br>carambola<br>avocado<br>mango<br>watermelon<br>banana<br>various fruits | | various herbs | lemon leaf tea<br>kava | | | beans and rice<br>mais moulen (corn mush) | | |

| Food source | Sub-type | Cereals and their products | Roots, tubers, plantains, and their products | Pulses, seeds and nuts and their products | Milk, eggs, and their products | Fish, shellfish, and their products | Meat and meat products | Insects, grubs, and their products | Vegetables and their products | Fruits and their products | Fats and oils | Spices and condiments | Beverages | Food suppleme nts and medicinal foods | Sweets and sugars | Composite dishes | Savoury snacks | Other |
|---|---|---|---|---|---|---|---|---|---|---|---|---|---|---|---|---|---|---|
| | **Family farm** | rice<br>maize | taro /dasheen<br>sweet potato /kumara /kumala<br>cassava<br>yam/eleph ant's foot yam<br>plantain green banana<br>yucca<br>arrowroot | beans<br>peas<br>betel/areca nut<br>candlenut<br>peanuts<br>cashew nuts<br>copra (cash crop)<br>coconut<br>breadfruit<br>coconut products (cream) | milk<br>eggs | farmed fish tilapia<br>giant clam | cows<br>pigs<br>chicken (livestock)<br>poultry (livestock)<br>goat (livestock)<br>sheep meat (mutton) | | various vegetables<br>ginger<br>cabbage<br>tomatoes<br>cucumber<br>pumpkin<br>carrots<br>lettuce<br>capsicum /chili pepper<br>spring onions<br>seaweed<br>kasume (edible fern)<br>garlic<br>radishes<br>pepper<br>eggplant | various fruits<br>banana<br>papaya /pawpaw<br>pandanus<br>lime<br>mango<br>golden apple<br>sour orange<br>pineapple<br>melon<br>watermelon | | vanilla (cash crop)<br>various spices<br>various herbs | coffee (cash crop)<br>cocoa (cash crop)<br>kava | ora | sugarcane | | | |
| | **Community farm** | rice | yucca<br>taro /dasheen<br>yam /elephant's foot yam<br>sweet potato /kumara /kumala<br>potato<br>cassava | beans | eggs | | chicken (livestock)<br>pigs<br>goat (livestock)<br>duck (livestock)<br>rabbit<br>geese<br>guanaco<br>turkey (livestock) | | various vegetables<br>tomatoes<br>pepper<br>squash | various fruits<br>banana | | various spices<br>various herbs | coffee | | | | | |

| Food source | Sub-type | Cereals and their products | Roots, tubers, plantains, and their products | Pulses, seeds and nuts and their products | Milk, eggs, and their products | Fish, shellfish, and their products | Meat and meat products | Insects, grubs, and their products | Vegetables and their products | Fruits and their products | Fats and oils | Spices and condiments | Beverages | Food suppleme nts and medicinal foods | Sweets and sugars | Composite dishes | Savoury snacks | Other |
|---|---|---|---|---|---|---|---|---|---|---|---|---|---|---|---|---|---|---|
| **Share** | **Gift** | rice<br>cereals<br>instant noodles (dry)<br>maize | cassava<br>plantain green banana | coconut | milk<br>eggs | fresh fish<br>shellfish<br>canned fish | red meat<br>chicken meat | | various vegetables<br>cucumber<br>leafy greens | mango<br>golden apple<br>sour orange<br>lime | | | soft sugary drinks | | sweet snacks<br>pancakes<br>cakes<br>sugar | prepared meals | savoury snacks | |
| | **Feast** | | taro /dasheen<br>yam/ elephant's foot yam<br>sweet potato /kumara /kumala<br>plantain green banana | beans | | fish | poultry (livestock)<br>sheep meat (mutton)<br>fresh meat<br>lamb flaps<br>beef (mostly corned beef)<br>chicken meat<br>pork | | locally grown island vegetables<br>cauliflower | banana<br>grapes<br>cantaloupe<br>watermelon<br>orange<br>canned fruit salad | | | | | desserts<br>ice cream | laplap (pudding baked in an earth oven)<br>beef soaked in a spicy lemon sauce (beef kelaguin)<br>fried fish and fresh vegetables soaked in an acidic sauce (eskabechi)<br>taro leaves in coconut milk (hagun suni)<br>shrimp soaked in a spicy lemon sauce (shrimp kelaguin)<br>fresh beef seasoned and dried in the sun (tinala' katne)<br>stuffed ground pork (embotido)<br>chicken stir-fried with bittermelo | | |

| Food source | Sub-type | Cereals and their products | Roots, tubers, plantains, and their products | Pulses, seeds and nuts and their products | Milk, eggs, and their products | Fish, shellfish, and their products | Meat and meat products | Insects, grubs, and their products | Vegetables and their products | Fruits and their products | Fats and oils | Spices and condiments | Beverages | Food suppleme nts and medicinal foods | Sweets and sugars | Composite dishes | Savoury snacks | Other |
|---|---|---|---|---|---|---|---|---|---|---|---|---|---|---|---|---|---|---|
| | | | | | | | | | | | | | | | | n (chicken ampalaya)<br><br>oxtail cooked with vegetables and peanut butter (kare kare)<br><br>rice noodles topped with eggs, meat flavorings, and special sauce (palabok) | | |
| | **Barter** | rice<br><br>instant noodles (dry) | | | | tin taiyo (canned mackerel)<br><br>fresh fish<br><br>lobster | piglets (livestock) | | garden produced vegetables | garden produced fruits | | | | | brownies<br><br>home made scones | homemade pies | | groceries |
| | **Remit** | | breadfruit<br><br>pulaka | coconut | | fresh fish<br><br>shellfish<br><br>dried fish | wild game meat | | fresh vegetables<br><br>leafy greens | fresh fruits<br><br>banana | | | | | | | | store-bought imported foods |
| **State** | **School meals** | bread<br><br>granola<br><br>cereal bars | cassava<br><br>imported potato<br><br>local sweet potato /kumara /kumala | beans | milk (fortified) milk-based drink | fish | chicken meat pork | | imported carrots<br><br>cabbage<br><br>local tomatoes<br><br>coriander<br><br>eggplant<br><br>imported onions<br><br>local cucumbers<br><br>local string beans | local and seasonal citrus fruits<br><br>fresh fruits<br><br>local watermelon<br><br>local bananas<br><br>local tangerines<br><br>local oranges | | | local juice<br><br>(fortified) juice<br><br>(fortified) cereal-based drink | | local biscuits<br><br>cookies<br><br>buns (Nutribun) | | | |
| | **Baskets** | rice | | | milk | | animal products | | | fresh fruits | cooking oil | | coffee | | sugar | | | |

| Food source | Sub-type | Cereals and their products | Roots, tubers, plantains, and their products | Pulses, seeds and nuts and their products | Milk, eggs, and their products | Fish, shellfish, and their products | Meat and meat products | Insects, grubs, and their products | Vegetables and their products | Fruits and their products | Fats and oils | Spices and condiments | Beverages | Food supplements and medicinal foods | Sweets and sugars | Composite dishes | Savoury snacks | Other |
|---|---|---|---|---|---|---|---|---|---|---|---|---|---|---|---|---|---|---|
| | | flour | | | (fortified) milk-based drink | | | | various fresh vegetables | | | | | | | | | |
| | **Assistance** | rice | | beans | | | chicken meat<br>turkey meat<br>ham | | coleslaw | | | | | | | | | |
| **Wild** | **Forage** | | wild yam /elephant's foot yam<br>wild taro /dasheen<br>plantain green banana<br>sago starch<br>sago palm<br>cassava<br>sweet potato /kumara /kumala<br>burkill /kumbili<br>breadfruit | bitter bean<br>Tahitian chesnut<br>coconut<br>various nuts | | | | | mushrooms<br>leafy greens<br>bele (spinach)<br>boreti (mangrove fern)<br>ota (edible fern)<br>other ferns | various fruits<br>banana<br>papaya /pawpaw<br>pandanus<br>maripa<br>awara<br>balata<br>hog plum<br>serrette | | | fresh water | wild plants<br>moca<br>edible bird's nests | | | | |

| Food source | Sub-type | Cereals and their products | Roots, tubers, plantains, and their products | Pulses, seeds and nuts and their products | Milk, eggs, and their products | Fish, shellfish, and their products | Meat and meat products | Insects, grubs, and their products | Vegetables and their products | Fruits and their products | Fats and oils | Spices and condiments | Beverages | Food supplements and medicinal foods | Sweets and sugars | Composite dishes | Savoury snacks | Other |
|---|---|---|---|---|---|---|---|---|---|---|---|---|---|---|---|---|---|---|
| | **Hunt** | | | | | | wild boars (vuaka)<br>wild pigs<br>monkey<br>cockatoo<br>bush fowl<br>turtledove<br>opossum<br>reptile<br>deer<br>buffalo<br>lizard<br>wild cat<br>birds<br>bats<br>rodents | insects<br>worms | | | | | | | | | | |
| | **Fish** | | | | | surgeonfish | turtle | mangrove worms | seaweeds algae | | | | | | | | | |

| Food source | Sub-type | Cereals and their products | Roots, tubers, plantains, and their products | Pulses, seeds and nuts and their products | Milk, eggs, and their products | Fish, shellfish, and their products | Meat and meat products | Insects, grubs, and their products | Vegetables and their products | Fruits and their products | Fats and oils | Spices and condiments | Beverages | Food suppleme nts and medicinal foods | Sweets and sugars | Composite dishes | Savoury snacks | Other |
|---|---|---|---|---|---|---|---|---|---|---|---|---|---|---|---|---|---|---|
| | | | | | | grouper<br>mullet<br>carangids<br>rabbitfish<br>beche-de-mer /sea cucumber<br>octopus<br>lobster<br>mud /mangrove crab<br>trochus<br>turbo<br>giant clams<br>anadara<br>prawns<br>eel<br>emperors<br>mussels<br>milkfish<br>snappers<br>trevally<br>tuna<br>matam"u<br>gague<br>taiva<br>mataeleele | | | | | | | | | | | | |

## References


10. Haynes, E. et al. Food Sources and Dietary Quality in Small Island Developing States: Development of Methods and Policy Relevant Novel Survey Data from the Pacific and Caribbean. Nutrients 12, 3350 (2020)

11. Savage, A., Bambrick, H. & Gallegos, D. Climate extremes constrain agency and long-term health: A qualitative case study in a Pacific Small Island Developing State. Weather and Climate Extremes 31, 100293 (2021)

12. Wentworth, C. Unhealthy Aid: Food Security Programming and Disaster Responses to Cyclone Pam in Vanuatu. Anthropological Forum 30, 73–90 (2020)

13. Thomas, K., Rosenberger, J. G. & Pawloski, L. R. Food Security in Bombardopolis, Haiti. Journal of Hunger & Environmental Nutrition 9, 230–243 (2014)

14. Takasaki, Y. Targeting Cyclone Relief within the Village: Kinship, Sharing, and Capture. Economic Development and Cultural Change 59, 387–416 (2011)

15. Ballard, C. & Bourke, R. M. Planning ahead to reduce feast and famine after natural disasters. The Conversation (2015).

16. PAHO. Ultra-processed food and drink products in Latin America: Trends, impact on obesity, policy implications. (2015).

17. IFAD. Transforming rural areas in Asia and the Pacific. (2014).

18. McCubbin, S. G., Pearce, T., Ford, J. D. & Smit, B. Social–ecological change and implications for food security in Funafuti, Tuvalu. Ecology and Society 22, (2017).

19. Paddock, J. R. Changing consumption, changing tastes? Exploring consumer narratives for food secure, sustainable and healthy diets. Journal of Rural Studies 53, 102–110 (2017).

20. Vogliano, C. et al. Dietary agrobiodiversity for improved nutrition and health outcomes within a transitioning indigenous Solomon Island food system. Food Sec. 13, 819–847 (2021).

21. FAO. An assessment of the Impact of climate change on agriculture and food security in the Pacific. A case study in the Cook Islands. (2008).

22. Johns, C., Lyon, P., Stringer, R. & Umberger, W. Changing urban consumer behaviour and the role of different retail outlets in the food industry of Fiji. Asia-Pacific Development Journal 24, 117–145 (2017).

23. Hippert, C. The moral economy of corner stores, buying food on credit, and Haitian-Dominican interpersonal relations in the Dominican Republic. Food and Foodways 25, 193–214 (2017).

24. Sadi, M. A. Restaurant Patronage and the Ethnic Groups in Singapore: An Exploratory Investigation Using Barker's Model. Journal of Foodservice Business Research 5, 79–99 (2002).

25. Mele, C., Ng, M. & Chim, M. B. Urban markets as a 'corrective' to advanced urbanism: The social space of wet markets in contemporary Singapore. Urban Studies 52, 103–120 (2015).

26. Vogliano, C. et al. Assessing diet quality of indigenous food systems in three geographically distinct solomon islands sites (Melanesia, Pacific Islands). Nutrients 13, 30 (2020).
27. Medina Hidalgo, D. et al. Sustaining healthy diets in times of change: linking climate hazards, food systems and nutrition security in rural communities of the Fiji Islands. Reg Environ Change 20, 73 (2020).
28. FAO & PAHO. Panorama of Food and Nutritional Security in Latin America and the Caribbean. (2017).
29. Reti, M. J. An assessment of the Impact of climate change on agriculture and food security in the Pacific. A Case Study in the Republic of the Marshall Islands. (FAO, 2008).
30. International Fund for Agricultural Development. Enabling poor rural people to overcome poverty in Seychelles. (2013).
31. Savage, A., Bambrick, H. & Gallegos, D. From garden to store: local perspectives of changing food and nutrition security in a Pacific Island country. Food Sec. 12, 1331–1348 (2020).
32. Serra Mallol, C. Monetary income, public funds, and subsistence consumption: the three components of the food supply in French Polynesia — a comparative study of Tahiti and Rapa Iti islands. Rev Agric Food Environ Stud 99, 37–55 (2018).
33. Minter T, Orirana G, Boso D and van der Ploeg J. 2018. From happy hour to hungry hour: Logging, fisheries and food security in Malaita, Solomon Islands. Penang, Malaysia: WorldFish. Program Report: 2018-07.
34. Shah, S., Moroca, A. & Bhat, J. A. Neo-traditional approaches for ensuring food security in Fiji Islands. Environmental Development 28, 83–100 (2018).
35. Warner, L. A., Harder, A. M., Henry, C. V., Ganpat, W. G. & Martin, E. Factors That Influence Engagement in Home Food Production: Perceptions of Citizens of Trinidad. Journal of Agricultural Education 58, 239–255 (2017).
36. da Costa, M. dJ et al. Household food insecurity in Timor-Leste. Food Sec. 5, 83–94 (2013).
37. WFP. Caribbean COVID-19 Food Security & Livelihoods Impact Survey. Dominica Summary Report. (2020).
38. FAO. Marine fishery resources of the Pacific Islands. (2010).
39. Sharma, K. L. Food Security in the South Pacific Island Countries with Special Reference to the Fiji Islands. in Food Insecurity, Vulnerability and Human Rights Failure (eds. Guha-Khasnobis, B., Acharya, S. S. & Davis, B.) 35–57 (Palgrave Macmillan UK, London, 2007).
40. WFP. Caribbean COVID-19 Food Security & Livelihoods Impact Survey. Saint Lucia Summary Report. (2020).
41. WFP. Adolescent Nutrition in Timor-Leste. (2019).
42. Kinlocke, R. et al. The state of household food security in Kingston, Jamaica. (Hungry Cities Partnership, 2019).
43. Rut, M. Thank You Food Sharers of Singapore! SHARE CITY (2017).

44. Boodoosingh, R. Bartering in Samoa during COVID-19. Devpolicy Blog (2020).
45. Randin, G. COVID-19 and Food Security in Fiji: The Reinforcement of Subsistence Farming Practices in Rural and Urban Areas. Oceania 90, (2020).
46. Bender, A. Changes in social orientation: Threats to a cultural institution in marine resource exploitation in Tonga. Human organization 66, 11–21 (2007).
47. Wright, L. & Epps, J. Coping strategies, their relationship to weight status and food assistances food programs utilized by the food-insecure in Belize. in 2015 5th International Conference on Biomedical Engineering and Technology vol. 81 66–73 (2015).
48. Iese, V. et al. Impacts of COVID-19 on agriculture and food systems in Pacific Island countries (PICs): Evidence from communities in Fiji and Solomon Islands. Agricultural Systems 190, 103099 (2021).
49. Cacavas, K. et al. Tongan Adolescents' Eating Patterns: Opportunities for Intervention. Asia Pac J Public Health 23, 24–33 (2011).
50. Wentworth, C. Public eating, private pain: Children, feasting, and food security in Vanuatu. Food and Foodways 24, 136–152 (2016).
51. Paulino, Y. C., Guerrero, R. T. L. & Novotny, R. Women in Guam consume more calories during feast days than during non-feast days. Micronesica 41, 223 (2011).
52. Moskow A. Havana's self-provision gardens. Environment and Urbanization. 11, 127-134 (1999).
53. Kraemer, D., 2017. Family relationships in town are brokbrok: Food sharing and "contribution" in Port Vila, Vanuatu. Journal de la Société des Océanistes. 144, 105–116 (2017).
54. Bryceson, K. P. & Ross, A. Habitus of informality in small scale society agrifood chains – filling the knowledge gap using a socio-culturally focused value chain analysis tool. Journal of the Asia Pacific Economy 25, 545–570 (2020).
55. Lako, J. V. & Nguyen, V. C. Dietary patterns and risk factors of diabetes mellitus among urban indigenous women in Fiji. Asia Pac J Clin Nutr 10, 188–193 (2001).
56. FAO, IFAD, PAHO, UNICEF, & WFP. Regional Overview of Food Security and Nutrition – Latin America and the Caribbean 2020: Towards Improving Affordability of Healthy Diets. (2020).
57. Burkhart, S. School Nutrition Education Programmes in the Pacific Islands: Scoping Review and Capacity Needs Assessment: Final Report. (FAO, 2019).
58. FAO. Crop and Food Supply Assessment Mission (CFSAM) to the Democratic Republic of Timor-Leste. (2021).
59. FAO. COVID-19 and the Role of Local Food Production in Building More Resilient Local Food Systems. (2020).
60. FAO. Strengthening the Capacity of Farmers and Food Vendors to Supply Safe Nutritious Food in Guadalcanal, Malaita and Temotu Provinces of Solomon Islands - TCP/SOI/3601. (2020).

61. Beazley, R., Ciardi, F. & Bailey, S. Shock Responsive Social Protection in the Caribbean. Synthesis Report. (WFP, 2020).
62. Bailey, S. & Ciardi, F. Shock Responsive Social Protection in the Caribbean. Belize Case Study. (WFP, 2020).
63. Bailey, S. & Ciardi, F. Shock Responsive Social Protection in the Caribbean. Guyana Case Study. (WFP, 2020).
64. WFP. Smart School Meals: Nutrition-sensitive National Programmes in Latin America and the Caribbean - A Review of 16 Countries. (2017).
65. CARPHA. Healthy Ageing in the Caribbean. State of Public Health Report. (2019).
66. Franck, V. Summer Edition: Food in Cuba! SHARE CITY (2019).
67. Burkhart, S. The Role of Diets and Food Systems in the Prevention of Obesity and Non-Communicable Diseases in Fiji: Gathering Evidence and Supporting Multi-Stakeholder Engagement. (FAO, 2021).
68. Reeser, D. C. "They don't garden here": NGO constructions of Maya gardening practices in Belize. Development in Practice 23, 799–810 (2013).
69. Gulliford, M. C., Mahabir, D., Rocke, B., Chinn, S. & Rona, R. J. Free school meals and children's social and nutritional status in Trinidad and Tobago. Public Health Nutrition 5, 625–630 (2002).
70. Walker, S. P., Powell, C. A., Hutchinson, S. E., Chang, S. M. & Grantham-McGregor, S. M. Schoolchildren's diets and participation in school feeding programmes in Jamaica. Public Health Nutrition 1, 43–49 (1998).
71. FAO. Fisheries of the Pacific Islands. Regional and national information. (2011).
72. David, G. Village fisheries in the Pacific Islands. in Proceedings of Socioeconomics, Innovation and Management of the Java Sea Pelagic Fisheries. Seminar SOSEKIMA. (eds. Roch J., S. Nurhakim,.I. Widodo, and A. Poernomo) 63–80 (1995).
73. Watson, M. S., Claar, D. C. & Baum, J. K. Subsistence in isolation: Fishing dependence and perceptions of change on Kiritimati, the world's largest atoll. Ocean & coastal management 123, 1–8 (2016).
74. FAO & SPC. Fishery and Aquaculture Economics and Policy Division. Report of the FAO/SPC Pacific Islands Regional Consultation on the Development of Guidelines for Securing Sustainable Small-Scale Fisheries, Noumea, New Caledonia, 12-14 June 2012. (2012).
75. Campbell, D. et al. Wild Food Harvest, Food Security, and Biodiversity Conservation in Jamaica: A Case Study of the Millbank Farming Region. Front. Sustain. Food Syst. 5, (2021).
76. Thomas, A. et al. Why they must be counted: Significant contributions of Fijian women fishers to food security and livelihoods. Ocean & Coastal Management 205, 105571 (2021).
84. Smith, D. The relational attributes of marketplaces in post-earthquake Port-au-Prince, Haiti. Environment and Urbanization 31, 497–516 (2019).

85. Rut, M. Following sustainable food narratives in Singapore. SHARE CITY (2018).
99. FAO. FAO and partners help restore nutrition and agricultural livelihoods in the Pacific Islands. Vanuatu. (2016).
100. WFP. Decentralized Evaluation. Final evaluation of WFP Haiti's Food for Education and Child Nutrition Programme (2016-2019). (2019).
101. WFP. Decentralized Evaluation. Final Evaluation of McGovern-Dole International Food for Education and Child Nutrition Program in Guinea-Bissau (2016-2019). (2021).
102. Committee on World Food Security. Regional Initiatives. Pacific Food Summit. (2010).
103. WFP. Strengthening National Safety Nets. School Feeding: WFP's Evolving Role in Latin America and the Caribbean.(2016).
104. FAO. State of food insecurity in the CARICOM Caribbean. (2015).
105. Thomas-Hope, E., Kinlocke, R., Ferguson, T., Heslop-Thomas, C. & Timmers, B. The Urban Food System of Kingston, Jamaica. (Hungry Cities Partnership, 2017).
106. FAO. CARICOM Food Import Bill, Food Security and Nutrition. (2013).
107. Hungry Cities Partnership. An Urban Perspective on Food Security in the Global South. (2018).
108. Emiliata, T. et al. Capturing the Experiences of Samoa: The Changing Food Environment and Food Security in Samoa during the COVID-19 Pandemic. Oceania 90, 116–125 (2020).
109. Narine, T. & Badrie, N. Influential Factors Affecting Food Choices of Consumers When Eating Outside the Household in Trinidad, West Indies. Journal of Food Products Marketing 13, 19–29 (2007).
110. Barr, S. Using Mixed Methods to Describe a Spatially Dynamic Food Environment in Rural Dominican Republic. Hum Ecol 45, 845–851 (2017).
111. Thomas, G. Growing Greener Cities in Latin America and the Caribbean: An FAO Report on Urban and Peri-Urban Agriculture in the Region. (FAO, 2014).
112. Morshed, A. B., Becker, H. V., Delnatus, J. R., Wolff, P. B. & Iannotti, L. L. Early nutrition transition in Haiti: linking food purchasing and availability to overweight status in school-aged children. Public health nutrition 19, 3378–3385 (2016).
113. Kinlocke, R. & Thomas-Hope, E. Inclusive growth and the informal food sector in Kingston, Jamaica. (Hungry Cities Partnership, 2020).
114. WFP. Caribbean COVID-19 Food Security & Livelihoods Impact Survey - Barbados Summary Report. (2020).
115. FAO. Enhancing evidence-based decision making for sustainable agriculture sector development in Pacific Islands Countries. (2010).
116. FAO. Documentation of the traditional food system of Pohnpei. (2009).
117. WFP. Caribbean COVID-19 Food Security & Livelihoods Impact Survey - Regional Summary Report. (2020).

118. Bottcher, C., Underhill, S. J. R., Aliakbari, J. & Burkhart, S. J. Food Access and Availability in Auki, Solomon Islands. Journal of Hunger & Environmental Nutrition 16, 751–769 (2021).
119. WHO. Healthy marketplaces in the Western Pacific: guiding future action: applying a settings approach to the promotion of health in marketplaces. (2014).
120. Gordons Market, Port Moresby officially opens. UN Women – Asia-Pacific (2019).
121. Making markets safe for women vendors in Papua New Guinea. UN Women – Headquarters (2014).
122. FAO. The Right to Food in the CARICOM Region: An Assessment Report. (2013).
123. Bottcher, C., Underhill, S. J., Aliakbari, J. & Burkhart, S. J. Food purchasing behaviors of a remote and rural adult Solomon islander population. Foods 8, 464 (2019).
124. WFP. Caribbean COVID-19 Food Security & Livelihoods Impact Survey - Belize Summary Report. (2020).
125. Hongjun, W. Food and the Singapore young consumer. Young Consumers 7, 53–59 (2006).
126. Naidoo, N. et al. Determinants of eating at local and western fast-food venues in an urban Asian population: a mixed methods approach. Int J Behav Nutr Phys Act 14, 69 (2017).
127. FAO. Mainstreaming Ecosystem Services and Biodiversity into Agricultural Production and Management in the Pacific Islands: Technical Guidance Document. (2016).
128. Rosset, P. & Benjamin, M. Two Steps Back, One Step Forward: Cuba's National Policy for Alternative Agriculture. (International Institute for Environment and Development (IIED), Sustainable Agriculture Programme, London, 1994).
129. FAO. FAO and SIDS: Challenges and Emerging Issues in Agriculture, Forestry and Fisheries: Paper Prepared by FAO on the Occasion of the Inter-Regional Conference of Small Island Developing States, Bahamas, 26-30 January 2004. (2004).
130. Thaman, R. R. Urban food gardening in the Pacific Islands: A basis for food security in rapidly urbanising small-island states. Habitat International 19, 209–224 (1995).
131. Valstar, A. Home-based food production in urban Jamaica. (FAO, 1999).
132. FAO. Pacific islands and FAO achievements and success stories. (2011).
133. Hibi, E., Lam, F. & Chopin, F. Accelerating Action on Food Security and Nutrition in Pacific Small Island Developing States (SIDS). (FAO, 2018).
134. Caribbean Agribusiness. Urban and Peri-urban Agriculture in Latin America and the Caribbean. Antigua and Barbuda. (2015).
135. Thomas-Hope, E., Kinlocke, R. & Ferguson, T. Nr. 09: Enhancing food security through urban agriculture in Kingston, Jamaica. (Hungry Cities Partnership, 2020).
136. WHO. Diet, food supply and obesity in the Pacific. (2003).

137. Craven, L. K. & Gartaula, H. N. Conceptualising the Migration–Food Security Nexus: Lessons from Nepal and Vanuatu. Australian Geographer 46, 455–471 (2015).
138. WFP. Caribbean COVID-19 Food Security & Livelihoods Impact Survey - Trinidad and Tobago Summary Report. (2020).
139. WFP. Caribbean COVID-19 Food Security & Livelihoods Impact Survey - Jamaica Summary Report. (2020).
140. WFP. Caribbean COVID-19 Food Security & Livelihoods Impact Survey - Bahamas Summary Report. (2020).
141. WFP. Caribbean COVID-19 Food Security & Livelihoods Impact Survey - Grenada Summary Report. (2020).
142. WFP. Caribbean COVID-19 Food Security & Livelihoods Impact Survey - British Virgin Islands Summary Report. (2020).
143. Garcia-Montiel, D. C. et al. Food sources and accessibility and waste disposal patterns across an urban tropical watershed: implications for the flow of materials and energy. Ecology and Society 19, (2014).
144. Lucantoni, D. Transition to agroecology for improved food security and better living conditions: case study from a family farm in Pinar del Río, Cuba. Agroecology and Sustainable Food Systems 44, 1124–1161 (2020).
145. Iimi, A. Hidden Treasures in the Comoros: The Impact of Inter-Island Connectivity Improvement on Agricultural Production. (The World Bank, 2019).
146. FAO. Food Security and Nutrition in Small Island Developing States (SIDS). (2014).
147. FAO. Improving the Capacity of Farmers to Market a Consistent Supply of Safe, Quality Food - TCP/SAM/3601. (2020).
148. FAO. Agriculture for Growth: learning from experience in the Pacific. (2010).
149. Rodríguez, D. I., Anríquez, G. & Riveros, J. L. Food security and livestock: The case of Latin America and the Caribbean. Ciencia e investigación agraria 43, 5–15 (2016).
150. WFP. Caribbean COVID-19 Food Security & Livelihoods Impact Survey - St. Kitts and Nevis Summary Report. (2020).
151. FAO. Global Blue Growth Initiative and Small Island Developing States (SIDS). (2014).
152. Sherzad, S. Family Farming in the Pacific Islands Countries: Challenges and Opportunities. (FAO, 2018).
153. Beyer, R. & Stice, K. FAO Roundtable on the competitiveness of Pacific Island Small and Medium agro-processing enterprises, 11-13 April 2012, Nadi, Fiji Islands. (FAO, 2012).
154. Bryceson, K. P. & Ross, A. Agrifood chains as complex systems and the role of informality in their sustainability in small scale societies. Sustainability 12, 6535 (2020).
155. Dean, G., Lyons, G. & Edis, R. How food gardens based on traditional practice can improve health in the Pacific. The Conversation (2017).

156. Opio, F. Contribution of subsistence diets to farm household nutrient requirements in the pacific. Ecology of Food and Nutrition 29, 285–305 (1993).
157. FAO. Strengthened Household Agroforestry and Food Production in Nauru - TCP/NAU/3501. (FAO, 2019).
158. IFAD. Investing in rural people in Papua New Guinea. (2020).
159. WFP. Caribbean COVID-19 Food Security & Livelihoods Impact Survey - Guyana Summary Report. (2020).
160. IFAD. Investing in rural people in Guinea-Bissau. (2019).
161. Chaplowe, S. G. Havana's popular gardens:sustainable prospects for urban agriculture. The Environmentalist 18, 47–57 (1998).
162. McIlvaine-Newsad, H., Porter, R. & Delany-Barmann, G. Change the game, not the rules: The role of community gardens in disaster resilience. Journal of Park and Recreation Administration 38, 2–22 (2020).
163. Grieg-Gran, M., Guijt, I. & Peutalo, B. Local Perspectives on Forest Values in Papua New Guinea - The Scope for Participatory Methods. (International Institute for Environment and Development, 2002).
164. Altieri, M. A. et al. The greening of the "barrios": Urban agriculture for food security in Cuba. Agriculture and Human Values 16, 131–140 (1999).
165. RUAF. Enhancing the Contribution of Urban Agriculture to Food Security. Urban Agriculture Magazine Special Issue (2002).
166. García-Quijano, C. G. & Lloréns, H. What rural, coastal Puerto Ricans can teach us about thriving in times of crisis. The Conversation (2017).
167. Davies et al. Singapore SHARECITY Profile. (SHARE CITY, 2017).
168. Troubat, N., Faaola, E. and Aliyeva, R. 2020. Food security and food consumption in Samoa – Based on the analysis of the 2018 Household Income and Expenditure Survey. Apia, FAO and SBS. https://doi.org/10.4060/cb0613en
169. FAO & SPC. Solomon Islands Food Security Profile. (2020).
170. FAO. Tonga: Food Security Profile. (2019).
171. KNSO, FAO & SPC. Kiribati Food Security Profile. (2021).
172. FAO, Pacific Community & Tuvalu Central Statistics Division. Tuvalu Food Security Profile. (2022).
173. Tora, T. Two piglets for a kayak: Fiji returns to barter system as Covid-19 hits economy. The Guardian (2020).
174. Siutaia, H. Le Barter trading platform glimpse of the past. Samoa Observer (2020).
175. Maeir AM. A Feast in Papua New Guinea. Near Eastern Archaeology 78, 26-34 (The University of Chicago Press, 2015).
176. Erskine, W. et al. The role of wild foods in food security: the example of Timor-Leste. Food Sec. 7, 55–65 (2015).
177. FAO. Natural Resources Management and the Environment in Small Island Developing States (SIDS). (2014).
178. FAO. Forest and Forestry in Small Island Developing States. (2002).

179. FAO. Disaster risk management and climate change adaptation in the CARICOM and wider Caribbean region. Strategy and action plan. (2015).
180. FAO. Management of large pelagic fisheries in CARICOM countries. (2004).
181. Gillett, R., Moy, W., & Fishery and Aquaculture Economics and Policy Division. Spearfishing in the Pacific Islands. Current Status and Management Issues. (FAO, 2006).
