# Supplementary material for: Examining Food Sources and Their Interconnections over Time in Small Island Developing States: A Systematic Scoping Review
Source: Nutrients. 2025 Jul 18;17(14):2353. doi: 10.3390/nu17142353 (PMC12298424; doi:10.3390/nu17142353)
Supplement: Supplementary file 1 [file nutrients-17-02353-s001.zip › List of eligible SIDS.pdf]

#ID	SIDS	Region	UN member
1	American Samoa	Pacific	N
2	Anguilla	Caribbean	N
3	Antigua and Barbuda	Caribbean	Y
4	Aruba	Caribbean	N
5	Bahamas	Caribbean	Y
6	Bahrain	AIS	Y
7	Barbados	Caribbean	Y
8	Belize	Caribbean	Y
9	Bermuda	Caribbean	N
10	British Virgin Islands	Caribbean	N
11	Cabo Verde	AIS	Y
12	Cayman Islands	Caribbean	N
13	Commonwealth of Northe	Pacific	N
14	Comoros	AIS	Y
15	Cook Islands	Pacific	N
16	Cuba	Caribbean	Y
17	Curaçao	Caribbean	N
18	Dominica	Caribbean	Y
19	Dominican Republic	Caribbean	Y
20	Federated States of Micro	Pacific	Y
21	Fiji	Pacific	Y
22	French Polynesia	Pacific	N
23	Grenada	Caribbean	Y
24	Guadeloupe	Caribbean	N
25	Guam	Pacific	N
26	Guinea-Bissau	AIS	Y
27	Guyana	Caribbean	Y
28	Haiti	Caribbean	Y
29	Jamaica	Caribbean	Y
30	Kiribati	Pacific	Y
31	Maldives	AIS	Y
32	Marshall Islands	Pacific	Y
33	Martinique	Caribbean	N
34	Mauritius	AIS	Y
35	Montserrat	Caribbean	N
36	Nauru	Pacific	Y
37	New Caledonia	Pacific	N
38	Niue	Pacific	N
39	Palau	Pacific	Y
40	Papua New Guinea	Pacific	Y
41	Puerto Rico	Caribbean	N
42	Saint Kitts and Nevis	Caribbean	Y
43	Saint Lucia	Caribbean	Y
44	Saint Vincent and the Gre	Caribbean	Y

45	Samoa	Pacific	Y
46	São Tomé and Príncipe	AIS	Y
47	Seychelles	AIS	Y
48	Singapore	AIS	Y
49	Sint Maarten	Caribbean	N
50	Solomon Islands	Pacific	Y
51	Suriname	Caribbean	Y
52	Timor-Leste	Pacific	Y
53	Tonga	Pacific	Y
54	Trinidad and Tobago	Caribbean	Y
55	Turks and Caicos Islands	Caribbean	N
56	Tuvalu	Pacific	Y
57	Unites States Virgin Island	Caribbean	N
58	Vanuatu	Pacific	Y
59	Tokelau	Pacific	N
