# Supplementary material for: Examining Food Sources and Their Interconnections over Time in Small Island Developing States: A Systematic Scoping Review
Source: Nutrients. 2025 Jul 18;17(14):2353. doi: 10.3390/nu17142353 (PMC12298424; doi:10.3390/nu17142353)
Supplement: Supplementary file 1 [file nutrients-17-02353-s001.zip › List of included studies_citations.pdf]

Ref. Nr.	Evidence type	Citation
10	peer-reviewed	Haynes, E. et al. Food Sources and Dietary Quality in Small Island Developing States: Development of Methods and Policy Relevant Novel Survey Data from the Pacific and Caribbean. Nutrients 12, 3350 (2020)
11	peer-reviewed	Savage, A., Bambrick, H. & Gallegos, D. Climate extremes constrain agency and long-term health: A qualitative case study in a Pacific Small Island Developing State. Weather and Climate Extremes 31, 100293 (2021)
12	peer-reviewed	Wentworth, C. Unhealthy Aid: Food Security Programming and Disaster Responses to Cyclone Pam in Vanuatu. Anthropological Forum 30, 73–90 (2020)
13	peer-reviewed	Thomas, K., Rosenberger, J. G. & Pawloski, L. R. Food Security in Bombardopolis, Haiti. Journal of Hunger & Environmental Nutrition 9, 230–243 (2014)
14	peer-reviewed	Takasaki, Y. Targeting Cyclone Relief within the Village: Kinship, Sharing, and Capture. Economic Development and Cultural Change 59, 387–416 (2011)
15	grey literature	Ballard, C. & Bourke, R. M. Planning ahead to reduce feast and famine after natural disasters. The Conversation (2015).
16	grey literature	PAHO. Ultra-processed food and drink products in Latin America: Trends, impact on obesity, policy implications. (2015).
17	grey literature	IFAD. Transforming rural areas in Asia and the Pacific. (2014).
18	peer-reviewed	McCubbin, S. G., Pearce, T., Ford, J. D. & Smit, B. Social–ecological change and implications for food security in Funafuti, Tuvalu. Ecology and Society 22, (2017).
19	peer-reviewed	Paddock, J. R. Changing consumption, changing tastes? Exploring consumer narratives for food secure, sustainable and healthy diets. Journal of Rural Studies 53, 102–110 (2017).
20	peer-reviewed	Vogliano, C. et al. Dietary agrobiodiversity for improved nutrition and health outcomes within a transitioning indigenous Solomon Island food system. Food Sec. 13, 819–847 (2021).
21	grey literature	FAO. An assessment of the Impact of climate change on agriculture and food security in the Pacific. A case study in the Cook Islands. (2008).
22	peer-reviewed	Johns, C., Lyon, P., Stringer, R. & Umberger, W. Changing urban consumer behaviour and the role of different retail outlets in the food industry of Fiji. Asia-Pacific Development Journal 24, 117–145 (2017).
23	peer-reviewed	Hippert, C. The moral economy of corner stores, buying food on credit, and Haitian-Dominican interpersonal relations in the Dominican Republic. Food and Foodways 25, 193–214 (2017).
24	peer-reviewed	Sadi, M. A. Restaurant Patronage and the Ethnic Groups in Singapore: An Exploratory Investigation Using Barker’s Model. Journal of Foodservice Business Research 5, 79–99 (2002).
25	peer-reviewed	Mele, C., Ng, M. & Chim, M. B. Urban markets as a ‘corrective’ to advanced urbanism: The social space of wet markets in contemporary Singapore. Urban Studies 52, 103–120 (2015).
26	peer-reviewed	Vogliano, C. et al. Assessing diet quality of indigenous food systems in three geographically distinct solomon islands sites (Melanesia, Pacific Islands). Nutrients 13, 30 (2020).
27	peer-reviewed	Medina Hidalgo, D. et al. Sustaining healthy diets in times of change: linking climate hazards, food systems and nutrition security in rural communities of the Fiji Islands. Reg Environ Change 20, 73 (2020).
28	grey literature	FAO. Panorama of Food and Nutritional Security in Latin America and the Caribbean. (2017).
29	grey literature	Reti, M. J. An assessment of the Impact of climate change on agriculture and food security in the Pacific. A Case Study in the Republic of the Marshall Islands. (FAO, 2008).
30	grey literature	International Fund for Agricultural Development. Enabling poor rural people to overcome poverty in Seychelles. (2013).
31	peer-reviewed	Savage, A., Bambrick, H. & Gallegos, D. From garden to store: local perspectives of changing food and nutrition security in a Pacific Island country. Food Sec. 12, 1331–1348 (2020).
32	peer-reviewed	Serra Mallol, C. Monetary income, public funds, and subsistence consumption: the three components of the food supply in French Polynesia — a comparative study of Tahiti and Rapa Iti islands. Rev Agric Food Environ Stud 99, 37–55 (2018).
33	grey literature	Consultative Group on International Agricultural Research. From happy hour to hungry hour: Logging, fisheries and food security in Malaita, Solomon Islands. (2018).
34	peer-reviewed	Shah, S., Moroca, A. & Bhat, J. A. Neo-traditional approaches for ensuring food security in Fiji Islands. Environmental Development 28, 83–100 (2018).
35	peer-reviewed	Warner, L. A., Harder, A. M., Henry, C. V., Ganpat, W. G. & Martin, E. Factors That Influence Engagement in Home Food Production: Perceptions of Citizens of Trinidad. Journal of Agricultural Education 58, 239–255 (2017).
36	peer-reviewed	da Costa, M. dJ et al. Household food insecurity in Timor-Leste. Food Sec. 5, 83–94 (2013).
37	grey literature	WFP. Caribbean COVID-19 Food Security & Livelihoods Impact Survey. Dominica Summary Report. (2020).
38	grey literature	FAO. Marine fishery resources of the Pacific Islands. (2010).
39	grey literature	Sharma, K. L. Food Security in the South Pacific Island Countries with Special Reference to the Fiji Islands. in Food Insecurity, Vulnerability and Human Rights Failure (eds. Guha-Khasnobis, B., Acharya, S. S. & Davis, B.) 35–57 (Palgrave Macmillan UK, London, 2007).
40	grey literature	WFP. Caribbean COVID-19 Food Security & Livelihoods Impact Survey. Saint Lucia Summary Report. (2020).
41	grey literature	WFP. Adolescent Nutrition in Timor-Leste. (2019).
42	grey literature	Kinlocke, R. et al. The state of household food security in Kingston, Jamaica. (Hungry Cities Partnership, 2019).
43	grey literature	Rut, M. Thank You Food Sharers of Singapore! SHARE CITY (2017).
44	grey literature	Boodoosingh, R. Bartering in Samoa during COVID-19. Devpolicy Blog (2020).
45	peer-reviewed	Randin, G. COVID-19 and Food Security in Fiji: The Reinforcement of Subsistence Farming Practices in Rural and Urban Areas. Oceania 90, (2020).
46	peer-reviewed	Bender, A. Changes in social orientation: Threats to a cultural institution in marine resource exploitation in Tonga. Human organization 66, 11–21 (2007).
47	peer-reviewed	Wright, L. & Epps, J. Coping strategies, their relationship to weight status and food assistances food programs utilized by the food-insecure in Belize. in 2015 5th International Conference on Biomedical Engineering and Technology vol. 81 66–73 (2015).

48	peer-reviewed	Iese, V. et al. Impacts of COVID-19 on agriculture and food systems in Pacific Island countries (PICs): Evidence from communities in Fiji and Solomon Islands. Agricultural Systems 190, 103099 (2021).
49	peer-reviewed	Cacavas, K. et al. Tongan Adolescents' Eating Patterns: Opportunities for Intervention. Asia Pac J Public Health 23, 24–33 (2011).
50	peer-reviewed	Wentworth, C. Public eating, private pain: Children, feasting, and food security in Vanuatu. Food and Foodways 24, 136–152 (2016).
51	peer-reviewed	Paulino, Y. C., Guerrero, R. T. L. & Novotny, R. Women in Guam consume more calories during feast days than during non-feast days. Micronesica 41, 223 (2011).
52	peer-reviewed	Moskow A. Havana's self-provision gardens. Environment and Urbanization. 11, 127-134 (1999).
53	peer-reviewed	Kraemer, D., 2017. Family relationships in town are brokbrok: Food sharing and "contribution" in Port Vila, Vanuatu. Journal de la Société des Océanistes. 144, 105–116 (2017).
54	peer-reviewed	Bryceson, K. P. & Ross, A. Habitus of informality in small scale society agrifood chains – filling the knowledge gap using a socio-culturally focused value chain analysis tool. Journal of the Asia Pacific Economy 25, 545–570 (2020).
55	peer-reviewed	Lako, J. V. & Nguyen, V. C. Dietary patterns and risk factors of diabetes mellitus among urban indigenous women in Fiji. Asia Pac J Clin Nutr 10, 188–193 (2001).
56	grey literature	FAO, IFAD, PAHO, UNICEF, & WFP. Regional Overview of Food Security and Nutrition – Latin America and the Caribbean 2022: Towards Improving Affordability of Healthy Diets. (2023).
57	grey literature	Burkhart, S. School Nutrition Education Programmes in the Pacific Islands: Scoping Review and Capacity Needs Assessment: Final Report. (FAO, 2019).
58	grey literature	FAO. Crop and Food Supply Assessment Mission (CFSAM) to the Democratic Republic of Timor-Leste. (2021).
59	grey literature	FAO. COVID-19 and the Role of Local Food Production in Building More Resilient Local Food Systems. (2020).
60	grey literature	FAO. Strengthening the Capacity of Farmers and Food Vendors to Supply Safe Nutritious Food in Guadalcanal, Malaita and Temotu Provinces of Solomon Islands - TCP/SOI/3601. (2020).
61	grey literature	Beazley, R., Ciardi, F. & Bailey, S. Shock Responsive Social Protection in the Caribbean. Synthesis Report. (WFP, 2020).
62	grey literature	Bailey, S. & Ciardi, F. Shock Responsive Social Protection in the Caribbean. Belize Case Study. (WFP, 2020).
63	grey literature	Bailey, S. & Ciardi, F. Shock Responsive Social Protection in the Caribbean. Guyana Case Study. (WFP, 2020).
64	grey literature	WFP. Smart School Meals: Nutrition-sensitive National Programmes in Latin America and the Caribbean - A Review of 16 Countries. (2017).
65	grey literature	CARPHA. Healthy Ageing in the Caribbean. State of Public Health Report. (2019).
66	grey literature	Franck, V. Summer Edition: Food in Cuba! SHARE CITY (2019).
67	grey literature	Burkhart, S. The Role of Diets and Food Systems in the Prevention of Obesity and Non-Communicable Diseases in Fiji: Gathering Evidence and Supporting Multi-Stakeholder Engagement. (FAO, 2021).
68	peer-reviewed	Reeser, D. C. "They don't garden here": NGO constructions of Maya gardening practices in Belize. Development in Practice 23, 799–810 (2013).
69	peer-reviewed	Gulliford, M. C., Mahabir, D., Rocke, B., Chinn, S. & Rona, R. J. Free school meals and children's social and nutritional status in Trinidad and Tobago. Public Health Nutrition 5, 625–630 (2002).
70	peer-reviewed	Walker, S. P., Powell, C. A., Hutchinson, S. E., Chang, S. M. & Grantham-McGregor, S. M. Schoolchildren's diets and participation in school feeding programmes in Jamaica. Public Health Nutrition 1, 43–49 (1998).
71	grey literature	FAO. Fisheries of the Pacific Islands. Regional and national information. (2011).
72	grey literature	David, G. Village fisheries in the Pacific Islands. in Proceedings of Socioeconomics, Innovation and Management of the Java Sea Pelagic Fisheries. Seminar SOSEKIMA. (eds. Roch J., S. Nurhakim,.I. Widodo, and A. Poernomo) 63–80 (1995).
73	peer-reviewed	Watson, M. S., Claar, D. C. & Baum, J. K. Subsistence in isolation: Fishing dependence and perceptions of change on Kiritimati, the world's largest atoll. Ocean & coastal management 123, 1–8 (2016).
74	grey literature	FAO & SPC. Fishery and Aquaculture Economics and Policy Division. Report of the FAO/SPC Pacific Islands Regional Consultation on the Development of Guidelines for Securing Sustainable Small-Scale Fisheries, Noumea, New Caledonia, 12-14 June 2012. (2012).
75	peer-reviewed	Campbell, D. et al. Wild Food Harvest, Food Security, and Biodiversity Conservation in Jamaica: A Case Study of the Millbank Farming Region. Front. Sustain. Food Syst. 5, (2021).
76	peer-reviewed	Thomas, A. et al. Why they must be counted: Significant contributions of Fijian women fishers to food security and livelihoods. Ocean & Coastal Management 205, 105571 (2021).
84	peer-reviewed	Smith, D. The relational attributes of marketplaces in post-earthquake Port-au-Prince, Haiti. Environment and Urbanization 31, 497–516 (2019).
85	grey literature	Rut, M. Following sustainable food narratives in Singapore. SHARE CITY (2018).
99	grey literature	FAO. FAO and partners help restore nutrition and agricultural livelihoods in the Pacific Islands. Vanuatu. (2016).
100	grey literature	WFP. Decentralized Evaluation. Final evaluation of WFP Haiti's Food for Education and Child Nutrition Programme (2016-2019). (2019).
101	grey literature	WFP. Decentralized Evaluation. Final Evaluation of McGovern-Dole International Food for Education and Child Nutrition Program in Guinea-Bissau (2016-2019). (2021).
102	grey literature	Committee on World Food Security. Regional Initiatives. Pacific Food Summit. (2010).
103	grey literature	WFP. Strengthening National Safety Nets. School Feeding: WFP's Evolving Role in Latin America and the Caribbean.(2016).
104	grey literature	FAO. State of food insecurity in the CARICOM Caribbean. (2015).
105	grey literature	Thomas-Hope, E., Kinlocke, R., Ferguson, T., Heslop-Thomas, C. & Timmers, B. The Urban Food System of Kingston, Jamaica. (Hungry Cities Partnership, 2017).
106	grey literature	FAO. CARICOM Food Import Bill, Food Security and Nutrition. (2013).

107	grey literature	Hungry Cities Partnership. An Urban Perspective on Food Security in the Global South. (2018).
108	peer-reviewed	Emiliata, T. et al. Capturing the Experiences of Samoa: The Changing Food Environment and Food Security in Samoa during the COVID-19 Pandemic. Oceania 90, 116–125 (2020).
109	peer-reviewed	Narine, T. & Badrie, N. Influential Factors Affecting Food Choices of Consumers When Eating Outside the Household in Trinidad, West Indies. Journal of Food Products Marketing 13, 19–29 (2007).
110	peer-reviewed	Barr, S. Using Mixed Methods to Describe a Spatially Dynamic Food Environment in Rural Dominican Republic. Hum Ecol 45, 845–851 (2017).
111	grey literature	Thomas, G. Growing Greener Cities in Latin America and the Caribbean: An FAO Report on Urban and Peri-Urban Agriculture in the Region. (FAO, 2014).
112	peer-reviewed	Morshed, A. B., Becker, H. V., Delnatus, J. R., Wolff, P. B. & Iannotti, L. L. Early nutrition transition in Haiti: linking food purchasing and availability to overweight status in school-aged children. Public health nutrition 19, 3378–3385 (2016).
113	grey literature	Kinlocke, R. & Thomas-Hope, E. Inclusive growth and the informal food sector in Kingston, Jamaica. (Hungry Cities Partnership, 2020).
114	grey literature	WFP. Caribbean COVID-19 Food Security & Livelihoods Impact Survey - Barbados Summary Report. (2020).
115	grey literature	FAO. Enhancing evidence-based decision making for sustainable agriculture sector development in Pacific Islands Countries. (2010).
116	grey literature	FAO. Documentation of the traditional food system of Pohnpei. (2009).
117	grey literature	WFP. Caribbean COVID-19 Food Security & Livelihoods Impact Survey - Regional Summary Report. (2020).
118	peer-reviewed	Bottcher, C., Underhill, S. J. R., Aliakbari, J. & Burkhart, S. J. Food Access and Availability in Auki, Solomon Islands. Journal of Hunger & Environmental Nutrition 16, 751–769 (2021).
119	grey literature	WHO. Healthy marketplaces in the Western Pacific: guiding future action: applying a settings approach to the promotion of health in marketplaces. (2014).
120	grey literature	Gordons Market, Port Moresby officially opens. UN Women – Asia-Pacific (2019).
121	grey literature	Making markets safe for women vendors in Papua New Guinea. UN Women – Headquarters (2014).
122	grey literature	FAO. The Right to Food in the CARICOM Region: An Assessment Report. (2013).
123	peer-reviewed	Bottcher, C., Underhill, S. J., Aliakbari, J. & Burkhart, S. J. Food purchasing behaviors of a remote and rural adult Solomon islander population. Foods 8, 464 (2019).
124	grey literature	WFP. Caribbean COVID-19 Food Security & Livelihoods Impact Survey - Belize Summary Report. (2020).
125	peer-reviewed	Hongjun, W. Food and the Singapore young consumer. Young Consumers 7, 53–59 (2006).
126	peer-reviewed	Naidoo, N. et al. Determinants of eating at local and western fast-food venues in an urban Asian population: a mixed methods approach. Int J Behav Nutr Phys Act 14, 69 (2017).
127	grey literature	FAO. Mainstreaming Ecosystem Services and Biodiversity into Agricultural Production and Management in the Pacific Islands: Technical Guidance Document. (2016).
128	grey literature	Rosset, P. & Benjamin, M. Two Steps Back, One Step Forward: Cuba's National Policy for Alternative Agriculture. (International Institute for Environment and Development (IIED), Sustainable Agriculture Programme, London, 1994).
129	grey literature	FAO. FAO and SIDS: Challenges and Emerging Issues in Agriculture, Forestry and Fisheries: Paper Prepared by FAO on the Occasion of the Inter-Regional Conference of Small Island Developing States, Bahamas, 26-30 January 2004. (2004).
130	peer-reviewed	Thaman, R. R. Urban food gardening in the Pacific Islands: A basis for food security in rapidly urbanising small-island states. Habitat International 19, 209–224 (1995).
131	grey literature	Valstar, A. Home-based food production in urban Jamaica. (FAO, 1999).
132	grey literature	FAO. Pacific islands and FAO achievements and success stories. (2011).
133	grey literature	Hibi, E., Lam, F. & Chopin, F. Accelerating Action on Food Security and Nutrition in Pacific Small Island Developing States (SIDS). (FAO, 2018).
134	grey literature	Caribbean Agribusiness. Urban and Peri-urban Agriculture in Latin America and the Caribbean. Antigua and Barbuda. (2015).
135	grey literature	Thomas-Hope, E., Kinlocke, R. & Ferguson, T. Nr. 09: Enhancing food security through urban agriculture in Kingston, Jamaica. (Hungry Cities Partnership, 2020).
136	grey literature	WHO. Diet, food supply and obesity in the Pacific. (2003).
14	peer-reviewed	Craven, L. K. & Gartaula, H. N. Conceptualising the Migration–Food Security Nexus: Lessons from Nepal and Vanuatu. Australian Geographer 46, 455–471 (2015).
138	grey literature	WFP. Caribbean COVID-19 Food Security & Livelihoods Impact Survey -Trinidad and Tobago Summary Report. (2020).
139	grey literature	WFP. Caribbean COVID-19 Food Security & Livelihoods Impact Survey - Jamaica Summary Report. (2020).
140	grey literature	WFP. Caribbean COVID-19 Food Security & Livelihoods Impact Survey - Bahamas Summary Report. (2020).
141	grey literature	WFP. Caribbean COVID-19 Food Security & Livelihoods Impact Survey - Grenada Summary Report. (2020).
142	grey literature	WFP. Caribbean COVID-19 Food Security & Livelihoods Impact Survey - British Virgin Islands Summary Report. (2020).
143	peer-reviewed	Garcia-Montiel, D. C. et al. Food sources and accessibility and waste disposal patterns across an urban tropical watershed: implications for the flow of materials and energy. Ecology and Society 19, (2014).
144	peer-reviewed	Lucantoni, D. Transition to agroecology for improved food security and better living conditions: case study from a family farm in Pinar del Río, Cuba. Agroecology and Sustainable Food Systems 44, 1124–1161 (2020).
145	grey literature	Iimi, A. Hidden Treasures in the Comoros: The Impact of Inter-Island Connectivity Improvement on Agricultural Production. (The World Bank, 2019).

146	grey literature	FAO. Food Security and Nutrition in Small Island Developing States (SIDS). (2014).
147	grey literature	FAO. Improving the Capacity of Farmers to Market a Consistent Supply of Safe, Quality Food - TCP/SAM/3601. (2020).
148	grey literature	FAO. Agriculture for Growth: learning from experience in the Pacific. (2010).
149	peer-reviewed	Rodríguez, D. I., Anríquez, G. & Riveros, J. L. Food security and livestock: The case of Latin America and the Caribbean. Ciencia e investigación agraria 43, 5–15 (2016).
150	grey literature	WFP. Caribbean COVID-19 Food Security & Livelihoods Impact Survey - St. Kitts and Nevis Summary Report. (2020).
151	grey literature	FAO. Global Blue Growth Initiative and Small Island Developing States (SIDS). (2014).
152	grey literature	Sherzad, S. Family Farming in the Pacific Islands Countries: Challenges and Opportunities. (FAO, 2018).
153	grey literature	Beyer, R. & Stice, K. FAO Roundtable on the competitiveness of Pacific Island Small and Medium agro-processing enterprises, 11-13 April 2012, Nadi, Fiji Islands. (FAO, 2012).
154	peer-reviewed	Bryceson, K. P. & Ross, A. Agrifood chains as complex systems and the role of informality in their sustainability in small scale societies. Sustainability 12, 6535 (2020).
155	grey literature	Dean, G., Lyons, G. & Edis, R. How food gardens based on traditional practice can improve health in the Pacific. The Conversation (2017).
156	peer-reviewed	Opio, F. Contribution of subsistence diets to farm household nutrient requirements in the pacific. Ecology of Food and Nutrition 29, 285–305 (1993).
157	grey literature	FAO. Strengthened Household Agroforestry and Food Production in Nauru - TCP/NAU/3501. (FAO, 2019).
158	grey literature	IFAD. Investing in rural people in Papua New Guinea. (2020).
159	grey literature	WFP. Caribbean COVID-19 Food Security & Livelihoods Impact Survey - Guyana Summary Report. (2020).
160	grey literature	IFAD. Investing in rural people in Guinea-Bissau. (2019).
161	peer-reviewed	Chaplowe, S. G. Havana’s popular gardens:sustainable prospects for urban agriculture. The Environmentalist 18, 47–57 (1998).
162	peer-reviewed	McIlvaine-Newsad, H., Porter, R. & Delany-Barmann, G. Change the game, not the rules: The role of community gardens in disaster resilience. Journal of Park and Recreation Administration 38, 2–22 (2020).
163	grey literature	Grieg-Gran, M., Guijt, I. & Peutalo, B. Local Perspectives on Forest Values in Papua New Guinea - The Scope for Participatory Methods. (International Institute for Environment and Development, 2002).
164	peer-reviewed	Altieri, M. A. et al. The greening of the “barrios”: Urban agriculture for food security in Cuba. Agriculture and Human Values 16, 131–140 (1999).
165	grey literature	RUAF. Enhancing the Contribution of Urban Agriculture to Food Security. Urban Agriculture Magazine Special Issue (2002).
166	grey literature	García-Quijano, C. G. & Lloréns, H. What rural, coastal Puerto Ricans can teach us about thriving in times of crisis. The Conversation (2017).
167	grey literature	Davies et al. Singapore SHARECITY Profile. (SHARE CITY, 2017).
168	grey literature	FAO & Government of Samoa. Food security and food consumption in Samoa: Based on the analysis of the 2018 household income and expenditure survey. (2020).
169	grey literature	FAO & SPC. Solomon Islands Food Security Profile. (2020).
170	grey literature	FAO. Tonga: Food Security Profile. (2019).
171	grey literature	KNSO, FAO & SPC. Kiribati Food Security Profile. (2021).
172	grey literature	FAO, Pacific Community & Tuvalu Central Statistics Division. Tuvalu Food Security Profile. (2022).
173	grey literature	Tora, T. Two piglets for a kayak: Fiji returns to barter system as Covid-19 hits economy. The Guardian (2020).
174	grey literature	Siutaia, H. Le Barter trading platform glimpse of the past. Samoa Observer (2020).
175	grey literature	Maeir AM. A Feast in Papua New Guinea. Near Eastern Archaeology 78, 26-34 (The University of Chicago Press, 2015).
176	peer-reviewed	Erskine, W. et al. The role of wild foods in food security: the example of Timor-Leste. Food Sec. 7, 55–65 (2015).
177	grey literature	FAO. Natural Resources Management and the Environment in Small Island Developing States (SIDS). (2014).
178	grey literature	FAO. Forest and Forestry in Small Island Developing States. (2002).
179	grey literature	FAO. Disaster risk management and climate change adaptation in the CARICOM and wider Caribbean region. Strategy and action plan. (2015).
180	grey literature	FAO. Management of large pelagic fisheries in CARICOM countries. (2004).
181	grey literature	Gillett, R., Moy, W., & Fishery and Aquaculture Economics and Policy Division. Spearfishing in the Pacific Islands. Current Status and Management Issues. (FAO, 2006).
