# Supplementary material for: Examining Food Sources and Their Interconnections over Time in Small Island Developing States: A Systematic Scoping Review
Source: Nutrients. 2025 Jul 18;17(14):2353. doi: 10.3390/nu17142353 (PMC12298424; doi:10.3390/nu17142353)
Supplement: Supplementary file 1 [file nutrients-17-02353-s001.zip › PsycINFO database_search strategy.pdf]

Search strategy: APA PsycINFO database

https://libguides.cam.ac.uk/az.php?a=m via Raven

310 references retrieved on 18 June 2021 (no limits applied)

Combination of Keywords and Subject Heading.

- Boolean/Phrase – Supports any Boolean searching or exact phrase searching. Stop words are ignored when part of phrases being searched.
- Title and abstract
- Thesaurus terms: DE "word"

A) Food Sources = a or b or c or d

a) General

DE "food preferences" or DE "eating behavior"

TI ((foodscape or "food environment" or "food desert" or "food swamp" or "obesogenic environment" or "nutrition* environment" or "food forest" or "food sourc*" or "market-based food" or "marketbased food" or "food purchas*" or "dietary pattern" or "dietary behavio#r" or "dietary behavio#rs" or "food consumption pattern" or "food consumption behavio#r" or "food consumption behavio#rs" or "food acqui*" or "food choice" or "food preference")) OR **AB** ((foodscape or "food environment" or "food desert" or "food swamp" or "obesogenic environment" or "nutrition* environment" or "food forest" or "food sourc*" or "market-based food" or "marketbased food" or "food purchas*" or "dietary pattern" or "dietary behavio#r" or "dietary behavio#rs" or "food consumption pattern" or "food consumption behavio#r" or "food consumption behavio#rs" or "food acqui*" or "food choice" or "food preference"))

b) Own production (1 or 2 or 3)

1. TI (((commun* OR urban* OR rural* OR local* OR school OR work OR workpl* OR smallhold* OR small hold*) N3 (allotment* or agricult* OR horticult* OR garden* OR farm* OR agroprocessing OR "agro processing" OR aquacultur* OR fishing OR fisheries OR fishery OR maricult* OR "food production"))) OR AB (((commun* OR urban* OR rural* OR local* OR school OR work OR workpl* OR smallhold* OR small hold*) N3 (allotment* or agricult* OR horticult* OR garden* OR farm* OR agroprocessing OR "agro processing" OR aquacultur* OR fishing OR fisheries OR fishery OR maricult* OR "food production")))

2. TI (((food OR animal OR fruit OR vegetable OR produce OR greens OR crop* OR insect OR bees OR bird OR nuts or plant or honey) N3 ("own produc*" OR rear* OR forag* OR gather* OR harvest* OR hunt*))) OR AB (((food OR animal OR fruit OR vegetable OR produce OR greens OR crop* OR insect OR bees OR bird OR nuts or plant or honey) N3 ("own produc*" OR rear* OR forag* OR gather* OR harvest* OR hunt*)))

3. TI ((Wild) N1 (food or plant)) OR AB ((Wild) N1 (food or plant))

c) Purchase

TI (((enterprise OR trading OR trader OR dealer OR retailer OR entrepreneur OR vendor OR street OR school OR college OR hawker or umbrella or stall or pallet or shop or kiosk or store or market or parlour or grocer* or truck or van or pick-up or pickup or trike or bicycle or bike or tricycle or wholesale or bulk or distributor or takeaway or take-away or takeout or take-out or fast) N1 (food or beverage or fruit or vegetable or meal or snack))) OR AB (((enterprise OR trading OR trader OR dealer OR retailer OR entrepreneur OR vendor OR street OR school OR college OR hawker or umbrella or stall or pallet or shop or kiosk or store or market or parlour or grocer* or truck or van or

pick-up or pickup or trike or bicycle or bike or tricycle or wholesale or bulk or distributor or takeaway or take-away or takeout or take-out or fast) N1 (food or beverage or fruit or vegetable or meal or snack)))

d) *Food exchange and Food Aid (4 or 5 or 6)*

4. TI ((food OR beverage OR meal OR fruit OR vegetable) N1 (transfer* OR borrow* OR exchang* OR barter* OR shar* OR aid* OR gift* OR bank* OR parcel* OR "faith-based organisation" OR "shipp* barrel"))) OR AB ((food OR beverage OR meal OR fruit OR vegetable) N1 (transfer* OR borrow* OR exchang* OR barter* OR shar* OR aid* OR gift* OR bank* OR parcel* OR "faith-based organisation" OR "shipp* barrel")))

5. TI ((tanda OR "partner hand" OR partnerhand OR "box hand" OR boxhand OR ROSCAs OR rosca OR "food program*" OR "food kitchen" OR "food sharing initiative" OR "food network" OR sou-sou OR susu OR asue OR feasting)) OR AB ((tanda OR "partner hand" OR partnerhand OR "box hand" OR boxhand OR ROSCAs OR rosca OR "food program*" OR "food kitchen" OR "food sharing initiative" OR "food network" OR sou-sou OR susu OR asue OR feasting))

6. TI (((commun* OR cultur* OR religio*) N1 (feast*))) OR AB (((commun* OR cultur* OR religio*) N1 (feast*)))

B) Small Island Developing States

DE "Caribbean" OR DE "Caribbean Community" OR DE "Micronesia" OR DE "Melanesia" OR DE "Polynesia"

TI (("Small Island* Developing State" or SIDS or Anguilla* OR Caribbean OR Antigua* OR Antilles* OR Aruba* OR Bahamas* OR Barbuda* OR Barbados* OR Belize* OR Bermuda* OR Caicos* OR Caledonia* OR Cayman* OR Comoros* OR "Cook Island*" OR Cuba* OR Curacao* OR Dominica* OR Dominican* OR Fiji* OR Grenada* OR Grenadines* OR Guadeloupe* OR Guam* OR Guinea-Bissau* OR Haiti* OR Jamaica* OR Kiribati* OR Lucia* OR Maarten* OR Maldives* OR Marshall* OR Martinique* OR Mauritius* OR Melanesia* OR Micronesia* OR Montserrat* OR Nauru* OR Nevis* OR Niue* OR Palau* OR Papua* OR Polynesia* OR Principe* OR Kitts* OR Samoa* OR "Sao Tome*" OR Seychelles* OR Singapore* OR Solomon* OR Suriname* OR Timor-Leste* OR Tonga* OR Trinidad* OR Tobago* OR Tokelau* OR Turks* OR Tuvalu* OR "Puerto Rico*" OR Marianas* OR Martinique* OR Vanuatu* OR Verde* OR Vincent* OR "Virgin Island*")) OR AB (("Small Island* Developing State" or SIDS or Anguilla* OR Caribbean OR Antigua* OR Antilles* OR Aruba* OR Bahamas* OR Barbuda* OR Barbados* OR Belize* OR Bermuda* OR Caicos* OR Caledonia* OR Cayman* OR Comoros* OR "Cook Island*" OR Cuba* OR Curacao* OR Dominica* OR Dominican* OR Fiji* OR Grenada* OR Grenadines* OR Guadeloupe* OR Guam* OR Guinea-Bissau* OR Haiti* OR Jamaica* OR Kiribati* OR Lucia* OR Maarten* OR Maldives* OR Marshall* OR Martinique* OR Mauritius* OR Melanesia* OR Micronesia* OR Montserrat* OR Nauru* OR Nevis* OR Niue* OR Palau* OR Papua* OR Polynesia* OR Principe* OR Kitts* OR Samoa* OR "Sao Tome*" OR Seychelles* OR Singapore* OR Solomon* OR Suriname* OR Timor-Leste* OR Tonga* OR Trinidad* OR Tobago* OR Tokelau* OR Turks* OR Tuvalu* OR "Puerto Rico*" OR Marianas* OR Martinique* OR Vanuatu* OR Verde* OR Vincent* OR "Virgin Island*"))

Full search:
(A) AND (B)
