# Supplementary material for: Examining Food Sources and Their Interconnections over Time in Small Island Developing States: A Systematic Scoping Review
Source: Nutrients. 2025 Jul 18;17(14):2353. doi: 10.3390/nu17142353 (PMC12298424; doi:10.3390/nu17142353)
Supplement: Supplementary file 1 [file nutrients-17-02353-s001.zip › PubAg database_search strategy.pdf]

Search strategy: PubAg Database

https://pubag.nal.usda.gov/advanced

183 hits retrieved on 17 June 2021 (no limits applied)

Combination of Keywords and **Subjects**.

- Advanced Search, All Fields
- Truncation and wildcards are not supported
- Word-stemming is done automatically

(((((((**foodways** or **feeding behavior** or **feeding preferences** or foodscape or "food environment" or "food desert" or "food swamp" or "obesogenic environment" or "nutrition environment" or "food forest" or "food sourc" or "market-based food" or "marketbased food" or "food purchas" or "dietary pattern" or "dietary behavio" or "food consumption pattern" or "food consumption behavio" or "food acqui" or "food choice" or "food preference") OR ((commun or urban OR rural OR local OR school OR work OR workpl OR smallhold OR "small hold") AND (allotment OR agricult OR horticult OR garden OR farm OR agroprocessing OR "agro processing" OR aquacultur OR fishing OR fisheries OR fishery OR maricult OR "food production"))) OR ((food OR animal OR fruit OR vegetable OR produce OR greens OR crop OR insect OR bees OR bird OR nuts or plant or honey) AND ("own produc" OR rear OR forag OR gather OR harvest OR hunt or wild))) OR ((enterprise OR trading OR trader OR dealer OR retailer OR entrepreneur OR vendor OR street OR school OR college OR hawker or umbrella or stall or pallet or shop or kiosk or store or market or parlour or grocer or truck or van or pick-up or pickup or trike or bicycle or bike or tricycle or wholesale or bulk or distributor or takeaway or take-away or takeout or take-out or fast) + (food or beverag or fruit or vegetable or meal or snack))) OR ((food OR beverag OR meal OR fruit OR vegetable) AND (transfer OR borrow OR exchang OR barter OR shar OR aid OR gift OR bank OR parcel OR "faith-based organisation" OR "shipp barrel"))) OR (tanda OR partner-hand OR partnerhand OR box-hand OR boxhand OR ROSCAs OR rosca OR "food program" OR "food kitchen" OR "food sharing initiative" OR "food network" OR sou-sou OR susu OR asue OR feasting)) OR ((commun OR cultur OR religio) AND (feast))) AND (**Caribbean** OR Melanesia OR Micronesia or "Commonwealth of the Northern Mariana Islands" or "Small Island Developing State" or SIDS or Anguilla* OR Antigua OR Antilles OR Aruba OR Bahamas OR Barbuda OR Barbados OR Belize OR Bermuda OR Caicos OR Caledonia OR Cayman OR Comoros OR "Cook Island" OR Cuba OR Curacao OR Dominica OR Dominican OR Fiji OR Grenada OR Grenadines OR Guadeloupe OR Guam OR Guinea-Bissau OR Haiti OR Jamaica OR Kiribati OR Lucia OR Maarten* OR Maldives OR Marshall OR Martinique OR Mauritius OR Montserrat OR Nauru OR Nevis OR Niue OR Palau OR Papua OR Polynesia OR Principe OR Kitts OR Samoa OR "Sao Tome" OR Seychelles OR Singapore OR Solomon OR Suriname OR Timor-Leste OR Tonga OR Trinidad OR Tobago OR Tokelau OR Turks OR Tuvalu OR "Puerto Rico" OR Marianas OR Martinique OR Vanuatu OR Verde OR Vincent OR "Virgin Island"))
