# Supplementary material for: Examining Food Sources and Their Interconnections over Time in Small Island Developing States: A Systematic Scoping Review
Source: Nutrients. 2025 Jul 18;17(14):2353. doi: 10.3390/nu17142353 (PMC12298424; doi:10.3390/nu17142353)
Supplement: Supplementary file 1 [file nutrients-17-02353-s001.zip › S1. Eligibility Criteria_initial.pdf]

Eligibility Criteria

Time Frame

All studies will be eligible regardless of their publication date. The choice of not applying a time frame is pragmatic and it will be reviewed following initial searches and adjustment, if necessary.

Geographical scope

The geographical scope of this review will focus on SIDS only. The list of eligible SIDS has been developed following previous authors criteria. This includes SIDS listed by the United Nations, with the addition of the Caribbean Region and Melanesia as Mesh terms, and Tokelau as country.

Languages

Restrictions in language will not be made although the search terms and the primarily language of the sources of data used will be in English. In subsequent work following this review, we will consider the addition of Spanish and French in keywords and sources of data.

Sources of food

The unit of analysis or intervention of this review are the food sources. Therefore, studies reporting food-related issues, such as food transport and distribution or dietary and consumption patterns not linked to a food source will be excluded from this review. Similarly, food sources for non-human feeding or food practices (e.g., hunting) by non-humans, and food sources (such as large commercial farms) not described as part of the food environment (i.e., there is no link or interaction with SIDS population) will also be excluded from this review.

Study and document type

All study designs and document types will be included (e.g., research articles, book chapters or conference proceedings) as long as sufficient information is provided to allow for data extraction. Studies reporting hypothetical food sources or hypothetical scenarios will be excluded.

Information Sources

Due to the nature of the topic, we anticipate there will be challenges identifying all relevant evidence. These challenges can be the result of 1. including informal food sources which might not be registered by official bodies thus may lack datasets containing information, hence not being considered in research studies, 2. including non-market-based food sources such as food sharing initiatives which might be reported through non-peer-reviewed sources or more informal channels, 3. a combination of both. In consequence, a combination of published and unpublished literature will be included in this review using a variety of sources of data (Table 2). The choice of databases has been informed by previous authors and aims to cover a range of disciplines according to objective 3 of this review. The choice of UN repositories has been informed by a training course provided by WHO Library and in consultation with a WHO librarian. The final list of data sources will be refined following an iterative process.

Table 2. Sources of data and brief description of each.

Source Type	Description	Details
Peer-reviewed databases	Health	MEDLINE/EMBASE; CINAHL, PsycINFO, Global Health

	Economic & Social Sciences	Web of science/SCOPUS, ASSIA, Econlit
	Agricultural Science	AGRICOLA/PubAg, FSTA
	Site-specific	WPRIM, LILACS/PAHO, MedCarib, SciELO
Grey Literature	Repositories of UN Agencies	IRIS (WHO publication repository), PAHO IRIS, FAO Repository, AGRIS, WFP, IFAD, UNICEF, UNOPS, UN OHRLLS
	Websites of relevant International Networks	Hungry Cities Partnership, Resource Centres on Urban Agriculture and Food Security (RUAF), Emergency Nutrition Network (ENN), International Institute for Environment and Development (IIED), ShareCity database, Caribbean Agribusiness, The Pacific Community (SPC), The Caribbean Public Health Agency (CARPHA), The Conversation.
Snowball	References from key included studies will be used to find other relevant titles	

Database/Website	Characteristics
MEDLINE	Indexes over 5,500 biomedical journals; coverage is worldwide, but (86%) of the records are from English-language resources. Scope covers medicine, nursing, dentistry, veterinary medicine, the health-care system and the pre- clinical sciences as well as microbiology, delivery of health care, nutrition, pharmacology and environmental health. Database updated weekly.
EMBASE	Very complementary to Medline with strengths in pharmacology, drug research & toxicology. Indexes over 8,500 biomedical journals from 1947 to the present from 95 countries.
CINAHL	Indexes 5,500 journals; English-language and selected other-language journal articles. Scope covers nursing, biomedicine, health sciences librarianship, alternative/complementary medicine, consumer health and 17 allied health disciplines. It includes nursing journals and publications from the National League for Nursing and the American Nurses Association.
PsycINFO	Over 2,500 peer-reviewed journal titles (78% of the overall content). It covers the field of psychology. Interdisciplinary aspects of behavioural and social science research. It contains journal articles, book chapters, books, and dissertations. Publications from at least 50 countries, with journals in more than 27 languages, and non-English titles in Roman alphabets from 1978 to the present.
Global Health	Dedicated to public health, with a global perspective. Over 4,000 peer-reviewed journals of which more than 3,600 are unique to Global Health
Web of Science	Access to multiple databases of many different academic disciplines. Coverage from the year 1900 to the present. Over 12,000 high impact journals and 160,000 conference proceedings. Titles of foreign-language publications are translated into English and so cannot be found by searches in the original language.
SCOPUS	From over 11,500 publishers. It covers three types of sources: book series, journals, and trade journals. Conference proceedings included. Scope covers the fields of science, technology, medicine, social sciences, and arts and humanities.

ASSIA	From over 500 journals published in 19 different countries. Applied Social Sciences Index and Abstracts is an indexing and abstracting tool covering health, social services, psychology, sociology, economics, politics, social problems such as poverty, race relations and education. Social science and health information for the practical and academic professional.
EconLit	It covers economics literature published over the last 130 years from leading institutions in 74 countries. Working papers, books, dissertations, articles and peer-reviewed articles.
AGRICOLA	It serves as the public catalog of the National Agricultural Library. It contains peer-reviewed journal articles, as well as monographs, serials, audiovisual materials and online content from around the world. Scope covers agriculture and its allied fields.
PubAg	Some overlap with AGRICOLA. It focuses on full-text publications from USDA scientists. It contains full-text articles relevant to the agricultural sciences, along with citations to peer-reviewed journal articles. Designed for farmers, scientists, scholars, students, and the general public.
FSTA (no access)	Food Science and Technology Abstracts. specialized database covering scientific and technological literature relating to food, beverages and nutrition.
WPRIM	Medical/Health database from Western Pacific Region
LILACS	Medical/Health database from Latin America and Caribbean Region
MedCarib	Medicine/Health database from English speaking Caribbean and Suriname
SciELO	Created to meet the scientific communication needs of developing countries. 16 countries: Argentina, Bolivia, Brazil, Chile, Colombia, Costa Rica, Cuba, Ecuador, Mexico, Paraguay, Peru, Portugal, South Africa, Spain, Uruguay, and Venezuela. Over 1,000 journals
IRIS (WHO Repository)	WHO documents and publications from HQ and Regional Offices, WHO periodicals, articles outside periodicals on WHO work, governing bodies documents, press and audiovisual materials, historical materials. Contains WHOLIS database
PAHO Regional Database	Not included in IRIS. Locally produced information. South – South information sharing
FAO Repository	All indexed publications as well as official reports and other papers and documents published by FAO, as well as some materials produced by other sources with FAO involvement.
AGRIS	Supported by FAO. Food and agricultural scientific research with special attention to scientific information produced in the global south.
WPF Publications	All indexed publications, annual reports and other papers and documents published by WPF
IFAD	UN agency focusing on poverty and hunger in rural areas of developing countries. All publications, reports, other papers and documents
UNICEF	Publications using household surveys, annual reports and stories organised by topic including child health and child nutrition.
UNOPS	a United Nations agency dedicated to implementing infrastructure and procurement projects for the United Nations System, international financial institutions, governments and other partners around the world.
UN-OHRLLS	The United Nations Office of the High Representative for the Least Developed Countries, Landlocked Developing Countries and Small Island Developing States
Hungry Cities Partnership	International network of cities and city-based partner organizations which focuses on the relationships between rapid urbanization, informality, inclusive growth and urban food systems in the Global South. Reports, papers, book chapters, articles, presentations, briefs, thesis. Mainly for Jamaica.
RUAF	Global partnership on sustainable urban agriculture and food systems that aims to transform urban food systems in cities around the world.

ENN	UK registered charity but works globally on know-how and evidence to overcome malnutrition.
IIED	Over 7,000 publications carried out by IIED, mostly available to download free of charge. It ranges from briefings, to case studies to reports. Includes publication from Environment & Urbanization journal.
ShareCity database	A collaborative and trans-disciplinary approach to assess the practice and sustainability potential of city-based food sharing economies. Publications, presentations, blog articles. Project of Trinity College Dublin, funded by EU Horizon 2020.
Caribbean Agribusiness	A one-stop website containing information on food sources, trade, food security, etc. organised by country and topics.
The Pacific Community (SPC)	A central repository, online cataloguing and dissemination system of Pacific Island statistical microdata, metadata, reports and documents.
The Caribbean Public Health Agency (CARPHA)	Publication catalogue including annual reports, manuals, guidelines, newsletters, and NCDs and nutrition-related publications.
The Conversation	A network of not-for-profit media outlets publishing news stories and research reports online, with accompanying expert opinion and analysis. Articles are written by academics and researchers under a Creative Commons license, allowing reuse without modification.
