# Supplementary material for: Examining Food Sources and Their Interconnections over Time in Small Island Developing States: A Systematic Scoping Review
Source: Nutrients. 2025 Jul 18;17(14):2353. doi: 10.3390/nu17142353 (PMC12298424; doi:10.3390/nu17142353)
Supplement: Supplementary file 1 [file nutrients-17-02353-s001.zip › SCOPUS database_search strategy.pdf]

Search strategy: SCOPUS database

Signed in with Institutional Account
https://libguides.cam.ac.uk/az.php?a=m via Raven

SCOPUS:
3682 references retrieved on 28 June 2021 (no limits applied)

Search terms only. Subject Heading not available.

- Default page Document search
- Search within Article title, Abstract, Keywords

A) Food Sources = a or b or c or d

a) General

TITLE-ABS-KEY(foodscape or "food environment" or "food desert" or "food swamp" or "obesogenic environment" or "nutrition* environment" or "food forest" or "food sourc*" or "market-based food" or "marketbased food" or "food purchas*" or "dietary pattern" or "dietary behavior" or "food consumption pattern" or "food consumption behavior" or "food acqui*" or "food choice" or "food preference")

b) Own production (1 or 2)

1. TITLE-ABS-KEY(((commun* OR urban* OR rural* OR local* OR school* OR work* OR workpl* OR smallhold* OR "small hold*") W/1 (allotment or agricult* OR horticult* OR garden* OR farm* OR agroprocessing OR "agro processing" OR aquacultur* OR fishing OR fishery OR maricult* OR "food production")))

2. TITLE-ABS-KEY((food OR animal OR fruit OR vegetable OR produce OR greens OR crop* OR insect OR bees OR bird OR nuts OR plant or honey) W/1 ("own produc*" OR rear* OR forag* OR gather* OR harvest* OR hunt*))

3. TITLE-ABS-KEY(Wild*) W/1 (food or plant)

c) Purchase

TITLE-ABS-KEY((enterprise OR trading OR trader OR dealer OR retailer OR entrepreneur OR vendor OR street OR school OR college OR hawker or umbrella or stall or pallet or shop or kiosk or store or market or parlour or grocery or truck or van or pick-up or pickup or trike or bicycle or bike or tricycle or wholesale or bulk or distributor or takeaway or take-away or takeout or take-out or fast or abattoir or slaughterhouse or butcher or bar or tavern or restaurant) W/1 (food or beverage or fruit or vegetable or meal or snack))

d) Food exchange and Food Aid (3 or 4 or 5)

3. TITLE-ABS-KEY((food OR beverage OR meal OR fruit OR vegetable) W/1 (transfer* OR borrow* OR exchang* OR barter* OR shar* OR aid* OR gift* OR bank* OR parcel* OR "faith-based organisation*" OR "shipp* barrel*"))

4. TITLE-ABS-KEY(tanda OR "partner hand" OR partnerhand OR "box hand" OR boxhand OR ROSCAs OR rosca OR "food program*" OR "food kitchen" OR "food sharing initiative" OR "food network*" OR sou-sou OR susu OR asue OR feasting)

5. TITLE-ABS-KEY((commun* OR cultur* OR religio*) W/1 (feast*))

B) Small Island Developing States

TITLE-ABS-KEY(Caribbean or Melanesia or Micronesia or "Commonwealth of the Northern Mariana Islands" or "Small Island Developing State" or SIDS or Anguilla OR Antigua OR Antilles OR Aruba OR Bahamas OR Barbuda OR Barbados OR Belize OR Bermuda OR Caicos OR Caledonia OR Cayman OR Comoros OR "Cook Island" OR Cuba OR Curacao OR Dominica OR Dominican OR Fiji OR Grenada OR Grenadines OR Guadeloupe OR Guam OR Guinea-Bissau OR Haiti OR Jamaica OR Kiribati OR Lucia OR Maarten OR Maldives OR Marshall OR Martinique OR Mauritius OR Montserrat OR Nauru OR Nevis OR Niue OR Palau OR Papua OR Polynesia OR Principe OR Kitts OR Samoa OR "Sao Tome" OR Seychelles OR Singapore OR Solomon OR Suriname OR Timor-Leste OR Tonga OR Trinidad OR Tobago OR Tokelau OR Turks OR Tuvalu OR "Puerto Rico" OR Marianas OR Martinique OR Vanuatu OR Verde OR Vincent OR "Virgin Island")

Full search:
(A) AND (B)

((TITLE-ABS-KEY (Caribbean or Melanesia or Micronesia or “Commonwealth of the Northern Mariana Islands” or “Small Island Developing State” or SIDS or Anguilla OR Antigua OR Antilles OR Aruba OR Bahamas OR Bahrain OR Barbuda OR Barbados OR Belize OR Bermuda OR Caicos OR Caledonia OR Cayman OR Comoros OR “Cook Island” OR Cuba OR Curacao OR Dominica OR Dominican OR Fiji OR Grenada OR Grenadines OR Guadeloupe OR Guam OR Guinea-Bissau OR Haiti OR Jamaica OR Kiribati OR Lucia OR Maarten OR Maldives OR Marshall OR Martinique OR Mauritius OR Montserrat OR Nauru OR Nevis OR Niue OR Palau OR Papua OR Polynesia OR Principe OR Kitts OR Samoa OR “Sao Tome” OR Seychelles OR Singapore OR Solomon OR Suriname OR Timor-Leste OR Tonga OR Trinidad OR Tobago OR Tokelau OR Turks OR Tuvalu OR “Puerto Rico” OR Marianas OR Martinique OR Vanuatu OR Verde OR Vincent OR “Virgin Island”)) AND (((TITLE-ABS-KEY(foodscape or "food environment" or "food desert" or "food swamp" or "obesogenic environment" or "nutrition* environment" or "food forest" or "food sourc*" or "market-based food" or "marketbased food" or "food purchas*" or "dietary pattern" or "dietary behavior" or "food consumption pattern" or "food consumption behavior" or "food acqui*" or "food choice" or "food preference")) OR (TITLE-ABS-KEY(((commun* OR urban* OR rural* OR local* OR school* OR work* OR workpl* OR smallhold* OR "small hold*") W/1 (allotment or agricult* OR horticult* OR garden* OR farm* OR agroprocessing OR "agro processing" OR aquacultur* OR fishing OR fishery OR maricult* OR "food production")))) OR (TITLE-ABS-KEY((food OR animal OR fruit OR vegetable OR produce OR greens OR crop* OR insect OR bees OR bird OR nuts OR plant or honey) W/1 ("own produc*" OR rear* OR forag* OR gather* OR harvest* OR hunt*))) OR (TITLE-ABS-KEY(Wild*) W/1 (food or plant)) OR (TITLE-ABS-KEY((enterprise OR trading OR trader OR dealer OR retailer OR entrepreneur OR vendor OR street OR school OR college OR hawker or umbrella or stall or pallet or shop or kiosk or store or market or parlour or grocery or truck or van or pick-up or pickup or trike or bicycle or bike or tricycle or wholesale or bulk or distributor or takeaway or take-away or takeout or take-out or fast or abattoir or slaughterhouse or butcher or bar or tavern or restaurant) W/1 (food or beverage or fruit or vegetable or meal or snack))) OR (TITLE-ABS-KEY((food OR beverage OR meal OR fruit OR vegetable) W/1 (transfer* OR borrow* OR exchang* OR barter* OR shar* OR aid* OR gift* OR bank* OR parcel* OR "faith-based organisation*" OR "shipp* barrel*"))) OR (TITLE-ABS-KEY(tanda OR "partner hand" OR partnerhand OR "box hand" OR boxhand OR ROSCAs OR rosca OR "food program*" OR "food kitchen" OR "food sharing initiative" OR "food network*" OR sou-sou OR susu OR asue OR feasting)) OR (TITLE-ABS-KEY((commun* OR cultur* OR religio*) W/1 (feast*))))))
