# Supplementary material for: Examining Food Sources and Their Interconnections over Time in Small Island Developing States: A Systematic Scoping Review
Source: Nutrients. 2025 Jul 18;17(14):2353. doi: 10.3390/nu17142353 (PMC12298424; doi:10.3390/nu17142353)
Supplement: Supplementary file 1 [file nutrients-17-02353-s001.zip › Web of Science database_search strategy.pdf]

Search strategy: Web of Science database
Core Collection – All Years, All Citation Indexes
https://libguides.cam.ac.uk/az.php?a=m via Raven

3388 hits retrieved on 28 June 2021 (no limits applied)

- Topic Field (to search Title, Abstract, Author Keywords and Keywords Plus® within a record)

A) Food Sources = a or b or c or d

a) General
foodscape$ or "food environment$" or "food desert$" or "food swamp$" or "obesogenic environment$" or "nutrition* environment$" or "food forest$" or "food sourc*" or "market-based food$" or "marketbased food$" or "food purchas*" or "dietary pattern$" or "dietary behavior$" or "dietary behaviour$" or "food consumption pattern$" or "food consumption behavior$" or "food consumption behaviour$"or "food acqui*" or "food choice$" or "food preference$"

b) Own production (1 or 2 or 3)
1. ((commun* OR urban* OR rural* OR local* OR school* OR work* OR workpl* OR smallhold* OR "small hold*") **NEAR/1** (allotment$ or agricult* OR horticult* OR garden* OR farm* OR agroprocessing OR "agro processing" OR aquacultur* OR fishing OR fisheries OR fishery OR maricult* OR "food production"))

2. ((food$ OR animal$ OR fruit$ OR vegetable$ OR produce OR greens OR crop* OR insect$ OR bees OR bird$ OR nuts or plant* or honey) **NEAR/1** ("own produc*" OR rear* OR forag* OR gather* OR harvest* OR hunt*))

3. (Wild*) NEAR/1 (food$ or plant*)

c) Purchase
((enterprise$ OR trading$ OR trader$ OR dealer$ OR retailer$ OR entrepreneur$ OR vendor$ OR street$ OR school$ OR college$ OR hawker$ or umbrella$ or stall$ or pallet$ or shop$ or kiosk$ or store$ or market$ or parlour$ or grocer* or truck$ or van$ or pick-up$ or pickup$ or trike$ or bicycle$ or bike$ or tricycle$ or wholesale$ or bulk$ or distributor$ or takeaway$ or take-away$ or takeout$ or take-out$ or fast) **NEAR/1** (food$ or beverage$ or fruit$ or vegetable$ or meal$ or snack$))

d) Food exchange and Food Aid (4 or 5 or 6)
4. ((food$ OR beverage$ OR meal$ OR fruit$ OR vegetable$) **NEAR/1** (transfer* OR borrow* OR exchang* OR barter* OR shar* OR aid* OR gift* OR bank* OR parcel* OR "faith-based organisation$" OR "shipp* barrel$"))

5. (tanda$ OR "partner hand" OR partnerhand OR "box hand" OR boxhand OR ROSCAs OR rosca OR "food program$" OR "food kitchen$" OR "food sharing initiative$" or "food network$" OR sou-sou OR susu OR asue OR feasting)

6. ((commun* OR cultur* OR religio*) **NEAR/1** (feast*))

B) Small Island Developing States

Caribbean or Melanesia or Micronesia or "Commonwealth of the Northern Mariana Islands" or "Small Island Developing State$" or SIDS or Anguilla OR Antigua OR Antilles OR Aruba OR Bahamas OR Bahrain OR Barbuda OR Barbados OR Belize OR Bermuda OR Caicos OR Caledonia OR Cayman OR Comoros OR "Cook Island$" OR Cuba OR Curacao OR Dominica OR Dominican OR Fiji OR Grenada OR

Grenadines OR Guadeloupe OR Guam OR Guinea-Bissau OR Haiti OR Jamaica OR Kiribati OR Lucia OR Maarten OR Maldives OR Marshall OR Martinique OR Mauritius OR Melanesia OR Micronesia OR Montserrat OR Nauru OR Nevis OR Niue OR Palau OR Papua OR Polynesia OR Principe OR Kitts OR Samoa OR “Sao Tome” OR Seychelles OR Singapore OR Solomon OR Suriname OR Timor-Leste OR Tonga OR Trinidad OR Tobago OR Tokelau OR Turks OR Tuvalu OR “Puerto Rico” OR Marianas OR Martinique OR Vanuatu OR Verde OR Vincent OR “Virgin Island$”

Full search:
(A) AND (B)
