# Supplementary material for: Examining Food Sources and Their Interconnections over Time in Small Island Developing States: A Systematic Scoping Review
Source: Nutrients. 2025 Jul 18;17(14):2353. doi: 10.3390/nu17142353 (PMC12298424; doi:10.3390/nu17142353)
Supplement: Supplementary file 1 [file nutrients-17-02353-s001.zip › WPRIM database_search strategy.pdf]

Search strategy: WPRIM database

https://pesquisa.bvsalud.org/gim/advanced/?lang=en via Global Index Medicus

443 hits retrieved on 18 Jun 2021 (no limits applied)

Combination of Keywords and Subject Heading.

- Advanced Search
- By default TW: = Title + Abstract + DeCS/MeSH terms
- Apply filters - Database selection: WPRIM

Just kept the General terms + Context, as per Isla Kuhn comments on this database

tw:((tw:((food environment OR nutrition environment OR food source OR food sourcing OR food purchasing OR dietary pattern OR food consumption OR food choice OR food preference OR market-based food))) AND (tw:((anguilla OR antigua OR antilles OR aruba OR bahamas OR barbuda OR barbados OR belize OR bermuda OR caicos OR caledonia OR cayman OR comoros OR "cook islands" OR cuba OR curacao OR dominica OR dominican OR fiji OR grenada OR grenadines OR guadeloupe OR guam OR guinea-bissau OR haiti OR jamaica OR kiribati OR lucia OR maarten OR maldives OR marshall OR martinique OR mauritius OR melanesia OR micronesia OR montserrat OR nauru OR nevis OR niue OR palau OR papua OR polynesia OR principe OR kitts OR samoa OR "sao tome" OR seychelles OR singapore OR solomon OR suriname OR timor-leste OR tonga OR trinidad OR tobago OR tokelau OR turks OR tuvalu OR "puerto rico" OR marianas OR martinique OR vanuatu OR verde OR vincent OR "virgin-islands")))) AND (collection_gim:("WPRIM"))
